# Piloting “From the Inside Out” — a toolkit addressing tuberculosis-related self-stigma

**DOI:** 10.1186/s44263-024-00062-5

**Published:** 2024-06-01

**Authors:** Stephen H.-F. Macdonald, Nadine Ferris France, Ian Hodgson, Fadhil Ali, Christa Dewi, Iman Abdurrakhman, Yeremia Mozart Runtu, Alva Juan, Jhon Sugiharto, Elaine Byrne, Ronan M. Conroy

**Affiliations:** 1 School of Medicine, University of Limerick, Limerick, V94 T9PX Ireland; 2 Beyond Stigma, 18A Redleaf Business Park, Turvey Avenue, Donabate, Co., Dublin, Ireland; 3 School of Public Health, University College Cork, Cork, Ireland; 4 DocDoc Pte. Ltd., Jl. Harsono RM No.22A, RT.007 RE004/RW.4, Ragunan, Ps. Minggu, Kota Jakarta Selatan, Daerah Khusus Ibukota Jakarta 12550 Indonesia; 5 Center for Tropical Medicine, Universitas Gadjah Mada, Jl. Medika, Senolowo, Sinduadi, Mlati, Sleman, DIY 55281 Indonesia; 6 Jaringan Indonesia Positif (JIP), Jl. Kudus No.16 RT 08/06, Dukuh Atas, Menteng, Kec., Menteng, Kota Jakarta Pusat, Daerah Khusus Ibukota Jakarta 10310 Indonesia; 7 Yayasan KNCV Indonesia (YKI), Altira Business Park, Jl. Yos Sudarso No.12-15, Sunter Jaya, Kec. Tj. Priok, Jkt Utara, Daerah Khusus Ibukota Jakarta 14360 Indonesia; 8 Center for Positive Health Sciences, Royal College of Surgeons in Ireland (RCSI), 123 St Stephen’s Green, Dublin 2, D02 YN77 Ireland; 9 Department of Public Health and Epidemiology, Royal College of Surgeons in Ireland (RCSI), 123 St Stephen’s Green, Dublin 2, D02 YN77 Ireland

**Keywords:** Self-stigma, Stigma, Tuberculosis, Psychosocial interventions, Capacity-building, Indonesia

## Abstract

**Background:**

Self-stigma among people who have tuberculosis (TB) can contribute to non-adherence to medication and disengagement from care. It can manifest in feelings of worthlessness, shame, and guilt, leading to social withdrawal and disengagement from life opportunities. Self-stigma may also affect families of those who have TB, or healthcare workers who treat them. However, few interventions addressing TB self-stigma exist to date.

**Methods:**

We piloted the delivery of a toolkit of psychosocial interventions using a “training-of-trainers” approach with six staff members of a TB-focused NGO (Non-Governmental Organisation) and partner organisations in Jakarta, Indonesia. These trainers could then disseminate the toolkit among community partner organisations. Local staff involvement throughout the study supported translation and adaptation to enhance cultural and language appropriateness. Over a 2-day training-of-trainers workshop, the NGO staff were familiarised with the mode of delivery of the toolkit, which they then delivered via a four-day participatory workshop with 22 people who have TB/TB survivors, who were representatives of partner organisations working among communities affected by TB.

**Results:**

The newly-trained local facilitators delivered the toolkit to the participants, who self-reported significant increases in knowledge and efficacy around TB self-stigma post-intervention compared to baseline (*Z* = 1.991, *p* = 0.047, Wilcoxon signed-rank test). The participants’ levels of self-compassion were also significantly higher post-workshop (*Z* = 2.096, *p* = 0.036, Wilcoxon signed-rank test); however, these effects were not maintained at 3-month timepoint. There was also a significant increase post-workshop in one of the participants’ Ryff dimensions of psychological wellbeing, that of positive relationships with others (*Z* = 2.509, *p* = 0.012, Wilcoxon signed-rank test) but this was also not maintained at the 3-month timepoint.

**Conclusions:**

The observed changes in recipients’ self-reported levels of knowledge and efficacy, self-compassion, and psychological wellbeing may warrant further investigation into the best modalities for toolkit delivery (frequency, dose, duration) and support for individuals as they progress through the TB treatment journey.

**Supplementary Information:**

The online version contains supplementary material available at 10.1186/s44263-024-00062-5.

## Background

Approximately 1.3 million people die due to tuberculosis (TB) annually, with around one-quarter of the world’s population infected with *M. tuberculosis*, of whom up to 10% will go on to develop active TB disease [1]. Active TB is often also found among people who have HIV and whose immune systems have been diminished through AIDS. Among the annual deaths of 2022 due to TB, 167,000 were people with HIV [1–3]. TB is initially treated with first-line antibiotics such as isoniazid and rifampin, although improper adherence to treatment regimens has led to the rise of drug-resistant, multidrug-resistant, and extensively drug-resistant TB which are of growing concern globally [4].

Various medical and social factors can negatively impact TB treatment adherence and outcomes [5, 6]. One key example of such factors is stigma, which has various consequences including delayed treatment-seeking, avoidance of disclosure of TB status, poor treatment adherence, and poor quality of life [7–10]. Additionally, TB may exert downstream effects even after treatment completion — for example common post-TB sequelae, such as neurological and cardiovascular impairment, as well as reduced social and psychological wellbeing, have an impact beyond resolution of the disease itself, and stigma can persist even after treatment has been completed [11, 12]. Successful policies to eliminate TB and prevent drug resistance must therefore address such factors, with stigma and self-stigma (or internalised stigma) being noted as important targets that are relatively under-explored in terms of intervention [13, 14].

Self-stigma is a complex phenomenon that is driven by multiple interacting societal, contextual, and self factors [15–17]. Self-stigma is distinct from external stigma and discrimination in that it concerns beliefs, feelings, and actions that an individual holds about, and does to, themselves rather than being something which is believed about, or is done to, someone else [18]. The various manifestations of self-stigma — disempowerment, guilt, shame, and social withdrawal, as well as lack of engagement in care — remain constant across a wide range of stigmatised health conditions [18–20]. Notable among these medical conditions are mental illness, infectious diseases such as HIV and hepatitis C, and noncommunicable conditions such as obesity [21–23]. TB is no exception [20].

Tools such as the Health Stigma and Discrimination Framework may be useful in identifying intervention points [24]. TB stigma arises, like other health-related stigmas, against a backdrop of underlying drivers and facilitators, such as stereotypes, prejudice, and cultural norms [24]. “Marking” of individuals who have a stigmatised condition occurs, leading them to become targets of the manifestations of stigma, such as stigmatising attitudes and discrimination [24]. With this in mind, interventions addressing TB stigma should be timely and appropriately targeted. Nutall et al. in their 2022 review noted that stigma interventions typically centred around education and psychosocial support, to counter the drivers and facilitators of stigma, variously targeting people who have TB, health workers, and the public [13]. Similarly, Foster et al.’s scoping review of interventions categorised stigma interventions as: (1) combined individual and interpersonal level interventions, targeting individual behaviour and that of the individual’s family and personal network, through actions such as home visits, family workshops, and support from community leaders; (2) interpersonal level interventions to reduce enacted stigma and improve knowledge around TB; (3) organisational level interventions to improve health workers’ knowledge and practice; and (4) community level interventions to improve the public’s knowledge around TB, and to reduce stigmatisation [14].

People who have stigmatised medical conditions such as TB, as well as TB survivors and even those involved in their care, may be stigmatised and discriminated against both in society in general and in the health system [9, 25]. Juniarti and Evans noted, in their 2011 qualitative review of stigma around TB, that TB is seen as a ‘dirty’ disease, bringing with it manifestations of external stigma such as social shunning of those who have TB and their families, as well as manifestations of self-stigma such as shame and withdrawal [7]. In combination, these may contribute to delays or disruptions in diagnosis and treatment [26]. It is likely that addressing self-stigma will contribute towards improvements in TB care outcomes, but there is a limited range of interventions to achieve this [27, 28].

In terms of practice, educational toolkits can be a useful approach for rapidly building collective knowledge, understanding, and attitudes around aspects of service delivery, treatment, or care [29]. Furthermore, the training-of-trainers approach is useful in supporting this type of knowledge dissemination, particularly where given adequate resources and support for continuation [30, 31]. This approach can be useful in terms of scalability where staff and teams are not necessarily required to leave service for extended periods to undertake centralised training and instead can absorb the materials and approach trainer colleagues for help and advice in situ. We adopted this approach by firstly training local facilitators to deliver the toolkit via a training-of-trainers approach, then these newly-trained local facilitators delivered the toolkit materials to TB patients and survivors via a participatory workshop, with the aim of creating changes in participants’ self-reported efficacy and knowledge around self-stigma.

Indonesia was chosen opportunistically as the pilot location, due to the strong links between KNCV Tuberculosis Foundation (https://www.kncvtbc.org/en/) and the Indonesian KNCV network member, Yayasan KNCV Indonesia (YKI) (https://yki4tbc.org/), who are partnered with local community organisations around the country working with people who have TB, and TB survivors. The project was funded by the Stop TB Partnership as part of Round Eight of their Challenge Facility for Civil Society grants [32], which seek to test integrative responses to TB that focus on the patient, and are linked with community and civil society.

Indonesia has an estimated annual TB incidence of 354 per 100,000 population and an estimated incidence of drug-resistant TB of 10 per 100,000 [33]. Indonesia remains among the top 30 TB high-burden countries and is one of the eight countries that accounted for two-thirds of new TB cases in 2017 [34]. The country’s National TB Program has cited a number of challenges that are faced in the Ministry of Health’s mission to eliminate TB in the country by 2030, including lack of knowledge around TB, limited involvement of patients and community in TB control, lack of public awareness around the rights and responsibilities of TB patients, and high levels of stigma [35]. A significant number of patients with HIV and MDR-TB (multidrug-resistant TB) refuse treatment because of stigma and fears of side effects, which are insufficiently addressed by health staff [36], adding to the risk of transmission. Furthermore, stigmatising attitudes and practices among health workers may also contribute to disengagement from care and reduce treatment adherence [37]. To address these challenges the engagement of communities and support organisations, and building their capacity to support their members in the places where they perceive the greatest need, may yield a positive impact as has been suggested for other pandemic infections such as HIV [38].

In this context, we sought to target the individual and interpersonal levels, aiming to build capacity for individuals to counter the negative effects of stigma. This article focuses on the piloting of a toolkit of interventions that aim to address TB self-stigma. We aimed to address the main research question: *Can the TB self-stigma toolkit improve participants’ knowledge and efficacy around self-stigma?*

## Methods

### Toolkit against TB self-stigma

A toolkit of anti-self-stigma interventions [39] was created as a collaboration between Beyond Stigma (https://www.beyondstigma.org/ formerly The Work for Change), an Irish NGO focusing on research and interventions against self-stigma [16, 22, 39] and KNCV Tuberculosis Foundation — a Dutch international NGO focusing on care for individuals living with TB and TB survivors (https://www.kncvtbc.org/en/) [40]. The same collaboration has also previously contributed to guidance around the measurement of self-stigma among people living with TB [41]. The toolkit draws on examples of techniques deployed by frontline NGOs working across fields including mental health, HIV and AIDS care, and others [39] and it was made available online as a resource for community-based organisations, NGOs, and National TB Programmes to roll out to their constituents. At the time of writing, the toolkit has not been reviewed or published in a peer-reviewed journal, however during its development, the toolkit was reviewed by experts in the field, as well as TB survivors and community members [42]. The toolkit consists of eight modules to familiarise workshop participants with aspects of self-stigma, discrimination, and the TB treatment journey. A major focus of the materials is on participation, discussion, and joint learning through varied exercises to ensure full embedding of knowledge on self-stigma and the range of interventions and activities that can be used to impact the effects of TB self-stigma. (See Additional file 1 for the full toolkit.) The toolkit has also been piloted with NGOs and participants in Kazakhstan, the Philippines and Vietnam, but has not yet been the focus of a research publication.

### Content of the intervention

The toolkit itself uses the socio-ecological model [43] as a basis for addressing self-stigma and comprises eight modules as follows:

- Module 1: What is self-stigma? An introduction to the concept of self-stigma.
- Module 2: Dealing with self-stigma and shame: explores self-stigma and shame, enabling participants to learn how to identify and cope with the thoughts and feelings.
- Module 3: DR-TB (drug-resistant TB): explores the impact of DR-TB on self-stigma.
- Module 4: Infection control and self-stigma: self-stigma in the context of infection control.
- Module 5: Health rights, TB, and self-stigma.
- Module 6: Treatment: linkage between treatment for TB and self-stigma.
- Module 7: Planning for the future — TB free! What now?
- Module 8: Evaluation of self-stigma and its impact.

The toolkit combines a number of approaches including a technique called self-inquiry (adapted from Inquiry-based Stress Reduction), a cognitive, mindfulness-based approach that supports people to identify and question their negative core beliefs [44]. The toolkit uses discussion, drawing, games, role-play, quizzes and informational sessions to walk participants through the modules. Guidelines for selection of participants as well as selection of facilitators are included in the toolkit.

### Delivery of the intervention

**Fig. 1:**
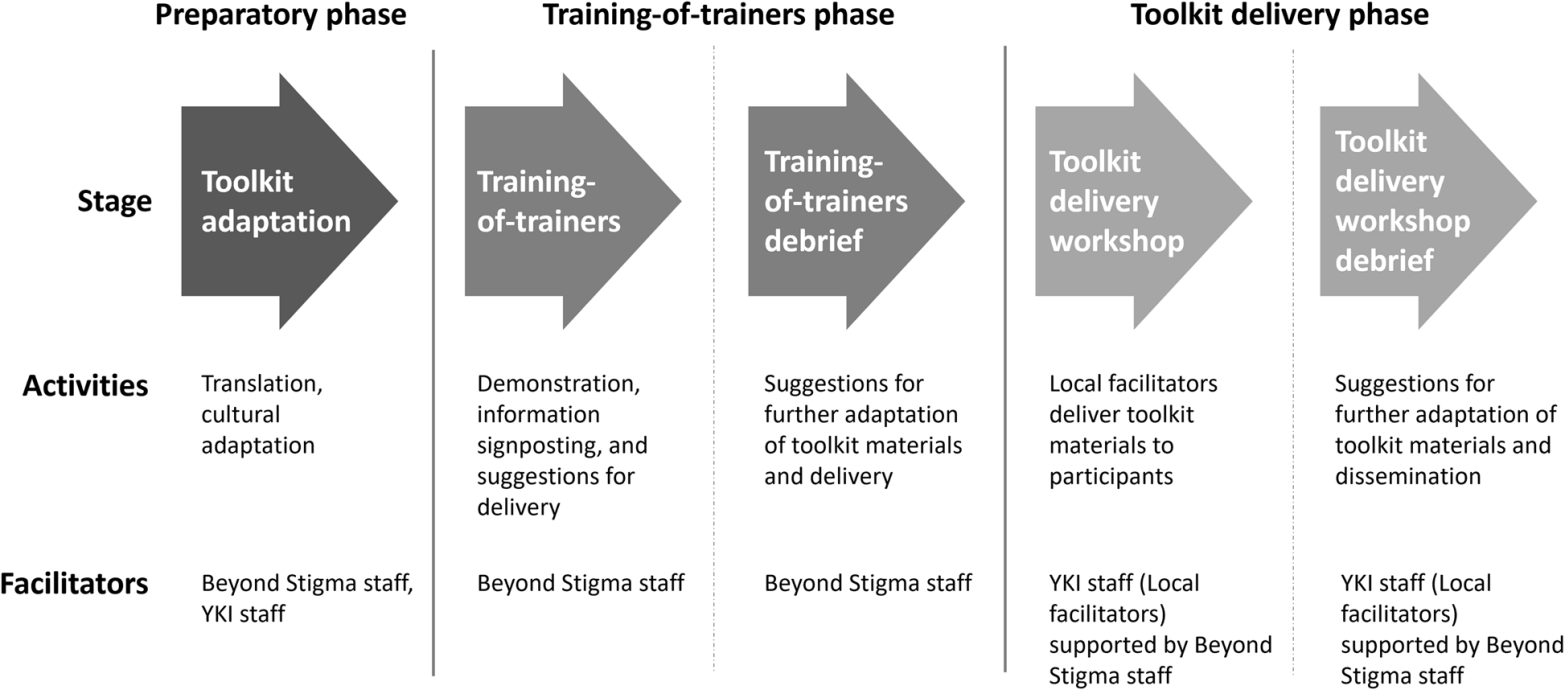
Description of the stages of the intervention. Showing stages of the intervention (grey arrows), and indicating the activities conducted at each stage, in addition to the relevant facilitators who delivered content at those stages

The intervention took place across through three distinct phases: (1) a preparatory phase of language and cultural adaptation of the toolkit, (2) a 2-day training-of-trainers workshop to familiarise local facilitators with the toolkit delivery, followed by a debrief to gather suggestions for further adaptation of toolkit and workshop materials, and (3) A 4-day workshop to deliver toolkit materials to TB patients and TB survivors, followed by debrief to gather feedback and suggestions for future adaptation and dissemination (Fig. 1). The preparatory phase was conducted remotely, whilst the training-of-trainers and 4-day participant workshop were conducted at a hired conferencing facility in a hotel in Jakarta, Indonesia, from the 19th to 24th November 2018.

During the preparatory phase, in order to ensure culturally sensitive and appropriate delivery of the toolkit materials by the newly-trained local facilitators, YKI and Beyond Stigma staff collaborated extensively to prepare for the workshops. The toolkit materials and facilitator presentations were translated into Bahasa Indonesia in advance of the sessions, with adjustments made to phrasing and tone using the cultural sensitivity of the local team. Accommodations were made to facilitate accessibility for participants with hearing loss or literacy challenges, such as having a local facilitator assigned to those participants to assist with interpretation.

Next, five staff of YKI and partners (authors FA, CD, IA, YMR, and AJ) were familiarised with the contents and mode of delivery of the majority of the toolkit components, during a 2-day (9am–5 pm) training-of-trainers workshop, facilitated by three Beyond Stigma staff (authors SM, IH, and NFF). These local facilitators were selected to undertake the training-of-trainers workshop due to their field experience in working with community organisations supporting people who have TB. The training-of-trainers session focused on showing examples of the content of the toolkit, suggestions on how to deliver them, and working through exercises contained in it, with the local facilitators fulfilling dual roles as recipients of the toolkit and evaluators of its usefulness. This process, and subsequent debriefing session, led to further refinements of the toolkit and workshop materials before it was rolled out to participants over a 4-day workshop.

The local facilitators then worked with Beyond Stigma facilitators to configure and deliver the 4-day workshop, also hosted in Jakarta, to deliver the toolkit to 22 participants, with a ratio of 4.4 participants to 1 facilitator. The participants were people who have drug-sensitive TB, and TB survivors, who were working as community advocates among YKI’s local partner organisations POP TB (Perhimpunan Organisasi Pasien TB), PETA (Pejuang Tangguh), Terjang, LKNU (Lembaga Kesehatan Nahdlatul Ulama), and TB-HIV Care Aisyiyah Center (Aisyiyah), drawn from Jakarta and West Java. These participants were opportunistically selected by YKI staff based on their willingness to participate in this pilot study. All were adults aged 18 years or older. The participants were not screened for self-stigma beforehand, and this was not a selection criterion for participation. The local facilitators adapted materials and created presentations in Bahasa Indonesia and conducted the workshop sessions mostly in that language. Local facilitators provided translation support for Beyond Stigma to aid communications where necessary. Additionally, following the 4-day workshop, a debriefing session was conducted with the participants to identify issues or additional improvements that could be made and implemented in future. Here, the local facilitators, who were themselves experienced in working with community organisations supporting people who have TB, acted as moderators and also gave suggestions for refinement.

### Measurement of intervention outcomes

We assessed our primary outcome, participants’ self-reported knowledge and efficacy around self-stigma, as well as secondary outcomes of self-compassion, self-stigma, and psychological wellbeing via questionnaires with responses entered using 7-point Likert-type scales. For self-reported knowledge and efficacy, we created a brief questionnaire which asked participants to self-rate their agreement with statements such as “I know what self-stigma is” accompanied by statements where they could give free text responses, such as “I can give examples of the manifestations of self-stigma” (for full questionnaire, see Additional File 2). For self-compassion, we used the Neff Self-Compassion scale [45], for self-stigma we used the Van Rie Patient Perspectives Towards Tuberculosis scale [46], and for psychological wellbeing, we used the Ryff dimensions of psychological wellbeing [47].

### Data analysis

For analysis, participants’ responses to the self-efficacy and Neff Self-Compassion scales were averaged to produce single scale scores, and items from the Van Rie Patient Perspectives Towards Tuberculosis scale were divided into categories of Disclosure (items 5, 7, 9, and 12 presented in our questionnaire in Additional File 2), Isolation (items 1,2,3, and 6 presented in our questionnaire in Additional File 2), and Guilt (items 8, 10, and 11 presented in our questionnaire in Additional File 2) reflecting the result of factor analysis by Fuady et al. [48]. For the Ryff dimensions, wellbeing responses were summed in each of the six dimensions (Autonomy, Environmental mastery, Personal growth, Positive relations with others, Purpose in life, and Self-acceptance).

Incomplete questionnaires were handled as follows: (A) Cases with more than one missing response were excluded from the analysis of that scale. (B) In the Neff Self-Compassion scale, three participants at baseline and one at the post-intervention timepoint missed five of 12 questions which were on the back of the questionnaire page. We tested whether the average score on the first seven questions for all participants was different to that of the remaining five for those who completed the full questionnaire, finding no significant difference. We therefore analysed the dataset using the affected participants’ average score in the first seven questions as a proxy for their total score. (C) Some items in our self-reported knowledge and efficacy around the self-stigma scale asked participants to rate their agreement with a statement and add detail in free text. Some participants included detailed and clearly positive responses in the free text but did not enter a response on the Likert-type scale. In such cases, we assigned the most conservative positive value (i.e. “slightly agree”). We analysed the data using the Wilcoxon Signed Rank Test comparing baseline with post-test and 3-month follow-up, reporting baseline median, median difference + interquartile range (IQR), *Z* score, effect size, and *p*-value [49]. Statistical analysis was performed using IBM SPSS Statistics version 28.0.0.0. Raw questionnaire response data is contained in Additional file 3.

## Results

During the 2-day training-of-trainers workshop, Beyond Stigma staff signposted important content from the toolkit, such as structure and suitability for use with participants at different stages of the TB treatment journey, as well as simulating key exercises. Having undertaken this training, the YKI staff members were able to deliver the toolkit over a 4-day workshop to the 22 community participants with minimal input or assistance from Beyond Stigma staff.

### Primary outcome: self-reported knowledge and efficacy around self-stigma

Participants’ self-reported knowledge and efficacy around self-stigma was significantly improved at post-intervention compared to baseline (*Z* = 1.991, *p* = 0.047) but this effect was not maintained at 3-month timepoint (*Z* = 1.823, *p* = 0.068) (Table 1).

**Table 1:** Analysis of participant responses to questionnaire items on self-reported knowledge and efficacy around self-stigma

		**Post-workshop**				**Three-month follow-up**			
	**Baseline median**	**Median difference (IQR)**	***Z***	**Effect size (*r*)**	***p*-value**	**Median difference (IQR)**	***Z***	**Effect size (*r*)**	***p*-value**
**Self-efficacy**	5.500	0.750 (0.25–1.062)	1.991	0.514	0.047*	0.25 (0.688–0)	1.823	0.577	0.068

Showing baseline median score, median difference and interquartile range, *Z*-score, effect size, and significance level (* = *p* < 0.05). Due to loss to follow-up and missing data, the numbers of respondents varied: at post-workshop *n* = 15; at 3-month follow-up *n* = 10

### Secondary outcomes: self-compassion, self-stigma

The participants’ self-reported levels of self-compassion were also significantly higher post-workshop (*Z* = 2.096, *p* = 0.036); however, this effect was again not maintained at 3-month timepoint (*Z* = 0.357, *p* = 0.721). Participants’ responses to the Van Rie patient perspectives towards tuberculosis scale did not change significantly at either timepoint for Disclosure (*Z* = 0.424, *p* = 0.672 and *Z* = 0.000, *p* = 1.000) and Isolation (*Z* = 0.318, *p* = 0.750 and *Z* = 0.220, *p* = 0.826), and did not change for Guilt post-workshop (*Z* = 1.090, *p* = 0.276). Guilt appeared significantly elevated at the 3-month timepoint (*Z* = 2.115, *p* = 0.034) (Table 2).

**Table 2:** Analysis of participant responses to questionnaire items on Neff Self-Compassion and Van Rie Patient Perspectives Towards Tuberculosis

		**Post-workshop**				**Three-month follow-up**			
	**Baseline median**	**Median difference (IQR)**	***Z***	**Effect size (*r*)**	***p*-value**	**Median difference (IQR)**	***Z***	**Effect size (*r*)**	***p*-value**
**Neff Self-Compassion**	4.875	0.333 (0.083—0.833)	2.096	0.508	0.036*	0.208 (-0.333—0.354)	0.357	0.103	0.721
**Van Rie (Disclosure)**	17.500	0.000 (-3.500 – 2.000)	0.424	0.090	0.672	0.000 (-3.000 – 3.000)	0.000	0.000	1.000
**Van Rie (Isolation)**	18.000	0.000 (-2.000 – 5.000)	0.318	0.068	0.750	0.000 (-4.500—3.500)	0.220	0.057	0.826
**Van Rie (Guilt)**	14.000	-0.500 (-4.000 – 1.750)	1.090	0.232	0.276	2 (-0.500 – 3.000)	2.115	0.546	0.034*

Showing baseline median score, median difference and interquartile range, *Z*-score, effect size, and significance level (* = *p* < 0.05). Due to loss to follow-up and missing data, the numbers of respondents varied: at post-workshop: Neff Self-Compassion *n* = 17; Van Rie *n* = 22. At 3-month follow-up: Neff Self-Compassion *n* = 12; Van Rie *n* = 15

### Secondary outcome: Ryff dimensions of psychological wellbeing

Analysis of psychological wellbeing questionnaire results indicated that after the workshop, there was a significant increase in the participants’ scores in the dimensions of positive relationships with others (*Z* = 2.509, *p* = 0.012). At 3-month follow-up, environmental mastery declined significantly (*Z* = 2.670, *p* = 0.008), whilst self-acceptance was significantly higher than baseline (*Z* = 2.877, *p* = 0.004) (Table 3).

**Table 3:** Analysis of participant responses to Ryff dimensions of psychological wellbeing

		**Post-workshop**				**Three-month follow-up**			
Ryff dimension	**Baseline median**	**Median difference (IQR)**	***Z***	**Effect size (*r*)**	***p*-value**	**Median difference (IQR)**	***Z***	**Effect size (*r*)**	***p*-value**
Autonomy	34.00	2 (− 2.75–5)	1.157	0.273	0.247	0.5 (− 1–2.5)	0.237	0.063	0.812
Environmental mastery	37.00	1 (− 1.00–4.00)	1.482	0.359	0.138	− 2.00 (− 4.00 to − 2.00)	2.670	0.714	0.008**
Personal growth	39.00	1.00 (− 2.50–2.00)	0.176	0.040	0.861	− 3.00 (− 4.50–2.00)	1.426	0.368	0.154
Positive relationships	35.50	2.00 (0.00–4.00)	2.509	0.591	0.012*	− 0.500 (− 1.000–1.750)	0.040	0.011	0.968
Purpose in life	40.00	0.00 (− 2.00–2.250)	0.492	0.123	0.623	− 1.00 (− 2.50–1.50)	0.821	0.212	0.412
Self-acceptance	35.00	2.00 (− 1.00–7.00)	1.706	0.414	0.088	2.00 (1.00–4.00)	2.877	0.769	0.004**

Showing baseline median score, median difference and interquartile range, *Z*-score, effect size, and significance level (* = *p* < 0.05; ** = *p* < 0.01). Due to loss to follow-up and missing data, the numbers of respondents varied — at post-workshop and follow-up: autonomy *n* = 18, *n* = 14, respectively; environmental mastery *n* = 17, *n* = 14, respectively; personal growth *n* = 19, *n* = 15, respectively; positive relationships *n* = 18, *n* = 14, respectively; purpose in life *n* = 16, *n* = 15, respectively; self-acceptance *n* = 17, *n* = 14, respectively

## Discussion

In relation to our primary outcome, participants reported significant increases in knowledge and efficacy around TB self-stigma post-intervention compared to baseline. In relation to our secondary outcomes, participants’ levels of self-compassion were also significantly higher post-intervention, although this was not maintained at the 3-month timepoint. The participants also reported a significant increase in the Ryff dimension of Positive relationships with others post-intervention, but this was again not maintained at the 3-month timepoint. Furthermore, at the 3-month timepoint (but not at the post-intervention timepoint), there appeared to be a significant increase in the Ryff dimension of Self-acceptance and a significant decrease in Environmental mastery, as well as a significant increase in the Van Rie Patient Perspective scale relating to guilt.

Unfortunately, whilst TB can be successfully treated by medication, the high rate of loss to follow-up, ongoing transmission, and growing incidence of drug-resistant and multidrug-resistant disease indicate the need for additional mechanisms to support patients during the treatment journey [50–52]. Alipanah et al. in their 2018 systematic review and meta-analysis of adherence interventions noted that treatment outcomes can be supported by patient education and psychological interventions [53]. Similarly, our toolkit aims to aid in engagement with health services, and treatment adherence, by providing education and psychosocial intervention tools addressing self-stigma and shame, which can harm patients’ ability to complete the treatment journey.

We set out to train a small group of NGO staff to deliver the materials of the toolkit against self-stigma with a small group of people who have TB, and TB survivors, drawn from community networks in Indonesia, responding to the need for community-based and person-centred interventions to support TB care and outcomes. The local facilitators were able to deliver the toolkit materials to the participants during the 4-day workshop with minimal assistance or input from Beyond Stigma staff, suggesting that the toolkit’s *conceptual* content could be easily understood and adapted. The pre-translation of the full toolkit into Bahasa Indonesia before training began most likely aided this process to a certain degree although the local facilitators were all fully fluent in English. However, their cultural competency, fluency in the local idiom, and familiarity with motivating and engaging community members were most likely the key factors that helped transfer the concepts to the workshop participants.

Exposure to the toolkit materials through taking part in the interactive workshop over 4 days appears to have had the positive effect of increasing participants’ self-reported knowledge and efficacy around TB self-stigma. Although the effect was not maintained at 3-month follow-up, the transfer of knowledge and skills about self-stigma and how to deal with self-stigma could be supported by refresher training. Further investigation would help us understand the best way to enable participants to maintain their knowledge, or to help embed the knowledge and skills in their daily practice. For example, are they willing to revisit workshop materials by themselves? Could they conduct self-stigma workshops with their own communities? What further training or additional information about self-stigma would they need? Since the toolkit is aimed at empowering users with knowledge and techniques to help those experiencing self-stigma, maintaining that knowledge and efficacy will be essential for sustainability. In light of the experience reported above, we suggest a cautiously positive answer to our research question — *can the TB self-stigma toolkit improve participants’ knowledge and efficacy around self-stigma?* It will also be crucial to examine the way that participants’ knowledge and understanding of self-stigma, and ways to address it, evolves over time. For example, starting with a general understanding of stigma and its concepts as delivered by the TB self-stigma toolkit workshops, do the participants then integrate their own experiences and knowledge from their formal and informal community networks to adapt that information to support themselves and others? It is also difficult to predict the frequency and dosage of such interventions that would be required to sustain change.

When we examined whether the TB self-stigma toolkit could influence other relevant measures including levels of self-compassion, patient perspectives towards self-stigma, and psychological wellbeing, our results were mixed. A positive outcome was that we observed that participants’ self-reported levels of self-compassion were significantly improved following the workshop. This in itself is a valuable finding since self-compassion may act as a buffer against self-stigma [23, 54]. Although this effect was not maintained at the 3-month timepoint, repeat exposure and refresher training may help to reinforce the short-term improvements. Indeed, during the workshop feedback and debrief session, the participants indicated a desire for repeated sessions in order to continue their learning and familiarisation with the materials. Due to time and funding constraints, the workshop intervention could only be delivered once, and future projects could aim towards more continuous exposure to the toolkit materials — for example using them during regular meetings of patient support groups such as TB clubs, or encouraging participants to track their use via “treatment journals” where they record the activities conducted, and effects, over a period of time.

Participants’ responses to the Van Rie patient perspectives towards the tuberculosis scale did not change significantly at either post-workshop or 3-month timepoints for Disclosure or Isolation, or Guilt at the post-workshop timepoint. Guilt appeared to be significantly higher at 3-month follow-up. Two considerations are important: Firstly, results may have been influenced by the wording of the questionnaire: Participants were asked questions such as "Some people who have TB feel hurt by how others react to knowing they have TB". This was done out of cultural consideration in the context of avoiding directly asking sensitive questions. The third-person wording of the questions was confusing to participants, and during feedback sessions, they reported that they had been uncertain whether they should answer from their own perspective or in the third-person. First-person wording of the Van Rie scale would therefore appear to be preferable, as recommended by Redwood et al., in their adaptation of the Van Rie scale for Vietnam, and Fuady et al., in adaptation and validation of the scale for Indonesia, and we suggest that the latter should be used for all future studies in this area, to overcome the challenges that we had observed in our study [48, 55]. These results may therefore not be useful in understanding whether the workshop changed the participants’ own levels of self-stigma. However, they may suggest that the participants perceived self-stigma around TB to exist among others that they have encountered — i.e. if they did not think that self-stigma existed in their communities, a low baseline median score for the Van Rie items would be expected. Secondly, due to their being already engaged in TB support programmes with community organisations, the participants to the workshop were the “educated audience” — i.e. already undergoing treatment and connected with support. Their experience of stigma and self-stigma would likely be different to those who were recently diagnosed or had just started treatment, for example.

We also observed potential improvements in one dimension of psychological wellbeing — positive relationships with others, which improved post-workshop. Psychological wellbeing is linked with resilience against self-stigmatisation [56], and this may warrant further investigation. We also observed an increase in guilt, as well as self-acceptance, in addition to a decline in environmental mastery at the 3-month timepoint only, with no difference in these immediately post-workshop. The low participant numbers, and lack of information on participants’ circumstances following their engagement in the workshop, raise the possibility that any changes that were NOT observed immediately following the intervention, but were observed at the 3-month follow-up, are spurious correlations and it is difficult to draw any meaningful insight from these. Further research, such as a randomised controlled trial with much larger numbers of participants, and detailed tracking of how the participants were using or applying the toolkit materials/information, would be required to strengthen our knowledge in this regard.

Regarding future use of the TB self-stigma toolkit, this is freely available as a resource from the KNCV Tuberculosis Foundation website. However, its use, and sustainability of use in primary care or community settings, is dependent on several factors: Although the toolkit itself is free, staff or volunteer time is needed, as is a venue to run workshops, and various consumables such as printed materials, stationery, and IT resources are also required. However, beyond these requirements, this low-tech approach may be a useful means of disseminating information and techniques among communities [29].

In terms of limitations, this was a pilot study without a control group, with low numbers of participants, and practical difficulties with missing data and loss to follow-up. In particular, the study had low power to detect changes in the self-completion measures. Given the number of observations available, the study had a significant statistical power (90%) to detect only changes of 0.9 standard deviations on a continuous measure, corresponding to a large treatment effect — under such a scenario, over 80% of participants would be expected to change for the better. The findings cannot yet be broadly generalised. The workshop participants were drawn from local community organisations and were already at an advanced stage of their treatment journeys, as well as being involved in community support groups for TB. Their experience of learning the concepts of self-stigma in TB, and techniques to overcome it, may be different from those who have not been involved in such work, or who are only just beginning treatment. The latter may find it difficult to engage in discussions about their personal feelings and beliefs, therefore future studies should seek to not only test the toolkit materials, but also examine the best ways to ensure that new (and possibly marginalised) participants are given adequate support around participation. The scale we used to assess participants’ knowledge and efficacy around self-stigma was created for this study and should be further investigated in terms of internal consistency and reliability. To minimise missing data, future research must reinforce the monitoring of participants’ completion of instruments, as well as examining reasons for participants becoming lost to follow-up.

## Conclusions

It was possible to rapidly train a small group of local facilitators in the methods and delivery of a new toolkit that aims to empower end-users with knowledge and techniques to overcome TB self-stigma. These local facilitators were then able to deliver the materials via workshop sessions to people who have TB, and TB survivors, with minimal support, suggesting that this approach is a convenient means to rapidly disseminate intervention knowledge and techniques. We noted improvements in self-reported knowledge and efficacy around self-stigma, self-compassion, and certain measures of psychological wellbeing, among participants. Further research is required to understand the effects of the toolkit in terms of dosage and frequency of exposure, and its direct effect on self-stigma among workshop participants must also be investigated.

## Supplementary Information

**Additional file 1.** “From the inside out: Dealing with TB-related stigma and shame.” A pdf version of the toolkit of psychosocial interventions against TB Self-Stigma developed by Beyond Stigma for KNCV Tuberculosis Foundation.

**Additional file 2.** Excel spreadsheet containing the questionnaire items deployed to participants during this study.

** Additional file 3.** Raw data responses from participants which was analysed for Tables 1 and 2.

## Data Availability

The dataset supporting the conclusions of this article are included within the article and its additional files.
