# Supplementary material for: Piloting “From the Inside Out” — a toolkit addressing tuberculosis-related self-stigma
Source: BMC Glob Public Health. 2024 Jun 1;2:31. doi: 10.1186/s44263-024-00062-5 (PMC11622890; doi:10.1186/s44263-024-00062-5)
Supplement: Supplementary file 1 — Additional file 1. “From the inside out: Dealing with TB-related stigma and shame.” A pdf version of the toolkit of psychosocial interventions against TB Self-Stigma developed by Beyond Stigma for KNCV Tuberculosis Foundation. [file 44263_2024_62_MOESM1_ESM.pdf]

TABLE OF CONTENTS

Introduction 10
Purpose 10
Structure 12
Delivery 13
Package snapshot 16
Glossary 23

MODULE 1: WHAT IS SELF-STIGMA? 25
Introduction 26
Part I - Welcome participants to the session [15 mins.] 28
Part II - Introduction to this session: What is self-stigma? [15 mins.] 29
Part III - Exercises 31
Exercise 1.1 Understanding self-stigma from end-to-end [30 mins.] 31
Exercise 1.1b Living with self-stigma [15-30 mins.] 32
Exercise 1.2 Recognizing TB self-stigma and its challenges [60 mins.] 33
Exercise 1.3 Recognizing self-stigma in the broader context of well-being [60 mins.] 37
Ryff dimensions 39
Part IV - Session wrap [30 mins.] 40
Resources 40

MODULE 2: DEALING WITH SELF-STIGMA AND SHAME 41
Introduction 42
Exercise 2.1 Keeping a TB Journal <TB/DR-TB> 44
Exercise 2.2 Being breathed <TB/DR-TB> 45
Exercise 2.3 Universality, self-stigma, and shame <TB/DR-TB> 46
Exercise 2.4 What I think you think about me <TB/DR-TB> 47
Exercise 2.5 Who is judging whom? <TB/DR-TB> 49
Exercise 2.6 How TB affects me? <TB/DR-TB> 51
Exercise 2.7 Do you believe everything you think? “I have TB and that means that….” <TB/DR-TB> 52
Exercise 2.8 Living self-stigma <TB/DR-TB> 54
Exercise 2.9 My journey - visualizing TB and stigma <TB/DR-TB> 56

Exercise 2.10 My right to tell <TB/DR-TB> 58
Exercise 2.11 Staying in your own business: reducing stress <TB/DR-TB> 59
Exercise 2.12 My agency, my power <TB/DR-TB> 60
Resources 61

MODULE 3: DRUG-RESISTANT TB (DR-TB) AND SELF-STIGMA 63
Introduction 64
Part I - Background presentation [see PowerPoint slides] 65
Part II - Exercises 67
Exercise 3.1 The many faces of DR-TB <TB> 67
Part III - Session wrap 68
Resources 68

MODULE 4: TRANSMISSION CONTROL AND SELF-STIGMA 69
Introduction 70
Part I - Background presentation [see PowerPoint slides] 71
Part II - Exercises 72
Exercise 4.1 TB lifeline <TB> 72
Exercise 4.2 What do we know about TB and risk? 73
Part III - Session wrap 74
Resources 74

MODULE 5: HEALTH RIGHTS, TB, AND SELF-STIGMA 75
Introduction 76
Part I - Background presentation [see PowerPoint slides] 77
Part II - Exercises 79
Exercise 5.1 Rights and the patient with TB <TB> 79
Exercise 5.2 Perfectly Imperfect 80
Part III - Session wrap 82
Resources 82

TABLE OF CONTENTS

MODULE 6: TB TREATMENT AND SELF-STIGMA 83
Introduction 84
Part I - Background presentation [see PowerPoint slides] 85
Part II - Exercises 87
Exercise 6.1 TB self-stigma and treatment <TB> 87
Exercise 6.2 Treating my body, treating my mind <TB> 88
Part III - Session wrap 90
Resources 90

MODULE 7: PLANNING FOR THE FUTURE - TB FREE! WHAT NOW? 91
Introduction 92
Part I - Background presentation [see PowerPoint slides] 93
Part II - Exercises 94
Exercise 7.1 The end of my TB journey - moving on <TB> 94
Exercise 7.2 Accompaniment: being a TB Champion 95
Part III - Session wrap 96

MODULE 8: MONITORING & EVALUATION OF THIS INTERVENTION 97
Introduction 98
8.1 Evaluating the effectiveness of this intervention 98
8.2 Monitoring implementation of the intervention 105
References 107

ANNEXES 111
Annex 1 – Additional resources, interventions and ideas 112
Annex 2 – Sample agendas 115
Annex 3 – Self-stigma learning tool 1: Defining self-stigma 118
Annex 4 – Self-stigma learning tool 2: We are not alone 119
Annex 5 – Example participant profiles for Exercise 1.3 120
Annex 6 - Journaling template for Exercise 2.1 121
Annex 7 - Labels for Exercise 2.8 – Living Self-Stigma 122
Annex 8 - Handout for Exercise 2.10 – My right to tell 123
Annex 9 - Handout for Exercise 2.10 - Where am I on the disclosure continuum? 124

Annex 10 - Handout for Exercise 2.11 - Staying in your own business 125
Annex 11 - Case studies for Exercise 3.1 – the many faces of TB 126
Annex 12 – TB quiz for Exercise 4.2 128
Annex 13 – Case studies for Exercise 5.1 – Rights and the patient with TB 134
Annex 14 - Card game for Exercise 5.2 – Perfectly imperfect 135
Annex 15 - Example baseline-endline assessment of particip 147
Annex 16 - Example facilitator pilot feedback form 149
Annex 17 - Example participant workshop feedback form 150

Introduction

"Don't believe Everything You Think."

– Byron Katie

Purpose

Goal: "From the Inside Out" is designed to help individuals identify, understand, and address self-stigma and anticipated stigma. We provide a framework and tools to reduce self-stigma in people with tuberculosis (TB). In order to explore self-stigma and examine its impact people must reach solutions. In particular, the package aims to challenge and overcome self-stigmatizing beliefs to improve well-being and ensure affected people can lead productive lives that are free of self-judgment

Participants who go through the whole intervention will come out with:

- A firm understanding of what self-stigma is.
- An ability to spot self-stigma in their own lives.
- Recognition of the powerful effects of self-stigma on well-being.
- The knowledge necessary to overcome self-stigma.
- Techniques to overcome self-stigma.
- Greater self-compassion and compassion for others.
- Techniques to address anticipated stigma.
- A fuller understanding of their rights as people with TB.
- An improved capacity to deflect stigmatization and prevent internalization of stigma.

Self-stigma and shame are deeply rooted, and it is deep work to learn how to identify and cope with these thoughts and feelings. Participants should understand the course requires an open mind and is likely to bring up many emotions. This toolkit is based on a few key understandings:

1. We need to first recognize and relate to self-stigma and shame inside of ourselves.
2. Then we can start to address self-stigma through reflective exercises which enable us to question our thinking and learn how to deal with self-stigma.
3. Addressing self-stigma allows us to be the best possible version of ourselves, free of any shame, blame, or guilt.
4. We believe every person has all the wisdom to support him- or her-self, and our role as facilitators is to hold the space for them to discover their own solutions.

What is self-stigma?

Self-stigma, also termed internal stigma, can manifest as shame, guilt and self-loathing. This can lead to reduced well-being, depression, low self-esteem, reduced self-efficacy, poor long-term coping, lower quality of life, and avoidance of social interactions even after the infectious period of TB is over.[1] Social, self, and contextual factors shape self-stigma.

Stigma occurs at different levels including the level of the self, the family, the community and at system level in the health, education, social, legal and policy levels (see Figure 1). Each level needs to be tackled with specific interventions

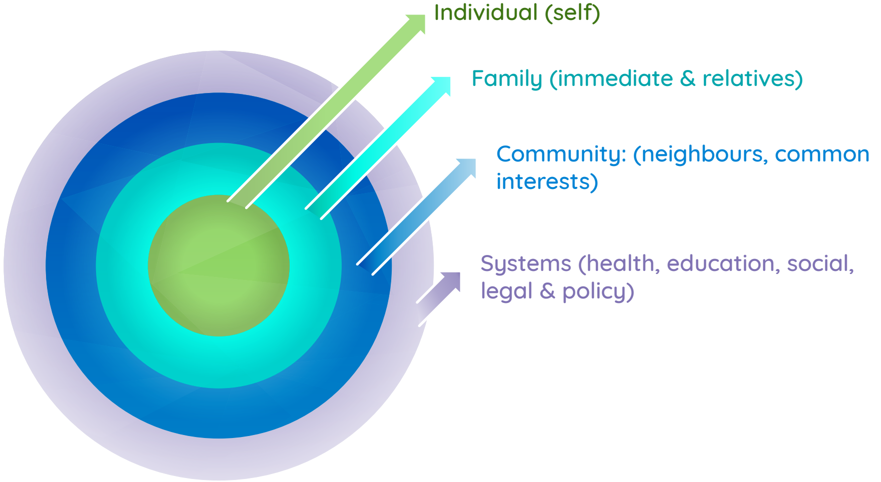

Figure 1. Stigma settings

and approaches. This toolkit however, focuses on the level of the self. Our sister toolkit "The Allies Approach" deals with stigma in the health facility setting (www.kncvtbc.org/stigma). An overview of Stigma – spaces, scope of intervention and corresponding tools can be found in Annex 1.

Exploring self-stigma

Confronting self-stigma and shame is no small thing. It requires openness and willingness for deep self-reflection. It implies letting go of the judgmental habits that harm us. The process can bring to the surface beliefs that have foundations in childhood. Working with thoughts and beliefs underpinning self-stigma often engenders resistance, as it challenges and exposes some core elements embedded in identity, deep notions of being a victim, resilience, and change. Ultimately, the result is peace and relief from the intense suffering and lack of self-agency caused by self-stigma and shame. The benefits will be felt across many areas of a person's life, above and beyond TB.

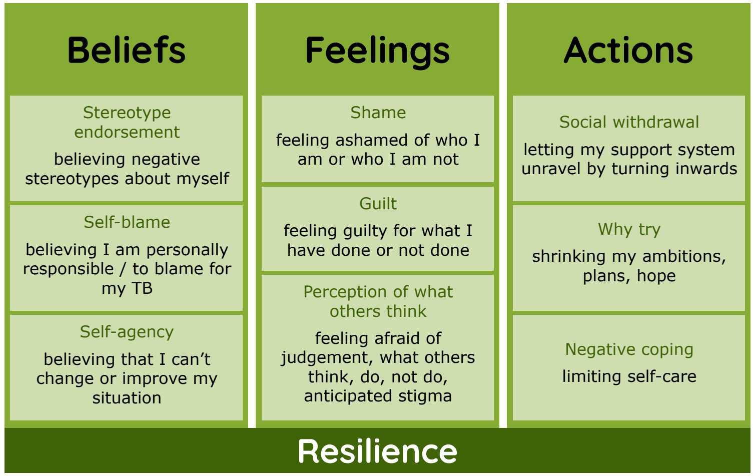

Figure 3. Beliefs, feelings and actions – self-stigma domains

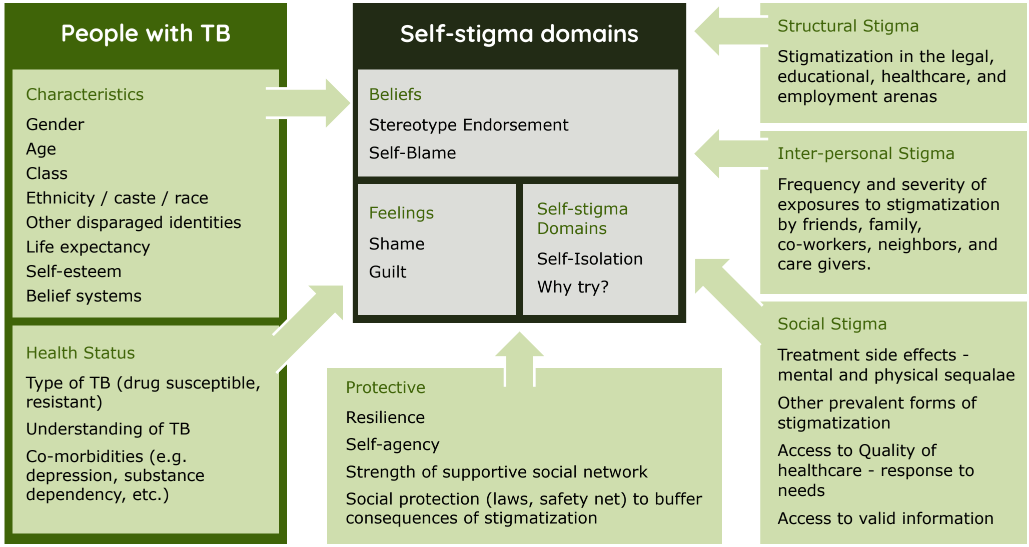

Figure 2. Conceptual framework

Theory and evidence base

This toolkit has been designed based on evidence-based research of self-stigma as it relates to various health issues such as TB, HIV, cancer, etc. Interventions must target cognitive, affective, and behavioral levels (see Figure 2).

Within each of these levels there are beliefs, feelings and actions that underpin and fuel self-stigma.

It is necessary to support people to deeply reflect on their underlying beliefs, often by connecting to the emotions that arise and then becoming aware of their action or inaction as a result. Figure 3 provides further information about the domains of self-stigma, including beliefs, feelings, and actions.

Self-stigma may arise throughout a person's journey with TB in different forms and is associated with various issues. Figure 4 shows self-stigma through the TB journey, highlighting the main areas where people need support.

This toolkit is based on the above theoretical underpinnings and provides a thorough, evidence-based and holistic approach to addressing self-stigma. In addition, it supports the participants in experiencing empowerment from the inside out and to experience the possibility of living a life that realizes their full potential.

Structure

Broad sections and modules

The toolkit is in four distinct parts in eight modules (see Figure 5). These modules are as follows:

- **Module 1:** What is self-stigma? An introduction to the concept of self-stigma.
- **Module 2:** Dealing with self-stigma and shame: Explores self-stigma and shame, enabling participants to learn how to identify and cope with the thoughts and feelings.
- **Module 3:** DR-TB: Explores the impact of DR-TB on self-stigma.
- **Module 4:** Transmission control and self-stigma: Self-stigma in the context of transmission control.
- **Module 5:** Health rights, TB, and self-stigma.
- **Module 6:** Treatment: Linkage between treatment for TB and self-stigma.
- **Module 7:** Planning for the future: TB Free! What now?
- **Module 8:** Evaluation of self-stigma and its impact.

Module selection

Please see the package outline below. Modules 1, 2, and 7, along with some specific exercises from Modules 3 through 6 are essential for understanding and dealing with self-stigma in the context of TB (see package snapshot for details of essential activities).

- **Module 1** can be run with large numbers of people (up to 100), as it is informational.
- **Modules 2 – 7** should be run with smaller groups of between 15-20 people, as they are experiential.

Note: Participant handouts are in a separate booklet.

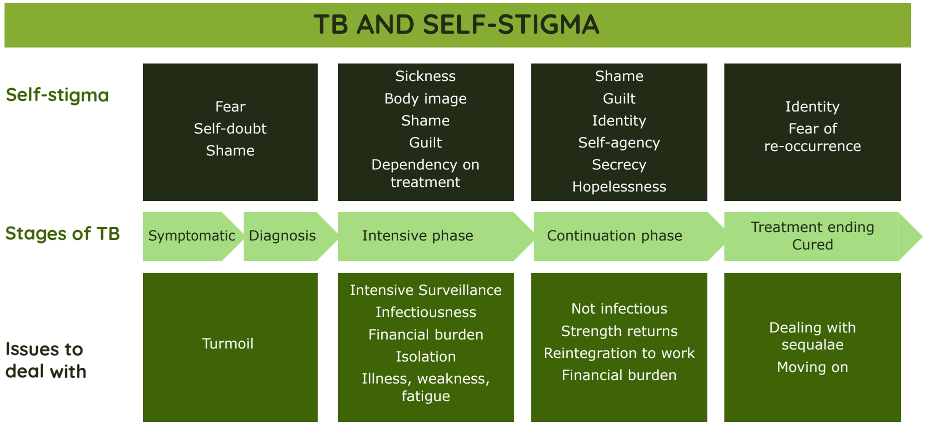

Figure 4. Self-stigma and the TB journey

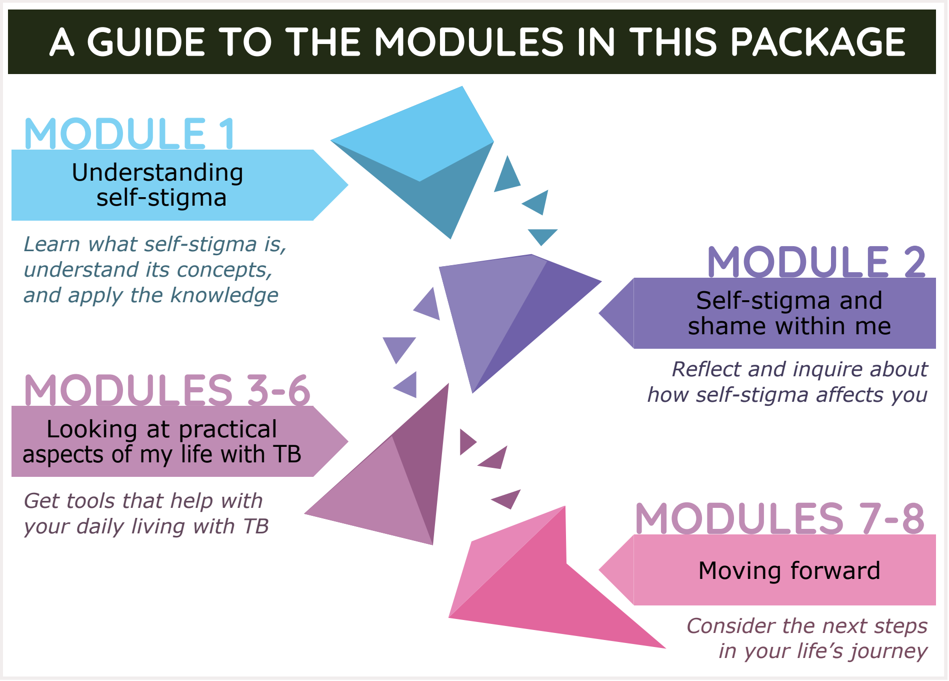

Figure 5. Toolkit structure - module overview

- **Modules 3-6** are contextual and should be used for specific circumstances as required by facilitators and the needs of participants.
- **Module 7** is important to support people 'moving on' after TB.
- **Module 8** provides information on monitoring and evaluation and serves as a guidance for implementers.

Recommended general approach

We recommend a size limit for any planned workshop (other than module 1). A cohort of 15 to 20 people should be considered the maximum number of participants to maintain a safe, confidential space. Smaller groups may be beneficial if participants experience multiple forms of stigmatization.

Delivery

This toolkit is intended to serve the needs of a wide range of people with TB. It is a first step to support people to start addressing their self-stigma.

A deeper dive alternative

For those who would like a more in-depth program on self-stigma, there is a 60-hour program in the form of a 12-session course available. This program is described in the case study at the end of Module 2 and is available from The Work for Change and in Annex 2.

Target audiences: who can benefit from this toolkit?

This intervention package is intended for people undergoing treatment for drug susceptible or drug resistant TB. It is assumed that participants have had at least one month of effective treatment and are feeling well enough to take part in the training. The continuation phase or after sputum conversion is often an appropriate time. It assumes that participants can interact in groups safely.

It is aimed specifically at:

1. People who have newly diagnosed drug-susceptible or drug-resistan TB.
2. People who have TB in conjunction with other stigmatized co-morbidities (substance dependency, HIV, diabetes mellitus, etc.)
3. People who have acquired drug-resistant TB who may experience (self-) blame or guilt linked to non-adherence.
4. People who may have had TB in the past and have relapsed.

It may also be adapted to benefit caregivers, health workers, advocates, and allies. 'The Allies Approach - Tuberculosis stigma reduction for health care institutions' is addressing tuberculosis stigma in health care workers and facilities.

How to identify participants

This complete toolkit is not for everyone. Facilitators and organizers should not assume that all people with TB would like to take this course or would even benefit from it. It is crucial that participants self-select to attend this course, as it requires openness and a willingness to explore deeper issues. Furthermore, participants need to be informed that the exploration of self-stigma and shame can evoke powerful emotions.

Screening participants to assess suitability

A way to assess individual readiness for the intervention is to invite participation in Module 1 and subsequently invite people who want to take the other modules to self-select. As previously mentioned, Module 1 can be run with large groups of people, and from that small groups can self-select for Modules 2-7.

Another option is to screen all persons starting TB treatment for self-stigma to identify people who would benefit from the intervention, and extend invitations to those with high self-stigma scores. Module 8 has information on how to screen people for the intervention.

Once participants decide to join in the intervention, we recommend that participants' baseline well-being be comprehensively measured to detect declines in self-stigma that are attributable to the intervention.

Important note on training facilitators: Participant selection is critical to successfully transferring knowledge and techniques contained in this toolkit. This is especially true for "training-of-trainers" (ToT) workshops where participants are exposed to the full toolkit with the aim of eventually using it as facilitators within their own services or peer support networks. During ToT

workshops, emphasis should be placed on the experience and acquiring the knowledge and techniques to address self-stigma, rather than conducting the training as a direct intervention against self-stigma. It will be important to select an "experienced audience" made up of individuals that are at an advanced stage, or have completed, their treatment journey, and who might already have undertaken other interventions to address self-stigma. The "experienced audience" will likely also include staff of NGOs or health institutions who are working with people who have TB.

Building commitment

Many TB patients are heads of household or have significant social and economic responsibilities. For some it is difficult to devote time to their own health. It is appropriate to acknowledge that self-care may involve trade-offs. For some it may be difficult to commit to the full intervention unless they are convinced of its socioeconomic value, so make this link clear.

Participants should be encouraged to value their own self-care and acknowledge that it may mean temporarily disappointing others or delaying caregiving. At the same time, severe socioeconomic pressures might make it impossible for participants to participate without external help. Provisions might need to be made (socio-economic/ financial support) to allow people in need to participate.

Who can implement this toolkit?

A facilitator team of approximately two to three people is the most effective way to deliver modules and activities in the toolkit. Within the team, there should be people who:

- Are able and willing to deal with their own self-stigma and shame.
- Have good technical knowledge of DS-TB and DR-TB.
- Avoid value-laden terminology in their normal speech.
- Have experience as a group facilitator
- Are skilled to deal with emotions and show vulnerability.
- Are good listeners and refrain from giving unsolicited advice.
- Can connect with people.
- Are used to working cross-culturally.
- Have good organizational skills.
- Have an ability to model making mistakes, owning them, showing self-compassion, and then moving on.

Facilitators should complete the exercises themselves *before* delivering them to groups, in particular the exercises in *Module 2*. Facilitators should ensure they are personally equipped to deal with judgments, stigma, and self-stigmatizing thoughts that may arise. Co-training should be done by pairs with diverse backgrounds and who have mutual respect for differences. Facilitators who are TB survivors and have worked through their own self-stigma can provide tangible, credible role modelling.

Facilitation

A guide on facilitation

1. Plan out which modules and exercises will be used before the session. Facilitators must consider the experience levels, cultural backgrounds, education levels, and life-stage of their participants.
2. Adapt materials to suit the participants' ways of working. For example, medications and other factors can reduce a person's ability to concentrate for long periods, or cause difficulties with hearing or eyesight. Facilitators should adapt session content to take this into account, for example by limiting the length of the taught components and interspersing them with exercises and group work. Ensuring adequate space to sit close to the facilitator, or using a microphone with amplification, can help those with hearing difficulties. Materials can be adapted to be more visual using pictures and graphics, or larger print.
3. Always set ground rules at the start of each session, and enforce if there are any lapses.
4. Welcome participants personally as they arrive to sessions or allow time at the beginning to speak to every participant individually to create a personal connection. Playing a game or using an icebreaker is a useful way for people to feel more comfortable with one another.
5. Try to create a culture of openness between facilitator(s) and the participants and between the participants themselves. This will help in sharing information and opinions.
6. Use a warm-up exercise to create active participation. For example, ask each of the participants to give their name, their age or occupation, if appropriate, and what they hope they will gain from the session.
7. Build confidentiality among the participants. They will learn that maintaining and respecting these boundaries helps to protect themselves and

others. *Confidentiality around negative coping behaviors (substance use, smoking), exposures (e.g., incarceration, homelessness) and co-morbidities (alcohol dependency, HIV) is especially important.* Participants must feel comfortable disclosing information only when they want to, and others should understand the importance of not revealing what others share.

8. Always use empowering language and terms. Do not say 'people suffering from TB, or 'TB victims'. Instead, use terms such as 'people affected by TB' or 'TB survivors'.
9. Be aware of cultural norms.
10. Be aware that group dynamics are important. Connectedness, effective engagement, and strong affinity between the facilitator and participants is important to ensure open discussions that are not dominated by a small number of people.
11. Use open-ended questions for discussion and active learning.
12. Take a creative approach when presenting – presentations should be visual and easy to understand.
13. Be aware of session pacing – ensure that there is time and space available to allow the participants to understand the content, by asking if anything requires clarification or a second look. Participants may request additional time during exercises that they strongly engage with, and this should be accommodated when possible.
14. Use creative ways to avoid session fatigue – for example, change the room layout between modules and exercises or encourage some discussions to take place whilst standing. Some of the modules' informational content may take a long time to present, therefore consider breaking up long stretches of teaching with a practical exercise.

Safe space

Safety is an important pre-condition for implementation of the self-stigma intervention package. All the modules in this package are designed to be fully participatory and will focus on ensuring participants are able to share and discuss their experiences in a safe and affirming environment. Ensure the physical space is appropriate for discussions, small-group work, and games and exercises.

A large room with visual and auditory privacy is important. Some clients may have hearing loss and background noise can impair their ability to benefit. A reasonable temperature is important to prevent excessive perspiration - especially for people with Rifampicin containing regimens, so that orange staining of clothing can be avoided. Sufficient wall and floor space for art work, mapping, posting, and presentation is necessary. Clean water and toilet facilities for participants are critical. People experiencing drug side effects may need a place to manage nausea and other physical symptoms.

The exercises can be intense, and some participants will experience sadness. Anticipate crying and have tissues available – particularly for those taking rifampicin containing regimens where tears can stain clothing.

A 'haven' approach has been shown to be a successful and effective way for supporting affected groups in the general stigma reduction context.

Safe air is vital. Precautions should be taken to keep the air fresh and the bacterial load low for the comfort and safety of everyone. The best ways to ensure safe air are holding sessions outdoors, having UVGI lights, and keeping windows open. Personal protective equipment (PPE) such as respirators are also used sometimes, but they can make it harder to interpret emotions via impairing facial communication.

Considerations when working with adults

Adults are generally goal- and relevancy-oriented and practical. We benefit from examples, stories, exercises, and discussions that allow us to reach conclusions ourselves. Linking material with personal experience is a powerful way for adults to learn and provides opportunities for synthesis, or when we can connect ideas and concepts in our own way, and then solve problems creatively. Therefore, a participatory approach is vital to ensure full engagement in and a maximum benefit from the intervention.

PACKAGE SNAPSHOT

A note on the modules and activities: This package is designed as a toolkit from which to draw activities and information on self-stigma and TB. It is not expected that the modules be run consecutively from 1-8. Rather, facilitators can select individual modules or activities for specific events and needs. The color codes represent: ***Understanding self-stigma****,* ***Self-stigma and shame within me****,* ***Practical aspects of my life with TB****,* ***Moving forward after TB****.*

Module 1 can be done with a large group, but for modules 2-7 we would recommend between 15-20 people per group.

	Module title	Description	Activities	Essential	Expected results	Time	Who will benefit?	Materials
**1**	What is self-stigma?	An introduction to the concept of self-stigma. Participants will:<br>• Understand what self-stigma is.<br>• Know how to identify the manifestations, drivers, and domains of self-stigma.<br>• Be familiar with contributing factors that lead to self-stigma.<br>• Understand the negative effects of self-stigma on an individual's life.	1.1 Understanding self-stigma and its effects	**X**	Participants will consider and understand the range of areas where self-stigma affects a person.	30m	All	Flipchart, flipchart paper for each group, markers, printed exercise outline for participants.
			1.1b Living with self-stigma		This exercise is designed to provide practical examples of lived experience and self-stigma, and broaden participants understanding of self-stigma.	60m	All	Case studies.
			1.2 Recognizing TB self-stigma and its challenges	**X**	Support participants to understand the broader challenges that are faced by people living with self-stigma.	30m	All	Printed copies of Domains of Self Stigma statements, pens.
			1.3 Recognizing self-stigma in the broader context of well-being	**X**	Participants will understand the overall effects of self-stigma on a person's well-being. This exercise emphasizes the fact that the negative internal thoughts of self-stigma create an impact on many aspects of a person's life, and these are independent of any condition.	60m	All	Printed Ryff Dimensions – things to think about, printed Ryff dimensions scale charts, colored stickers or pencils/pens (in red, yellow, or green).

Table 1. Package Overview

	Module title	Description	Activities	Essential	Expected results	Time	Who will benefit?	Materials
**2**	Self-stigma and shame within me: Reflect and inquire about how self-stigma affects you.	Explores self-stigma and shame, enabling participants to learn how to identify and cope with the thoughts and feelings.	2.1 Keeping a TB journal	X	People with TB can reflect daily on their situation and explore the challenges that they have encountered using a safe space.	30m	All, focus people with DS-TB and DR-TB	Journaling template in a stapled booklet ensuring there are at least 60 blank templates.
			2.2 "Being breathed"	X	To understand the universality, innocence, and un-intentionality of breath as it relates to contracting TB while addressing guilt and self-blame.	10m	All, focus people with DS-TB and DR-TB	None
			2.3 Universality, self –stigma, and shame	X	To get in touch with the feeling of self-stigma and shame inside of us and understand how it affects us.	60m	All, focus people with DS-TB and DR-TB	Flipcharts and post-its.
			2.4 "What I think you think about me."	X	To explore our assumptions and feelings around other people's negative judgements and to become aware of our own negative judgements of ourselves.	120m	All, focus people with DS-TB and DR-TB	Printing for participant packs:<br>• Judge-your-neighbor worksheet x 3: http://thework.com/sites/thework/downloads/worksheets/JudgeYourNeighbor_Worksheet.pdf<br>• One Belief at a Time worksheet x 4: http://thework.com/sites/thework/downloads/worksheets/onebelief_Eng.pdf<br>• The Little Book x 1: http://thework.com/sites/thework/downloads/little_book/English_LB.pdf<br>• Video to play: https://www.youtube.com/watch?v=Vqjj7nvqczo
			2.5 Who is judging whom?	X	To start to recognize how self-stigma and hypersensitivity can cloud my judgement and influence my action/non-action.	20-30m	All, focus people with DS-TB and DR-TB	Case study - Masimba
			2.6 How TB affects me	X	Strengthened personal reflection on the different ways TB affects our lives.	60m	All, focus people with DS-TB and DR-TB	Paper, markers/pens<br>soft music and speaker<br>tissues
			2.7 Do you believe everything you think?	X	To understand the beliefs people have about themselves regarding TB, and to understand the effects of such thoughts.	60m	All, focus people with DS-TB and DR-TB	Blank belief tree on a flipchart<br>Blank belief trees for participants
			2.8 Living self stigma	X	To experience the effect of living and interacting in society with and without strong TB-related self-stigma and shame in a safe environment.	60m	All, focus people with DS-TB and DR-TB	Self-stigma card deck (two sets for each to ensure enough for group)<br>Green and Blue stickers (or any two colors), markers, and chairs

	Module title	Description	Activities	Essential	Expected results	Time	Who will benefit?	Materials
			2.9 My journey - visualising TB stigma		An exercise to visually explore how TB has affected people's lives.	60m	All, focus people with DS-TB and DR-TB	Large flipchart papers, sticky tape or other adhesive to stick photos, marker pens with different colors, printed pictures.
			2.10 My right to tell	X	Participants will understand how secrecy around TB affects them and also have carefully understood the various levels of disclosure as it relates to them.	60m	All, focus people with DS-TB and DR-TB	Handout story, one for each participant. Handout disclosure, one for each participant.
			2.11 Staying in your own business: reducing stress		To know the difference between my business and the things I can change, and your business, and the things I cannot change.	60-90m	All, focus people with DS-TB and DR-TB	Three-kinds of business handout [thework.com]
			2.12 My agency, my power	X	Identify and question the self-limiting beliefs related to my TB recovery.	60m	All, focus people with DS-TB and DR-TB	One Belief at a Time worksheet x 4: http://thework.com/sites/thework/downloads/worksheets/onebelief_Eng.pdf

	Module title	Description	Activities	Essential	Expected results	Time	Who will benefit?	Materials
**3**	Drug-resistant TB (DR-TB) and self-stigma	Explores the impact of DR-TB on self-stigma. Participants will:<br>• Begin to understand self-stigma associated with DR-TB<br>• Explore strategies that can help reduce the negative impact of self-stigma in the context of DR-TB	3.1 The many faces of DR-TB	X	Understand self-stigma and shame related to DR-TB.	60m	People with DR-TB	Each case study should be available in the participant handbook.
**4**	TB transmission control	Explores self-stigma in the context of TB transmission control. Participants will:<br>• Understand self-stigma associated with limited knowledge of transmission control.<br>• Select strategies that can help reduce the negative impact of self-stigma deriving from fears of being 'a threat' or risk to others.	4.1 TB lifeline: What do we know about TB?	X	Accurate understanding around infectiousness of TB at the various stages of the disease and to identify any negative self-stigmatising beliefs that may arise.	90m	All affected by TB	TB Lifeline (either projected or draw onto a flipchart)<br>My TB Lifeline copy for each participant
			4.2 What do we know about TB and risk?		Understand the essential facts about TB.	45-60m	All affected by TB	Quiz

	Module title	Description	Activities	Essential	Expected results	Time	Who will benefit?	Materials
**5**	Health rights, TB, and self-stigma	The rights of people affected by TB. Participants will:<br>• Begin to understand the right to health in the context of self-stigma associated with MDR-TB and other forms of drug-resistant and drug-sensitive TB.<br>• Explore strategies that can help ensure better realization of and mitigate violations of right to health, and thereby reduce the negative impact of self-stigma.	5.1 Rights and the person with TB		To explore and understand rights and TB in the context of potential self-stigma.	60m	All, focus people with DS-TB and DR-TB	Handout of case studies, flip chart
			5.2 Perfectly Imperfect	**X**	A game to practice countering and deflecting stigmatization with knowledge of one's rights, self-compassion, and compassion for those who judge.	Varies	All, focus people with DS-TB and DR-TB	Card set
**6**	TB treatment and self-stigma	Linkage between treatment for TB and self-stigma. Participants will:<br>• Begin to understand self-stigma associated with taking treatment for TB.<br>• Explore strategies that can help reduce the negative impact of self-stigma in the context of TB treatment.	6.1 TB self-stigma and treatment	**X**	Understand how TB stigma and self-stigma can affect access to treatment.<br>Share ideas and experiences of self-stigma related to TB treatment.	45-60m	All, focus people with DS-TB and DR-TB	Flip chart, card and markers
			6.2 Treating my body, treating my mind	**X**	Identify self-limiting beliefs about TB treatment and understanding the effect of those beliefs on our lives.<br>Identify empowering beliefs for support during treatment.	60m	All, focus people with DS-TB and DR-TB	Flip chart

	Module title	Description	Activities	Essential	Expected results	Time	Who will benefit?	Materials
**7**	Planning for the future: TB free! What now?	What next? Planning for a life after TB. Participants will:<br>• Explore ways to plan for a life post-TB, particularly around leaving behind feelings and thoughts leading to self-stigma.<br>• Identify factors in their own lives that could impact on their social and mental health following treatment of TB	7.1 The end of my TB journey: moving on	**X**	To mark the end of TB and reflect on the journey.	60-90m	All	A number of objects, preferably from the local environment, such as rocks, stones, flowers, grass, sand. Can also include any available materials, pens, rulers, and pencils. Should have coloured markers and paper available too.
			7.2 Accompaniment: being a TB Champion		To explore ways of becoming a 'TB Champion' at a level the person is comfortable with.	60m	All	Flip charts
**8**	Evaluation	A note on the evaluation of self-stigma.	Using self-stigma scales	This module includes information measuring self-stigma, and evaluation, and is appropriate for all.				

Sample Agenda for 4-day Self-Stigma workshop

The color codes represent: ***Understanding self-stigma***, ***Self-stigma and shame within me***, ***Practical aspects of my life with TB***, and ***Moving forward after TB.*** This sample does not include module 4 (DR-TB). The format for this module would require half a day like module 5 below.

Day one	Activity	Topic
8:15-8:30 am	Registration	
8:30-9:15 am	Introductions and Expectations	
	Baseline	
9:15-10:45 am	Module 1 [exercises 1.1, 1.2 or 1.3]	What is self-stigma?
10:45-11:00 am	BREAK	
11:00-12:00 pm	Module 2 [exercises 2.1 and 2.2]	Dealing with self-stigma and shame.
12:00-1:15 pm	LUNCH	
1:15-1:45 pm	Module 2 [exercise 2.3]	
1:45-3:15 pm	Module 2 [exercise 2.4]	
3:15-3:30 pm	BREAK	
3:30-4:30 pm	Module 2 [exercise 2.6]*	
4:30 pm	Finish	

Table 2. Sample 4-day agenda

Day two	Activity	Topic
8:15-8:30 am	Icebreaker/progress review	
8:30-9:15 am	Module 2 [exercise 2.5]	Dealing with self-stigma and shame.
9:15-10:45 am	Module 2 [exercise 2.7]	
10:45-11:00 am	BREAK	
11:00-12:15 pm	Module 2 [exercise 2.8]	
12:15-1:15 pm	LUNCH	
1:15-2:30 pm	Module 2 [exercise 2.10]	
2:30-2:45 pm	BREAK	
2:45-4:00 pm	Module 2 [exercise 2.12]	
4:00-4:30 pm	REVIEW of progress modules 1 and 2	
4:30 pm	Finish	

* = *Note sequence*

Day three	Activity	Topic
8:15-8:30 am	Icebreaker/progress review	
8:30-10:00 am	Module 4 [exercise 4.1]	Transmission control and self-stigma.
10:00-10:15 am	BREAK	
11:30-12:00 pm	Module 4 [exercise 4.2]	
12:00-1:15 pm	LUNCH	
1:15-2:15 pm	Module 5 [exercise 5.1]	Rights and self-stigma.
2:15-3:15 pm	Module 5 [exercise 5.2]	
3:15-3:30 pm	BREAK	
3:30-4:15 pm	Module 6 [exercise 6.1]	Treatment and self-stigma.
4:15-4:30 pm	Review of day 3	
4:30 pm	Finish	

Day four	Activity	Topic
8:15-8:30 am	Icebreaker/progress review	
8:30-10:00 am	Module 6 [exercise 6.2]	Treatment and self-stigma.
10:00-10:15 am	BREAK	
10:45-12:15 pm	Module 7 [exercise 7.1]	Planning for the future - TB free! What now?
12:15-1:30 pm	LUNCH	
1:30-2:30 pm	Module 7 [exercise 7.2]	
2:30-2:45 pm	BREAK	
2:45-3:45 pm	Closing review and discussion	
3:45-4:30 pm	Evaluation and conclusion	
4:30 pm	Finish	

Glossary

Many of these words are used in the toolkit. Others are included here to provide further details of terminology in the stigma context.

**Agency**	The capacity and ability to make decisions for one's self.
**Anticipated Stigma**	The fear of disparagement (fear of enacted stigma) even in the absence of having the disparaged 'mark' or characteristic (condition).
**Blame**	A common rhetorical framing and a domain of stigma. This is when responsibility for a health condition is attributed to a person, or when a causal attribution is constructed, whether it is plausible, or evidence-based.
**Change Agent**	Any person within an institution that has enough social capital, respect, and leadership to catalyze new behaviors among the staff, through example, mentoring, advocacy or other means.
**Change Process**	An effective change process is a recipe for selecting, adapting, implementing, and scaling up effective practices to achieve and sustain health results.
**Courtesy Stigma**	This is a type of secondary stigma. It is the vicarious social taint experienced by those who interact with stigmatized people. Courtesy stigma may reduce the social standing of family, friends, and caretakers.
**Deconstruct**	To demystify a phenomenon by revealing its supporting structures and ideas.
**Defaulter**	A stigmatizing word used to describe a TB patient who interrupts treatment or is not compliant with treatment. No longer used. Now called a "person lost to follow up".
**Destigmatization**	The process of countering the drivers of stigma through intervention to reduce discrimination, name calling, and feelings of blame and shame.
**Dirty Work**	'Dirty work' stigma refers to employment that others regard as degrading or demeaning. Professionals serving the health or social needs of stigmatized persons may be indirectly tainted, and their roles discredited in the professional hierarchy. A type of loss of prestige experienced by certain health workers (e.g., substance dependency counselors, mental health professionals, or reproductive and sexual health care providers).
**Discrimination**	To enact stigma through illegal means or deny a person equal treatment to which they are entitled.
**Drug-resistant TB (DR-TB)**	Caused by an organism that is not cured by one or more of the drugs used to treat TB. There are many forms of DR-TB, dependent on which class of drugs the organism is resistant to.
**Drug-susceptible TB (DS-TB)**	A TB infection that responds to first-line medications.
**Enacted Stigma**	Behaviors designed to discredit or diminish - A synonym of discrimination.
**Health Rights**	The right to health is a fundamental human right, and includes access to timely, acceptable, and affordable health care.
**Infantilizing**	To treat a person as if they are immature or unable to think or act in their best interest.
**Journal**	A diary where people record their day-to-day experiences and thoughts.

**Label Avoidance**	Avoiding overt self-identification with a stigmatized group.
**Microaggressions**	Subtle forms of interpersonal stigmatization or slights and denigration.
**Pathologizing**	The practice of seeing a behavior as indicative of a disease or disorder. A way to label certain behavior as abnormal.
**Ryff Dimension**	A tool for determining levels of psychological well-being.
**Safe Space**	A place where people can share their feelings openly without judgment or fears of confidentiality breaches outside of the immediate group. Alternatively a place where the risk of disease transmission is low.
**Self-stigma**	Self-stigma, also termed internal stigma, can manifest as shame, guilt, and self-loathing. This can lead to reduced well-being, depression, low self-esteem, reduced self-efficacy, poor long-term coping, and lower quality of life. The person may avoid social interactions even after the infectious period is over. Social, self, and contextual factors shape self-stigma. Self-stigma can lead to denial of symptoms and rejection of treatment.
**Social Distancing**	When someone tries to avoid a person with TB.
**Stigma**	The relationship between an attribute and a stereotype that assigns undesirable labels, qualities, and behaviors to a person. Labeled individuals are devalued socially, leading to inequality and discrimination. For example, a TB patient is always assumed to be infectious, and therefore is labeled as dangerous. This justifies behaviors and policies that create social distance (e.g., triage, separation). Importantly, stigma is distinct from discrimination, which is an act or behavior.
**Stigmatization**	The social process by which a condition affects the lives of all of those who are impacted by it.

**Structural Stigma**	Societal-level conditions, cultural norms, and institutional practices that constrain the opportunities, resources, and well-being of stigmatized populations.
**TB Champion**	A person, often a TB survivor, who becomes a strong advocate for people affected by TB at the community or national levels.
**TB Lifeline**	A graphical way of showing where on the 'TB journey' a person may be, determined by tests, stage of treatment, and post-TB phase.
**Van Rie Scale**	Van Rie scales were developed to measure stigma and self-stigma in HIV and TB.

WHAT IS **SELF-STIGMA?**

MODULE 1

This module will guide learners through the concepts of self-stigma. It is a facilitated session with clear information, group exercises, and self-learning tools. It starts by defining self-stigma, then explores the contributing factors and outcomes to build understanding. It then gives information on self-stigma in different conditions around the world. Lastly, it contains three practical exercises for participants to apply their learning and embed knowledge.

What is self-stigma?

Timeline:

Half a day (3.5 – 4 hrs)

Required materials for this module:

Computer, projector, post-it notes or similar, printed handouts for activities, colored pens/stickers (red, yellow, and green), flip-chart/ whiteboard, and marker pens.

Introduction

Objectives of the module

After completing this module, learners will:

1. Understand what self-stigma is.
2. Know how to identify and distinguish among the drivers, domains, and manifestations of self-stigma.
3. Be familiar with contributing factors that lead to self-stigma.
4. Understand the negative effects of self-stigma on an individual's life.

Who will benefit?

This package is suitable for people living with TB, TB survivors, their families, carers, or other interested individuals. The intended facilitators are staff or volunteers from organizations who are working on self-stigma among people affected by TB.

Using the tools

Facilitators who are familiar with the package should first administer the tools. The exercises build on one another and the session should be carried out in full. Avoid going over the specified timing of the practical exercises.

"I am not what has happened to me. I am what I chose to become"

– Carl Jung

Summary of this module

This session contains six parts, which build upon each other. Firstly, facilitators will welcome participants to the session. They will then deliver a brief lecture containing information to help participants identify and understand self-stigma. Next, the participants will undertake three separate but additive activities that let them understand issues faced by people living with self-stigma. Finally, the session will be closed by a facilitator-led session wrap.

Timing and logistics

1. This session contains six parts, timed as follows. Each part is designed to lead into the next, and the session should be carried out in full:
   - Welcome participants to the session (15 min.)
   - Introduction to the session (15 min.)
   - Exercise 1: Understanding self-stigma end-to-end (30 min.)
   - Exercise 2: Recognizing self-stigma and its challenges (60 min.)
   - Exercise 3: Understanding self-stigma and well-being (60 min.)
   - Session wrap (30 min.)
2. Although people being treated for TB, or TB survivors, are experts *on their own life experiences*, they might not know the context or wider circumstances of tuberculosis and self-stigma. While this session aims to build knowledge, exploring the unique first-hand experiences of the participants can be a valuable learning experience. These discussions may take more time than expected.
3. Consider the range of education and literacy levels among learners - extra time might be needed to explain the concepts. It may be useful to have key phrases/definitions translated into a local language.

Critical issues

1. Facilitators should be prepared to deal with the emotional reactions of participants as they reflect on painful past experiences. For example, some may become visibly upset. Others may hide their emotions, and it might not be obvious that they are reacting. Facilitators should try to emphasize that the workshop is a "safe space" where discussions can be held freely.
2. This module builds basic knowledge but does not contain methods to deal with self-stigma. It should therefore always be used as part of a complete package that is aimed at informing AND empowering people to recognize and overcome TB self-stigma.
3. There is a lot of information contained in this module, so plan time to give clarifications whenever needed. Avoid session fatigue by scheduling breaks, changing the room layout between sections, and include short tasks or games to give participants a mental rest.

Additional resources

- Self-learning tool 1: Defining self-stigma – A factsheet/infographic with clear, simple language. It distills the key learning points about self-stigma. (see Annex 3)
- Self-learning tool 2: We are not alone: self-stigma around the world – A factsheet/ infographic with easily understood language. (see Annex 4)

Part I -Welcome participants to the session [15 mins.]

OBJECTIVE

Build group rapport and connect with participants to encourage open and honest discussions.

Computer, projector.

15 mins.

Facilitator-led plus discussion.

ADVANCE PREPARATION

Before the workshop day itself, it is important to assess the participant group makeup. To do this, the participants should be asked to provide the following information:

1. A short description of themselves, including locally salient characteristics, such as age, education, tribe, faith tradition, migration story, time in recovery, and working background, and where they are from (the latter may help with intercultural understanding if the group is very diverse).
2. Whether they belong to any advocacy groups.
3. What they would like to get out of this workshop.

Part II - Introduction to this session: What is self-stigma? [15 mins.]

OBJECTIVE

To deliver knowledge that allows participants to know what self-stigma is, identify its various manifestations, explore aspects of self-stigma in other conditions, and apply their knowledge of self-stigma.

Computer, projector, Module 1 Facilitator Presentation PowerPoint slides (brief facilitator notes accompany each slide).

15 mins.

Facilitator briefing participants.

ADVANCE PREPARATION

Facilitators should familiarize themselves with the contents of the Module 1 Facilitator Presentation beforehand and ensure that there are no unfamiliar concepts.

What is this session about?

This session will show learners what is meant by "self-stigma". They will explore the definitions of self-stigma, how it manifests, what it does, and how it is harmful. Exercises include ways to better understand the effects of self-stigma on a person's well-being. Central themes of this session are:

- **Define:** Participants will learn what self-stigma is.
- **Understand:** Participants will learn what the contributing factors to self-stigma are. They will also learn what self-stigma does to people who experience it.
- **Expand:** Participants will learn the scale of self-stigma, and the range of people around the world who are vulnerable to it. There will also be a brief discussion on the different manifestations of self-stigma during the TB treatment process.
- **Apply:** Participants will learn how to measure self-stigma using simple questionnaires, to help understand the issues that contribute to self-stigma.

What will the group do during this session?

The participants will explore the definitions, contexts, and outcomes of self-stigma, as guided by the facilitator. They will then undertake practical exercises in pairs or groups to help embed this knowledge.

FACILITATOR'S STEP-BY-STEP INSTRUCTIONS

1. Guide participants through the Module 1 Facilitator Presentation, while ensuring that they understand the points that have been made on each slide.
2. **At slide 3, BEFORE looking at the definition:**
    - Ask the participants to close their eyes.
    - Ask them if any of them have ever thought "I'm not good enough".
    - Ask them to raise their hands if they have had that thought.
    - Ask them to open their eyes and look around the room. Most will have their hand raised.
    - Let them know that they are ALL experts on self-stigma, whether they knew it or not.
3. Spend a few minutes on slides 3 and 4. The description of self-stigma is long, but useful.
4. Use slide 5 to ensure that participants understand the ways that self-stigma fits into the overall ecological model of stigma. External stigmas often have parallels with self-stigma, for example where stigma might cause healthcare workers to deny care to an individual with TB, the individual might not engage with care services due to self-stigma.
5. Make sure that participants understand that individuals can experience self-stigma differently. They might experience stigma as blame OR guilt OR shame – or all those things. All are manifestations of self-stigma. (Slide 6-8)
6. Emphasize the fact that self-stigma can be the result of many circumstances (slide 9) and is affected by many interacting factors.
7. Emphasize the point that self-stigma affects a person's LIFE (slide 10). It is never just a 'negative feeling'. Self-stigma can lead to isolation, depres-

sion, lack of self-care, reduced treatment adherence, etc.

8. Ensure that participants understand that self-stigma can be experienced by ANYBODY (slide 11). Note the examples given in slides 12 and 13. Slide 12 shows the different dimensions of self-stigma, and slide 13 gives an overview of self-stigma in different conditions around the world.
9. Emphasize that self-stigma CAN be overcome. Different people do it in different ways. Slide 14 gives a quick overview of what others are doing to overcome stigma. Reassure participants that tools and activities to overcome self-stigma are coming up in later modules.
10. Move on to the specific timeline of self-stigma as it might relate to TB and TB treatment. Show that different manifestations of self-stigma might affect people with TB at various points during their TB journey (slide 15). It may be useful to ask if participants know of other people who experienced self-stigma. Videos can be used.
11. Then move on to Exercise 1.1 (Slide 16)
12. Optional: Before moving on from this exercise, consider also doing exercise 1.1b
13. After Exercise 1.1 is done, consider taking a short break, then move on to Exercise 1.2 (slide 17).
14. After Exercise 1.2 is completed, move on to slide 18. This slide emphasizes that self-stigma does NOT work in isolation. It impacts treatment, mental health, and more. More widely, self-stigma impacts a person's WELL-BEING. Measuring well-being can be used to find out "how well" a person is doing in life. Guide participants through slides 19-21, clarifying the concepts of well-being, and helping them understand all the ways that self-stigma can impact well-being.
15. Take a break before moving on to Exercise 1.3 (slide 22).

Before moving on from this exercise, consider the optional exercise 1.1b (see below).

Part III - Exercises

Exercise 1.1 Understanding self-stigma from end-to-end [30 mins.]

INTRODUCTION

Group exercise to explore the areas of a person's life where self-stigma can have an impact.

OBJECTIVE

To teach participants to consider the range of areas where self-stigma affects a person.

Flipchart, flipchart paper for each group, markers, and printed exercise outline for participants.

30 mins.

Group discussion and collaboration.

ADVANCE PREPARATION

Before the workshop day, familiarize yourself with the exercise.

FACILITATOR'S STEP-BY-STEP INSTRUCTIONS

1. Before starting, make sure that participants know that they do not have to discuss their own experiences. They can discuss things that happened to someone else.
2. Be prepared for emotional responses from some participants. Allow time and/or personal space for people affected.
3. Try to encourage participation from all group members. Float between groups to observe if anyone is very quiet, or if an extra-talkative person is dominating the discussions.
4. This exercise will reinforce learning from the introductory part of the session. It encourages participants to explore the manifestations of self-stigma that might appear both in themselves and others. It also encourages them to think about where self-stigma has an effect.

Time required	**30 mins.**
Group size	Participants will work in groups of three or four, with facilitator(s) floating.
Materials checklist	Flipchart, flipchart paper for each group, markers, printed exercise outline for participants

**Exercise**	
STEP 1: OUTLINE TASK	Facilitator(s) outline the task to participants: Discuss in small groups the contributors to self-stigma, and how stigma impacts a person's life. Participants can draw on their own experiences, if comfortable, or they can discuss things that have happened to other people.
STEP 2: GROUP EXPLORATION	Each group will discuss and explore the following questions, and write down their answers:<br>1. What are some situations, actions, beliefs, or contexts that might influence self-stigma?<br>2. What areas of a person's life can be impacted by self-stigma?<br>3. How might stigma affect different types of people differently?
STEP 3: DISCUSSION	The facilitator will seek input from each group in turn, writing down some of the key answers on the flipchart, divided into two sections:<br>• Contributors to self-stigma: "What leads to self-stigma?"<br>• Areas in a person's life where self-stigma has an impact: "Where is self-stigma?"<br>Example discussion may include how many of these factors do participants think are limited to people with TB? This expands on the point that self-stigma is not limited by condition or geography

Exercise 1.1b Living with self-stigma [15-30 mins.]

INTRODUCTION

This is a short, optional exercise to show some examples of self-stigma using a series of case studies based on the 'Chitter Chatter' project facilitated by the GCTA Community.

OBJECTIVE

To provide specific examples of the impact of stigma on people living with TB.

Copies of the case studies (or access to the participant handbook and flipchart for discussion feedback.

15-30 mins.

Group discussion and collaboration

ADVANCE PREPARATION

Before the workshop day, familiarize yourself with the exercise, and print out the case studies for dissemination if the participant handbook is not being used.

FACILITATOR'S STEP-BY-STEP INSTRUCTIONS

1. Share the case studies with the group. Ask participants to select one to read through.
2. Ask the participants to consider three core questions in relation to the case studies they have selected:
    - In your view, what are the differences between self-stigma, social stigma, and discrimination?
    - In your view, what are the biggest issues relating to self-stigma in the case study?
    - Are there any possible solutions that could be useful for your own experience of self-stigma?
3. After an agreed time (15 mins. is appropriate), ask participants to share their responses. Use this to discuss self-stigma.

Exercise 1.2 Recognizing TB self-stigma and its challenges [60 mins.]

INTRODUCTION

This is a paired or group exercise in measuring self-stigma, followed by a discussion of management issues that may arise. Build upon Exercise 1.1 by helping participants understand the broader challenges that are faced by people living with self-stigma.

The self-stigma domain items were culled from validated self-stigma scales for other stigmatized conditions.[1–4] This is not strictly a measurement exercise to assess levels of self-stigma among the participants. By asking them to rate each statement, they will better understand and absorb the ideas as compared to telling them what the domains are, or giving them a list to read through.

OBJECTIVES

1. To help participants to reflect on the underlying concepts (domains) of self-stigma; and understand their meaning.
2. To build participants' appreciation of the ways in which their own context might influence the manifestations of self-stigma.

Printed copies of the Domains of Self-Stigma statements and pens.

60 mins.

Paired work and group discussion. Participants are asked to rate their level of agreement with various statements reflecting the domains of self-stigma and resilience.

ADVANCE PREPARATION

Before delivering this exercise, familiarize yourself with the questionnaire and statements.

FACILITATOR'S STEP-BY-STEP INSTRUCTIONS

1. This exercise is not intended to teach learners to be experts in stigma measurement. Also, it is not intended to be used to assess participants' levels of self-stigma during this workshop. Instead, it builds on the previous exercise and uses a questionnaire to grow participants' understanding of the challenges faced by people living with self-stigma.
2. This exercise will also give participants a sense of the ways in which their own cultural background might influence questions and discussions about self-stigma. This will help ensure more complete discussions later on. For example, individuals not accustomed to openly discussing their emotions with relative strangers may be hesitant to participate. Reassurance that the workshop is a safe, confidential space may encourage them to open up. This will help build understanding of how culture and social norms can contribute to self-stigma, even though it may not be obvious at first.
3. Before starting, make sure that participants know that group sharing is voluntary. They can keep their own personal rating private if they do not feel comfortable sharing.
4. Space should be allocated to ensure that groups can work together without being disturbed.
5. Make sure there is enough time to examine the questions during discussion. Ask the participants if they thought any self-stigma issues did not get covered. The participants are experts in their own life experiences: they can give valuable insight into how self-stigma is experienced in their lives/families/culture. Because of this, they might give unique information that helps improve understanding of self-stigma in their group. This can be very valuable for some key populations who are difficult to access.
6. Discuss any tools or techniques that the participants themselves have used to cope with self-stigma. The questionnaire explores many negative domains, which are very important to help understand self-stigma. However, the purpose of this toolkit is to help overcome self-stigma, and participants may be able to share their own personal experiences, which may in turn be useful to other members of the group.

Practical exercise in exploring TB self-stigma

Time required	**60 mins.**
Group size	Participants will work in pairs, or groups of three, with facilitator(s) floating.
Materials checklist	Printed copies of the Domains of Self-Stigma Statements, printed copies of the van Rie scale, pens.

Exercise	
STEP 1: OUTLINE TASK	Facilitator(s) outline the task to participants (see facilitator's step-by-step instructions).
STEP 2: GROUP EXPLORATION	Working in pairs, fill in the questionnaire together.<br>Answers are coded with 1 – 7, depending on the response. 1 = strongly disagree, 2 = disagree, 3= slightly disagree, 4=not sure 5 = slightly agree, 6=agree, 7 = strongly agree. How much does the participant agree with each statement?
STEP 3: DISCUSSION	Discussion questions:<br>• What was your experience responding to the question statements?<br>• What surprised you?<br>• Where were your higher scores? Look at the questions statements in this section again. What caused these higher scores [what experience in people's lives]?<br>• How did you score on resilience? How could you improve this?

Table 3. Domains of Self-Stigma questions

Domain	Question	Strongly disagree	Disagree	Slightly disagree	Not sure	Slightly agree	Agree	Strongly agree	Summary Score
Beliefs	**Stereotype endorsement**								
	1. I think that I am less careful than others because I developed TB disease.	1	2	3	4	5	6	7	
	2. I am unclean because I developed TB disease.	1	2	3	4	5	6	7	
	3. TB disease is a sign of weakness.	1	2	3	4	5	6	7	
	4. People with TB are as responsible as anyone else.	1	2	3	4	5	6	7	
	5. People with TB are dangerous.	1	2	3	4	5	6	7	
	**Blame**								
	1. If I had taken better care of myself, I would not have developed TB disease.	1	2	3	4	5	6	7	
	2. If I had avoided certain people, I would not have TB.	1	2	3	4	5	6	7	
	3. If I had taken my ART and IPT as instructed, I would not have developed TB disease.	1	2	3	4	5	6	7	
	4. If I had gone to the doctor earlier I wouldn't have gotten so ill.	1	2	3	4	5	6	7	
	**Self-agency**								
	1. Having TB will mean I cannot further my education.	1	2	3	4	5	6	7	
	2. TB disease will make it hard to support my family.	1	2	3	4	5	6	7	
	3. Having TB will affect my changes of holding down my job.	1	2	3	4	5	6	7	
	4. I can plan for the future despite having TB.	1	2	3	4	5	6	7	

Domain	Question	Strongly disagree	Disagree	Slightly disagree	Not sure	Slightly agree	Agree	Strongly agree	Summary Score
Feelings	**Shame**								
	1. I feel confident about who I am.	1	2	3	4	5	6	7	
	2. Having TB makes me feel like I'm a bad person.	1	2	3	4	5	6	7	
	3. I am ashamed of having TB.	1	2	3	4	5	6	7	
	**Guilt**								
	1. I cannot forgive myself for exposing my loved ones to TB.	1	2	3	4	5	6	7	
	2. I feel upset that TB disease makes it hard for me to care for my family.	1	2	3	4	5	6	7	
	3. Caring for me is a financial burden on my family that is hard for me to accept.	1	2	3	4	5	6	7	
	4. My family will be discredited by my having TB disease.	1	2	3	4	5	6	7	
	5. The diagnosis was delayed because I put off going to the doctor.	1	2	3	4	5	6	7	
	**Perception of what others think (anticipated)**								
	1. Neighbors gossip about those with TB disease.	1	2	3	4	5	6	7	
	2. People assume it is my fault I have TB.	1	2	3	4	5	6	7	
	3. Health care workers think TB patients deserve TB.	1	2	3	4	5	6	7	
	4. Friends won't want someone with TB around them.	1	2	3	4	5	6	7	
Actions	**Self-isolation and social withdrawal**								
	1. I avoid interacting with others because I developed TB disease.	1	2	3	4	5	6	7	
	2. I hide myself to protect other's health.	1	2	3	4	5	6	7	
	3. I dare not to make new friends because I developed TB disease.	1	2	3	4	5	6	7	
	4. I stopped socializing to prevent rejection.	1	2	3	4	5	6	7	
	5. I avoid the health center to prevent judgement.	1	2	3	4	5	6	7	
Resilience	**Protecting against self-stigma**								
	1. Things will go back to normal once my TB treatment is done.	1	2	3	4	5	6	7	
	2. I have plans for my life after TB treatment is over.	1	2	3	4	5	6	7	
	3. I will feel relief once my strength returns.	1	2	3	4	5	6	7	
	4. When I get over TB disease, there is a lot ahead of me.	1	2	3	4	5	6	7	
	5. I can get through this.	1	2	3	4	5	6	7	

Adapted from Stevelink et al.[5]

The Van Rie Patient Perspectives Towards Tuberculosis scale[6]

The Van Rie scales were developed to measure stigma and self-stigma in HIV and TB. Four parts were created: Community Perspectives Towards HIV/AIDS; Patient Perspectives Towards HIV/AIDS; Community Perspectives Towards Tuberculosis; and Patient Perspectives Towards Tuberculosis.

The community perspectives questions can be used to measure stigma held by general communities, or specific groups such as healthcare providers. The patient perspectives questions are useful in measuring self-stigma held by individuals. Van Rie's scales were first developed in 2007-8, using input from tuberculosis patients in Thailand, combined with input from Thai and American research teams. Since their development, they have been used in many other countries and contexts. The Patient Perspectives Towards Tuberculosis scale is reproduced below (Table 4). Note that the wording of the original questions is in the third-person – i.e. "some people", rather than "I". Higher scores represent higher level of self-stigma.

Question	Strongly disagree	Disagree	Slightly disagree	Not sure	Slightly agree	Agree	Strongly agree	Summary Score
Some people who have TB feel hurt by how others react to knowing they have TB.	1	2	3	4	5	6	7	
Some people who have TB lose friends when they share with them they have TB.	1	2	3	4	5	6	7	
Some people who have TB feel alone.	1	2	3	4	5	6	7	
Some people who have TB keep their distance from others to avoid spreading TB germs.	1	2	3	4	5	6	7	
Some people who have TB are afraid to tell those outside their family that they have TB.	1	2	3	4	5	6	7	
Some people who have TB are afraid of going to TB clinics because other people may see them there.	1	2	3	4	5	6	7	
Some people who have TB are afraid to tell others that they have TB because others may think that they also have AIDS.	1	2	3	4	5	6	7	
Some people who have TB feel guilty because their family has the burden of caring for them.	1	2	3	4	5	6	7	
Some people who have TB will choose carefully who they tell about having.	1	2	3	4	5	6	7	
Some people who have TB feel guilty for getting TB because of their smoking, drinking, or other careless behaviours.	1	2	3	4	5	6	7	
Some people who have TB are worried about having AIDS.	1	2	3	4	5	6	7	
Some people who have TB are afraid to tell their family that they have TB.	1	2	3	4	5	6	7	

Table 4. Van Rie patient perspective towards tuberculosis scale

Adapted from van Rie, et al.[6]

Exercise 1.3 Recognizing self-stigma in the broader context of well-being [60 mins.]

INTRODUCTION

Paired or small group exercise in measuring well-being among people living with self-stigma.

OBJECTIVE

To support participants in understanding the overall effects of self-stigma on a person's well-being. This exercise emphasizes the fact that negative internal thoughts impact a person's life. These negative thoughts are independent of any health condition, including TB and HIV.

Printed Ryff Dimensions – things to think about, printed Ryff dimensions scale charts, colored stickers or pencils/pens in red, yellow, and green.

60 mins.

Participants work in small groups of two or three to discuss their own experiences or role-play. The size of the small groups will be determined by the availability of pens, printed materials, etc., the total number of people taking part in the workshop, and the time available. This exercise builds understanding of the wide-reaching effects of self-stigma on an individual's life. Example profiles can be found in Annex 5.

ADVANCE PREPARATION

- If discussing their own personal experiences, this exercise may cause strong emotional reactions, so facilitators need to be prepared to give adequate support.
- You can take a short break before this exercise to allow participants to mentally prepare themselves for the emotions they may feel.
- Ensure that there is adequate space for groups to spread out and discuss the exercise without interrupting each other.

FACILITATOR'S STEP-BY-STEP INSTRUCTIONS

1. Familiarize yourself with the exercise before the workshop. During the group discussion at the end of the exercise, explore what the participants think can be affected by self-stigma. Guide the participants through one or two simple examples to help build the understanding that even if a person is doing well in some areas of their life, self-stigma might cause them to struggle in other areas.
2. For instance, a person who has many positive relationships with other people and has a lot of support from friends, family, and colleagues, could still have a low level of self-worth due to self-stigma around their appearance. That might in turn affect their ability to go out and form new relationships, or to take opportunities that would expose them to others. In a more specific example, TB patients may feel guilt or shame that their families must dedicate scarce resources to care for them, resulting in poor levels of autonomy and environmental mastery despite good positive relationships with others.

Practical exercise to help understand the Ryff dimensions of psychological well-being

Time required	**60 mins.**
Group size	Participants work in small groups of two or three.
Materials checklist	Printed Ryff Dimensions – things to think about<br>Printed example profiles created by facilitators (see examples)<br>Printed Ryff dimension scale charts<br>Colored stickers or pencils/pens in red, yellow, and green

Exercise	
STEP 1: INDIVIDUAL GROUP DISCUSSION	Each group will think about and discuss the Ryff Dimensions. They will examine the three example questions in each dimension (see "Ryff Dimensions - things to think about"). Then they will think about their own lives (or the life of the person in the example profile that they picked) and how highly they would rate themselves in that dimension.
STEP 2: SCORE THE DIMENSIONS	Each individual group member will draw a line in each dimension of their Ryff dimension scale chart to show how well they think they are doing in that dimension. The score is out of 10, with 10 being the best score. For emphasis, they can also color in each section – e.g., Red = score 1-4; Yellow = 5-7; Green = 8-10.<br><br>Alternatively, if participants find it difficult to think of a score they can just shade in red for "low", yellow for "medium", and green for "high".
STEP 3: DISCUSS	Groups will discuss among themselves the reasons for their scores in each dimension.
STEP 4: GROUP REFLECTION	After everyone is finished, the group will gather to discuss their profiles and the results of the questionnaires, led by the facilitator. Key questions for members could be:<br>1. Did you notice a high score in one dimension, but a low score in another? What were the reasons for your score?<br>2. What did you learn about yourself?<br>3. What surprised you?<br>4. How does self-stigma affect you, and in what areas of your life does it affect you most?<br><br>In cases where participants used the example profiles, the discussion might include the differences between the profiles. There might be observations that some people have very high well-being in certain Ryff dimensions but very low in others.

RYFF DIMENSIONS

▶ **Self-acceptance:** Does the person like their appearance / personality / life?

▶ **Purpose in life:** Does the individual have goals or things they want to achieve in their life?

▶ **Environmental mastery:** How well does the individual cope with their circumstances and environment? Do they feel like they are in control of their life?

▶ **Positive relationships with others:** Does the individual have meaningful and good relationships with other people?

▶ **Personal growth:** Is the individual growing and improving as their life progresses?

▶ **Autonomy:** Is the individual able to live and cope independently of others?

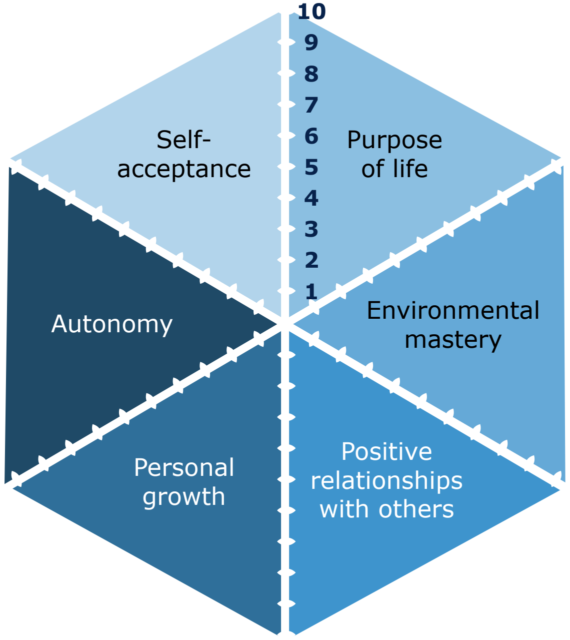

Figure 6.
Ryff dimension scale chart
Adapted from: Ryff et al, 1995.[7]

Figure 7.
Ryff Dimensions - things to think about

Ryff Dimensions – things to think about			
DIMENSION 1: AUTONOMY	Are you often influenced by people who have strong opinions?	DIMENSION 4: POSITIVE RELATIONSHIPS WITH OTHERS	Do people think you are a loving and affectionate person?
	Do you have confidence in your own opinions, even if they are different from everybody else's?		Do other people think you are generous and willing to share your time with them?
	Do you judge yourself by what you think is important, not what other people think is important?		Have you experienced many good relationships with other people?
DIMENSION 2: ENVIRONMENTAL MASTERY	Do you feel like you are in control of your life?	DIMENSION 5: PURPOSE IN LIFE	Do you make plans for your future?
	Does everyday life often make you sad?		Do you have goals in life?
	Are you good at managing the responsibilities of your daily life?		Do you feel like you still have plenty to do in life?
DIMENSION 3: PERSONAL GROWTH	Do you think it is important to have new experiences that change the way you think about yourself and the world around you?	DIMENSION 6: SELF-ACCEPTANCE	Are you pleased with how your life is?
	Has your life been a continuous process of learning and growth?		In general, do you feel confident and positive about your-self?
	Do you sometimes like to make big improvements or changes to your life?		Do you feel like many of the people you know have better lives than you?

Part IV - Session wrap [30 mins.]

OBJECTIVE

Consolidate knowledge and point towards further activities and sources.

Computer, projector, and Module 1 Facilitator Presentation PowerPoint slides (brief facilitator notes accompany each slide).

30 mins.

Facilitator-led discussion with participants.

ADVANCE PREPARATION

During the session, facilitators should note any concepts that the participants found challenging. Use this time to make sure that the participants were able to understand these points.

FACILITATOR'S STEP-BY-STEP INSTRUCTIONS

1. Revisit the points of learning from the session. Go over each point and make sure that the participants feel that they have achieved their goals.
2. Summarize some additional learning resources, including the self-learning tools and information sources that participants can use by themselves.
3. Finally, revisit the confidentiality 'agreement' with the participants. It is impossible to control what information participants disclose to others outside the workshop. However, emphasizing the importance of confidentiality and fostering empathy with fellow participants will nurture confidentiality.

Resources

In the opening section on self-stigma and concepts, and the Self-learning reference tool "What is self-stigma? Key concepts" we compiled and adapted information from articles by France et al., Morrison, Kalichman et al., and Corrigan et al.[1-4]

We also added information provided by our colleagues at KNCV Tuberculosis Foundation and Justice Edwin Cameron's address at the 17th National Congress of South African Society of Psychiatrists in Johannesburg in 2012.[8]

Information about the harmful effects of self-stigma was adapted from articles by Parker et al., Castro and Farmer, and Deacon.[9-11]

For Exercise 1.2, "Recognizing self-stigma and its challenges", we used concepts of the Domains of Self-Stigma developed by Ellen Mitchell and colleagues at KNCV based on Stevelink et al.[5], and the van Rie Patient Perspectives Towards Tuberculosis scale.[6]

For context, please see the Module 1 Facilitator Presentation.

For Exercise 1.3, "Understanding self-stigma and well-being", we used The Ryff Dimensions of psychological well-being.[7, 12]

For the self-learning reference tool, "We are not alone! Self-stigma examples around the world", we adapted information from references Brohan et al., Puhl et al., Macq et al., The People Living with HIV Stigma Index: South Africa. South African National AIDS Council., Young and NG, Oduguwa et al., and People Living with HIV [13-19]

This module also includes concepts from:
Simbayi et al.[46] and the International HIV/AIDS Alliance.[21]

DEALING WITH **SELF-STIGMA AND SHAME**

MODULE 2

This toolkit is solely focused on self-stigma. It is specifically designed to support people going through drug-susceptible or drug-resistant TB treatment. This module is part of a broader toolbox that focuses on all aspects of TB stigma and discrimination.

Self-stigma and shame are deeply rooted, and it is difficult work to identify and cope with these thoughts and feelings. Participants should understand that the course requires an open mind and is likely to bring up many emotions.

Dealing with self-stigma and shame

Timeline:

One and a half day (12 hrs)

Required materials for this module:

Computer, projector, post-it notes or similar, printed handouts for activities, coloured pens, flipchart/ whiteboard, post-its, marker pens, tissues, sticky tape, green and blue stickers, chairs, soft music and a speaker.

Introduction

Objectives of the module

This module is based on a few key understandings:

1. We need to first be able to recognise and relate to self-stigma and shame inside of ourselves.
2. Then we can start to address it through reflective exercises that support us to question our thinking, and learn how to deal with self-stigma.
3. The result of addressing self-stigma is being able to be the best version of yourself possible, free of any shame, blame or guilt.
4. We believe every person has all the wisdom to support themselves within and our role as Facilitators is to hold the space for them to discover their own solutions and realisations.

In order to support participants through Module 2, facilitators need a really thorough understanding of how self-stigma manifests during the different stages of TB. This is illustrated in the graphic below.

Who will benefit?

This module is aimed at people who are being treated for TB or those who are TB survivors. It could also be helpful for the family and/or carers who provide support and care for people affected by TB.

"The world is what you believe it to be: it changes as you change" – Byron Katie

"Self-issues are a set of concerns that positively or negatively impact self-acceptance, self-perception, self-efficacy, self-esteem and self-confidence. Self-stigma often results when self-issues interact with external causes (such as discrimination or violence in family, school, social or work settings), resulting in depression, low self-esteem, anger and self-harm, even suicidal intent."

Rahul Kumar Dwivedi, TB survivor

To support participants through Module 2, facilitators need a thorough understanding of how self-stigma manifests during the different stages of TB. This is illustrated in Figure 8.

TB and self-stigma - a map of how it manifests

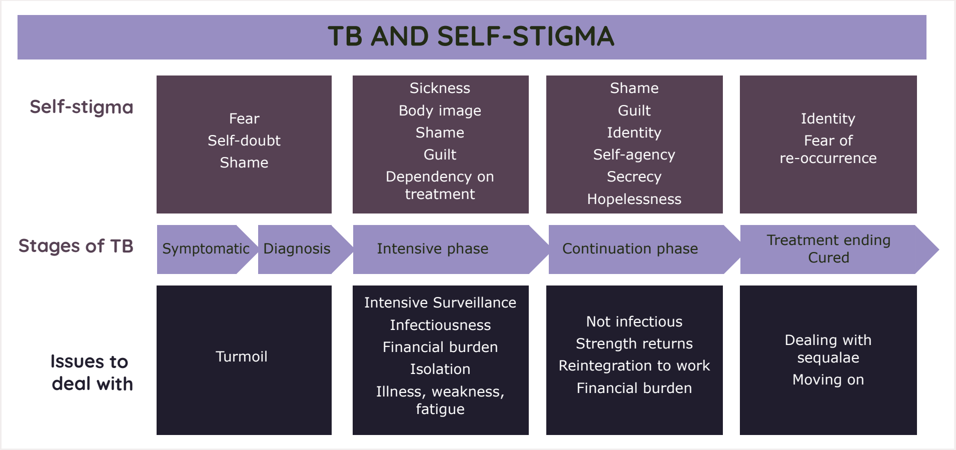

Figure 8. TB stigma along the patient care pathway

Who should deliver this module?

This module is best delivered by:

- People who are able to deal with their own self-stigma and shame.
- Facilitators who have examined their own self-stigma and shame through the work of Byron Katie, counselling, coaching, mindfulness, or any other method that has supported a deep reflective process.
- Facilitators who have had TB or been deeply affected by TB.
- Those experienced in working with groups on personal topics.
- People used to dealing with emotions and who are prepared to be vulnerable and share their own stories.

Facilitators should complete the exercises themselves before delivering them to groups.

How should this module be delivered?

Facilitators need to carefully plan which of these exercises suit the people and the time available. It is possible to select several activities if adequate time is not available, but it is preferable to perform all of the exercises. Many of these exercises are deeply reflective and require adequate time and space. This module would be best run over three to four days. Please see the suggested agendas in the introduction for more details.

Can we pick and choose which exercises to deliver?

To derive full benefit, we recommend delivering all exercises in this module. If time is limited, we recommend delivering exercise 2.1–2.8, 2.10, and 2.12. The exercises are sequential and should be completed in order. As a package, it aims to support people in dealing with guilt, shame, self-judgement, lack of information, and lack of hope. As such, it is important participants experience as many exercises as possible.

Working with feelings: Guidance for facilitators

Working with self-stigma is deep, reflective work. Facilitators should expect a lot of emotions, as memories will surface for participants, and many of them will be experiencing difficulties with self-stigma.

Feelings associated with self-stigma may include shame, loss, hopelessness, worthlessness, or rejection. Some of the exercises in this module will enable participants to get in touch with their emotions. It is necessary to first recognize the self-stigma inside all of us, allow the emotions to surface, and then we deal with self-stigma. Unlike other topics, this must be addressed from the inside out.

As facilitators, it is important to create a safe, non-threatening space where feelings, fears, and taboos can be discussed and explored openly. Setting clear ground rules and expectations around confidentiality, listening, and support are essential, and the following are some specific facilitator tips that may help:

1. Do the exercises in this module yourself first so you are aware of your own feelings and fears about each topic covered.
2. Share your feelings and experiences openly, which will encourage trust.
3. Remember to always leave enough time for participants to share their feelings and help the group to create an atmosphere where participants know they will be listened to.
4. Offer participants time-out if they need to take a break.
5. If there are any exercises you do not feel comfortable leading, find a co-trainer who can help. A certified facilitator for the work of Bryon Katie or someone trained in counselling skills would be ideal, as they will be trained in working with feelings. Other counsellors will also have a similar skillset.
6. Refrain from offering advice to participants as they discover their own realizations. The power of this module is in supporting participants to discover things for themselves.

Exercise 2.1 Keeping a TB Journal <TB/DR-TB>

OBJECTIVE

Journaling helps people with TB to reflect daily on their situation and explore the challenges that they have encountered using a personal and safe space. Modules 2 and 3 are likely to surface feelings and thoughts around stigma, both inside themselves and from others. Keeping a journal will ensure full expression and self-reflection during TB treatment and recovering from TB. Participants should journal for at least seven days, but it is recommended to continue journaling daily while on treatment.

Journaling template [Annex 6]. Facilitators should print out templates and make them into a stapled booklet ensuring there are at least 60 blank templates. Suggest also adding a nice cover to the journal with the title 'My TB Journal'.

Introduction to the journal: 30 mins. People will complete the journal daily while being treated for TB.

FACILITATOR'S STEP-BY-STEP INSTRUCTIONS

As an important part of dealing with self-stigma, we would encourage you to record your experiences using a journal throughout your treatment and journey with TB. Some participants may find journaling in this manner to be a useful structured way of exploring their experiences, and extra time may be required during this session if they engage strongly with the process.

Background: why keeping a TB journal is useful?

Research has shown that expressive writing can produce measurable changes in physical and mental health and can positively influence sleep, work efficiency, and connection with others.[20] By keeping a daily journal, participants will be able to track their positive and negative thoughts and emotions. They will gain insight into how they have changed over time, and chart progress that they have made in dealing with self-stigma. Modules 2 and 3 are likely to cause feelings and thoughts around stigma to surface both internally and externally. Keeping a journal will ensure full expression and self-reflection while undergoing TB treatment and recovering from TB.

How to do this exercise: what to share with participants

1. Set aside time to write or draw in your journal each day. Make sure that you are somewhere that you can think and write without interruptions. Use happy/sad faces to illustrate your emotions if you prefer.
2. You do not have to write everything all at once. You can add to or change the sections as the day progresses.
3. Be honest with yourself. You are the only person who will read this journal, and if you record things accurately they will be useful to reflect on in future.
4. Do not over-think things. Write what you are thinking and feeling, without worrying if it sounds strange or embarrassing.
5. Explore the way you are feeling. Can you identify the triggers that make you feel happy or sad? If so, include them in your writing.
6. Consider different perspectives other than your own. What would others think?
7. Write only for yourself.
8. When writing about things that you are grateful for or proud of, they do not all have to be big, important events. Small, personal successes are just as useful.
9. Write continuously for 15-20 minutes each day.
10. Try to do the journal every day, so that you can look back on how you felt before and use it as your journey continues.

Exercise 2.2 Being breathed <TB/DR-TB>

OBJECTIVE

To highlight the universality, innocence, and un-intentionality of breath as it relates to contracting TB while addressing guilt and self-blame.

None.

10 mins.

Meditation and reflection through questions.

FACILITATOR'S STEP-BY-STEP INSTRUCTIONS

Read the following meditation very slowly, with purposively long pauses in between sentences to allow space for people to experience their breath.

> *I invite you to get comfortable in your chair, put all papers down and feel your feet on the floor. Close your eyes and focus on your breath. Notice the quality of your breath…… Do you take long breaths, short breaths, what sound does your breath make, what does it feel like as it goes in and out? …….. [long pause here]. Get in touch with your breath, the breath you do nothing for……. Notice the ebb and flow, the quality of your breath……*
>
> *Now take on the count of three, I am going to ask you to take the biggest breath you can and hold it for as long as you can. 1 – 2 - 3*

When it seems that everyone has begun to breathe normally again you can invite people to gently open their eyes.

- Did you choose to breathe?
- Do you have a choice which kind of air you breathe?
- Can you hold your breath in if you don't want to breathe out?
- Can you decide not to breathe?
- Just notice, we are being breathed.

People with TB get sick because of breathing. This disease is not anyone's fault. No one has caused it. No one can be blamed or shamed for breathing. We must do it, there is no choice. We are all equal in that we all need to breathe.

Exercise 2.3 Universality, self-stigma, and shame <TB/DR-TB>

OBJECTIVE

To get in touch with the feeling of self-stigma and shame inside of us and start to understand how it affects us in the many aspects of our lives.

Flipcharts, post-its.

60 mins.

Group discussion and participation.

ADVANCE PREPARATION

Make a 'Wall of self-stigma and shame' and 'Effects of Self-stigma and Shame Wall'.

FACILITATOR'S STEP-BY-STEP INSTRUCTIONS

1. Self-stigma and shame live inside all of us as human beings and are big influencers of the life we live. Shameful thoughts include things such as "I'm not good enough", "I'm a failure", and "If they knew that about me…"
2. Close your eyes and answer the question 'How many of you have ever had the thought, even once in your life that you are not good enough?"
3. When you believe this thought, can you get in touch with how this makes you feel? Can you experience how it limits you, makes you feel small, separates you from those around you? Can you imagine how many things you believe you can't do when you are believing you're not good enough? This includes making a presentation, getting a job, studying, and seeking health care. Can you see how you compare yourself to others, who in your mind are better than you?
4. Invite the group to think about a specific situation [related to your TB for those who have or have ever had TB] where you had the thought 'I'm not good enough'. Be as specific as you can – where were you? What time of the day was it? Whom were you with?
5. Now pair up. Turn to your partner and share your situation/story with them and tell them how it made you feel. Take three minutes each. The facilitator will let pairs know when it's time to switch.
6. Write down all the emotions you felt associated with the situation when you felt you were not good enough. Write down one emotion per post-it.
7. Invite participants to come forward and place their emotions on the Wall of 'Self-stigma and shame' (flipchart).
8. Now invite participants to write down on the post-its all of the things they think they cannot do or won't have when they believe that they are not good enough.
9. Invite participants to come forward and place their emotions on the 'Effects of self-stigma and shame Wall' (flipchart).
10. Discuss the results with the group, using prompts such as:
    - What surprised you?
    - Are you surprised by the power of self-stigma?
    - What similarities are you seeing between people's experiences of self-stigma. Prompt them to notice how prevalent self-stigma is regardless of TB. It doesn't matter if you are living with TB or HIV, have had a difficult childhood, or you are going through a relationship break-up, self-stigma and shame may be there.
11. Self-stigma lives inside all of us. Can we recognize it and do something about it or will we allow it to have its hold on us and dictate what we can and can't do and who we are.

Take away tip:

The next time you have the thought 'I'm not good enough', consider carefully if it is true. Then find at least one specific example where you are good enough.

Exercise 2.4 What I think you think about me <TB/DR-TB>

OBJECTIVE

To explore our assumptions and feelings around other people's negative judgements of us and to become aware of our own negative judgements of ourselves.

Flipcharts, Post-its, and printing for participant packs:

3 x Judge-your-neighbor worksheet: http://thework.com/sites/thework/downloads/worksheets/JudgeYourNeighbor_Worksheet.pdf

4 x One Belief at a Time worksheet: http://thework.com/sites/thework/downloads/worksheets/onebelief_Eng.pdf

1 x The Little Book: http://thework.com/sites/thework/downloads/little_book/English_LB.pdf

Video to play: https://www.youtube.com/watch?v=Vqjj7nvqczo

120 mins.

Group discussion, The Work of Byron Katie: Inquiry-based stress reduction and reflection.

ADVANCE PREPARATION

Make a flip chart on the wall with the title 'Wall of Stigma'. Put up on a second flipchart:

Turnarounds:

a. He/she didn't judge me (opposite).
b. I judged him/her (other).
c. I judged me (self).

FACILITATOR'S STEP-BY-STEP INSTRUCTIONS

1. Invite participants to think about what society thinks of people with TB and people with drug-resistant TB.
2. Reflect on times when you yourself have been judged or times where you have heard about others being judged.
3. Invite participants to make a list or all the negative beliefs and judgements they think society has about people living with TB, and give some examples to start.
   - People living with TB are dirty.
   - People living with TB are dangerous.
   - People living with TB are to blame for their illness.

   All participants should use the prompt 'People with TB/DR-TB are…….'
4. Each belief should be on a separate post-it.
5. Invite all participants to put their beliefs on the Wall of Stigma, with a (DR) in brackets for those judgements specifically related to DR-TB.
6. Then start a discussion based on the following questions:
   - Where do these thoughts come from?
   - Why do people have these thoughts?
   - Who is society? (people will say these thoughts come from society)
   - Are we part of the same society? We can identify these thoughts because we have been socialized in the same way.
7. Invite each participant to find a specific situation in their lives where someone judged them because of their TB. Allow adequate time for everyone to find a specific situation. Invite participants to really allow themselves to feel what it felt like to be judged. Ask participants who have experience of this to share what it felt like.
8. Invite participants to fill in a judge-your-neighbor worksheet based on that situation where they felt judged. Play the following video so that the group can together fill in a Judge Your Neighbor Worksheet: https://www.youtube.com/watch?v=Vqjj7nvqczo.

9. Pause the video after #1 to make sure everyone fills it in with the same statement - **I am [emotion] at [name] because he/she judged me**.
10. When everyone is finished, invite all participants to take their statement for #1 - for example: "I am angry at Sharon because **she judged me**, and transfer just the statement **'she judged me'** onto a One Belief at a Time worksheet.
11. Then invite all participants to take 20 minutes to answer the questions on the worksheet, writing as much as they can. This is a meditative, reflective exercise, so invite them to take their time and really consider their responses.
12. When they get to the last part of the worksheet let them know that the turnarounds they will look for are visible on the flipchart:
    - He/she didn't judge me (opposite).
    - I judged him/her (other).
    - I judged me (self).
13. Bring the group back together and invite people to share what they learned.
14. Then invite people to share what they learned about themselves. People may discover that when they thought someone was judging them, they may have in fact judged that person or judged themselves.
15. Wrap up the session by sharing that this is a process of considering that what we initially think or believe may not always be true.

Homework

Take one statement from #2, #3, and #4 off the Judge Your Neighbor Worksheet and complete a One Belief at a Time for each one. Read 'The Little Book'.

FACILITATOR'S STEP-BY-STEP INSTRUCTIONS

It is recommended that the Facilitator complete the exercise for themselves before holding a group. They should find a time they were judged for something in their life and complete the exercise steps 8 through 17.

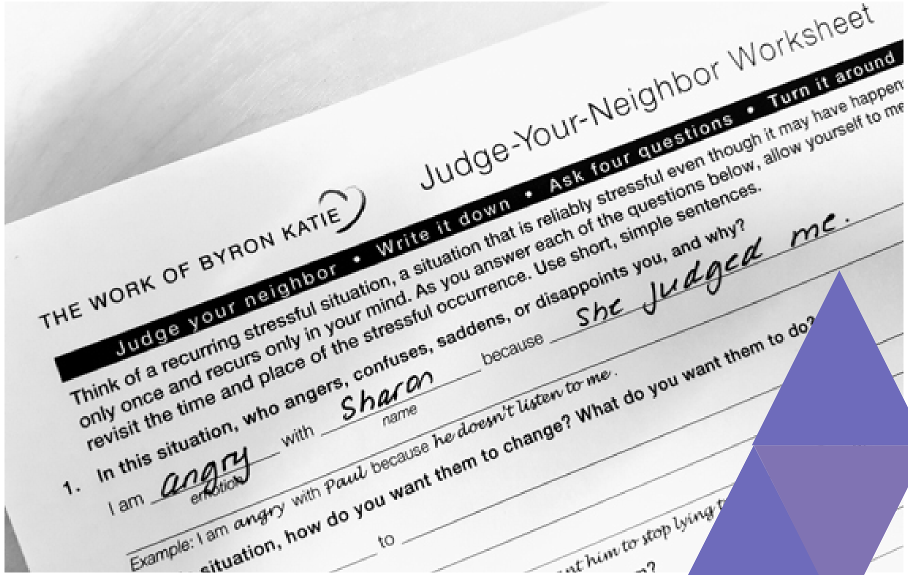

SUMMARY

- What we think is true is often not true. Any time we feel stress, it is worth questioning what we are believing.
- We all make assumptions about people, and sometimes they can be wrong

Take away tip:

Anytime you feel judged by someone for having TB, you should stop and ask yourself if they are really judging you? Ask yourself in that moment are you judging you?

Adapted from: We are the change: Dealing with HIV-related self-stigma. Facilitators Guide Using the Work of Byron Katie: Inquiry-based stress reduction: www.theworkforchange.org - www.thework.com

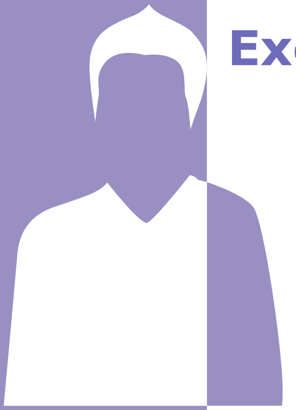

Exercise 2.5 Who is judging whom? <TB/DR-TB>

OBJECTIVE

To recognize how self-stigma and hypersensitivity can cloud my judgement and influence my actions.

None.

20-30 mins.

Theatre.

ADVANCE PREPARATION

Prepare paper script/story.

FACILITATOR'S STEP-BY-STEP INSTRUCTIONS

1. Ask a participant to read aloud the story below. One of the Facilitators can play the role of Masimba. Stop and listen in to some of Masimba's thoughts (say them aloud).

2. This exercise can be an audiovisual way of communicating the wide reaching effects of self-stigma on the individual. It can also be useful in delivering these concepts to the general audience, since no background or special knowledge is needed to understand it.

Masimba had been feeling unwell for quite a few weeks and has a bad cough. Three weeks ago Masimba tested positive for TB. At first, he seemed okay and felt calm, but for the last few days he has been feeling that everyone is watching and talking about him.

He gets the bus to work and overhears two women talking about someone who is sick and has lost weight. He looks at his own body and is sure that he is losing weight and looking thin. He wonders if they are talking about him.

At work he notices a new poster about getting tested for TB. Masimba has not told anyone at work about testing positive for TB, but he immediately thinks someone has guessed and put the poster up as a way of telling others to be careful of him.

At lunch, he goes to join a table of co-workers but there is no space at the table. He immediately thinks it is because they don't want him near them. Feeling rejected, he goes and sits on his own to eat his lunch.

His boss asks him how he is feeling, and he thinks she is asking him about having TB. He wonders if he looks sick. He starts to sweat, and gets a headache. He thinks he should have stayed at home today.

His boss then tells him, he will be working on a new project for the next four weeks. Although this is a fantastic opportunity that he has been looking forward to, now he thinks she is trying to get him out of her department.

The final straw is when a co-worker brings him a cup of tea in a brand-new mug. Now he is sure that everyone is talking about him having TB. He feels miserable, isolated, alone, misunderstood, and angry.

*If working with MDR-TB patients, adapt this case study to talk about Masimba having been diagnosed with MDR-TB.

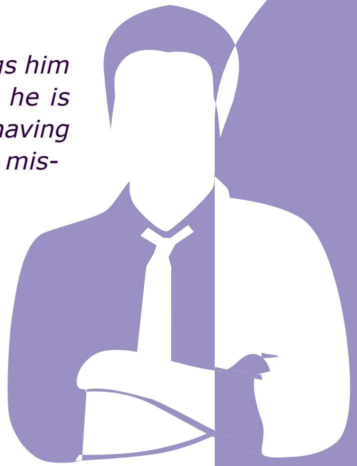

3. Invite participants to discuss:
   - What is happening in the story?
   - Has anyone had a similar experience, with TB or anything else in their life?
   - How do you show up to a given situation, such as work, when you feel people are judging you?
   - How can we support ourselves to not make assumptions?
   - How can we give ourselves a reality check? Be specific.

Take away tip:

- When you feel judged, check your assumptions

- Learn to recognize that you can create your own stories about what other people think of you.

- Understand you can start to become paranoid. When people react, could they be normal reactions?

- Laugh at yourself, and keep in mind everything is not always so serious.

- Stigma and discrimination happen. Have approaches to deal with them.

- Self-stigma also happens and starts inside of us. The difference with self-stigma is that we can do something about it from the inside out.

Adapted from: International HIV/AIDS Alliance Understanding and challenging TB stigma. Toolkit for action: Coping with Stigma[21]

Exercise 2.6 How TB affects me? <TB/DR-TB>

OBJECTIVE

It can be tempting to judge ourselves based on past behaviors and choices. This module will encourage participants to deeply reflect on how TB has affected their lives since diagnosis. By sharing their stories and then writing a letter, participants can find greater peace and acceptance of TB as well as kindness for themselves on their TB journey.

Paper, markers/pens, tissues, soft music and a speaker.

60 mins.

Reflection, pairing, and a letter of amends.

ADVANCE PREPARATION

Be prepared for emotions to arise. This means allowing emotions to happen and not trying to comfort the person. Give space and time for the emotions to run their course. Prepare a "letter of amends" template on flipchart to be shown at the appropriate time in the session.

FACILITATOR'S STEP-BY-STEP INSTRUCTIONS

1. Start by explaining that this exercise is a personal reflection and emotions will naturally surface.
2. Invite all participants to find a space in the room and think about how their life has been affected by TB. Invite them to ask: How has your life changed emotionally because of you or someone close to you getting TB? Think about what happened. How did it feel? Did you feel shame in relation to TB?
3. Now invite participants to pair up and to share their stories with each other. One person should share their story while the other person listens carefully. There should be no crosstalk. Remind the person(s) listening to make eye contact with the person sharing and not to comment beyond "thank you". After five minutes, facilitators call switch and the other person shares their story.
4. Invite volunteers to share their story with the big group, stressing that no-one has to share their story. After two to three stories have been heard ask the group if they notice any similarities and how shame affects people.
5. Then invite participants to get a piece of paper and a pen and write a letter of amends to themselves (playing soft music if possible):

> *Dear Me,*
>
> *Identify three things you did to hurt yourself and apologize sincerely for those three things. Share three things that you have given yourself and that you are grateful for. Thank yourself.*

   Sign it 'I Love you' if it feels right, along with your name.

6. Invite volunteers to read their letter.
7. Invite three to four people to read their letters and then invite people to turn to their partner and take turns reading their letters. Again, allow "thank you" to be enough for the listener. When both pairs complete the exercise, invite them to be silent..

Informed by

- *International HIV/AIDS Alliance Understanding and challenging TB stigma Toolkit for action. [22]*
- *We are the change: Dealing with HIV-related self-stigma. Facilitators' Guide Using The Work of Byron Katie: Inquiry-based stress reduction: www.theworkforchange.org www.thework.com*

Exercise 2.7 Do you believe everything you think? "I have TB and that means that…." <TB/DR-TB>

OBJECTIVE

To understand the beliefs people have about what having TB means to them, and to examine the effects of these thoughts.

This exercise will help people begin to understand the powerful effect believing negative thoughts about TB has on their lives and aid them in understanding what their life would be like without these thoughts. Participants can then explore the opposite of their original beliefs. We all have many negative self-judgements and we can change them if we see the power our thinking has on our lives and break these patterns. Other's judgements of us only have power if we believe them. We have no control over other's thoughts, but we can choose whether we believe them or not or if we apply meaning to those judgements.

Blank belief tree on a flipchart, blank belief trees for participants, pens and marker pens.

120 mins.

Problem tree

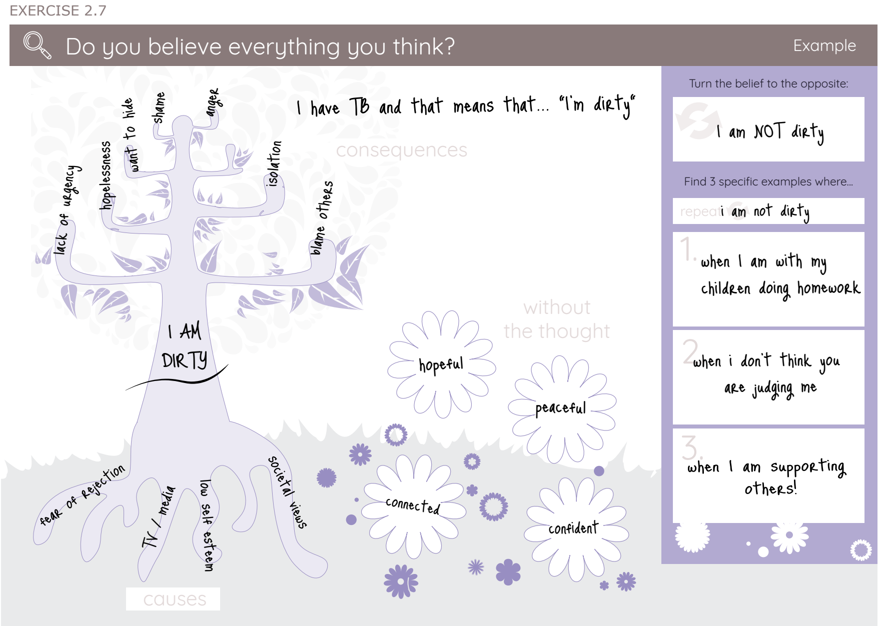

Figure 9. Example of problem tree

FACILITATOR'S STEP-BY-STEP INSTRUCTIONS

1. Invite participants to think about what society thinks of people with TB. Participants can view the Wall of Stigma from Exercise 2.5 - what I think you think about me.
2. Reflect on the times having TB has really affected you.
3. Invite participants to make a list:

   I have developed TB disease and that means …

4. When everyone has their list, ask them to circle the top two items that cause them the most stress and invite them to share these with the group.
5. Now take one belief that comes out in more than one person's list and bring it as a group to the 'Belief Tree'.
6. Put the belief (for example, 'This is my fault') into the space in the middle of the tree.
7. Then ask participants to name the causes of this belief (such as low self-esteem, cultural norms, the media, etc.) Write them each as roots of the tree.
8. Then ask participants to name the consequences of believing the thought 'I am dirty 'The effects may include isolation, blaming others, shame, a lack of self-agency, not seeking support, etc. Write them as branches of the tree.
9. Now ask them to consider who they would be without the thought. ('If you wouldn't have that thought, even just for a moment, who would you be?' i.e., more peaceful, confident, hopeful). Write down each effect in the flowers of the new garden.

10. Now ask participants to turn the original upside down, exploring the opposite of this thought, and to find three genuine examples of where this is true. For example, "I am not dirty when I am supporting others."
11. Invite participants to do this exercise alone as a means of self-reflection. They should complete two Belief trees using the two thoughts they circled. Extra time may be needed to ensure that participants fully understand how to turn a belief around, and the actions or situations that accompany the turnaround.
12. After the exercise is complete, invite participants to share what they have learned.

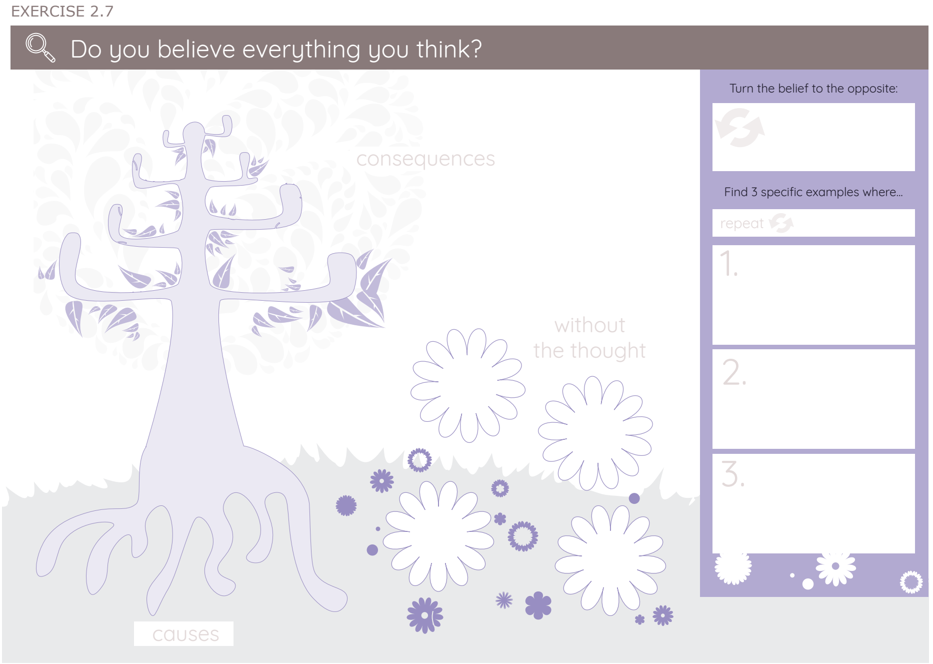

Figure 10. Blank problem tree

Examples to share:

I have TB and that means that:

- I am dirty.
- I am unlovable.
- I did not take care of myself.
- This is my fault.
- My family will be discriminated against.
- I will be a burden upon my family.

Inspired by

- *We are the change: Dealing with HIV-related self-stigma. Facilitators Guide Using The Work of Byron Katie: Inquiry-based stress reduction: – www.theworkforchange.org*
- *Gender or sex: who cares? Skills-building resource pack on gender and reproductive health for adolescents and youth workers. de Bruyn, Maria and France, Nadine, 2001. Chapel Hill: Ipas & Health & Development Networks (HDN) [23]*

Exercise 2.8 Living self-stigma <TB/DR-TB>

OBJECTIVE

To experience the effect of living in and interacting with society with and without strong TB-related self-stigma and shame in a safe environment.

Two decks of self-stigma cards, green and blue stickers (or any two colors)[Annex 7], marker pens, chairs.

60 mins.

Simulation experience.

ADVANCE PREPARATION

Review and adapt the exercise as necessary.

Note: This exercise may create discomfort among participants because some will be asked to stigmatize others, and some will be stigmatized. For this reason, this exercise should never be used with participants who are new to the concept of self-stigma, or who are at the early stages of their treatment journey. The other Module 2 exercises that precede this one are considered to be preparatory exercises that will help participants in dealing with emotional reactions around self-stigma. This exercise should therefore only carried out with experienced groups, who have also completed the preceding exercises.

Additionally, facilitators should ensure that participants are fully briefed that this is a role-playing or acting exercise. After the exercise is completed, the group should discuss negative experiences that arose, and facilitators should make clear that none of the stigmatizing interactions should be taken as personal attacks. If possible, time should also be given for participants to talk with their discussion partners, so that reconciliation or apologies can take place.

FACILITATOR'S STEP-BY-STEP INSTRUCTIONS

1. Divide people into two groups, A and B.
2. Ask all the people in group B to leave the room.
3. Distribute all the self-stigma cards to those in group A.
4. Give all people in group A a Green sticker.
5. Invite those in group A to rejoin group B participants. Their job is to really take on the role of someone experiencing the self-stigma attribute they have on their card. For example, someone with a shame card would respond to a conversation with group B members as someone who deeply feels shame about themselves. They cannot however, explain to group B what they are doing. During the interactions, they can speak with any member of group B, identified by a blue sticker.
6. Give group B the blue stickers and the self-worth cards.
7. Members of group B need to take on the role of someone experiencing the self-worth attribute they have on the card. For example, 'self-worth'. In the social interactions they have with group A members, they should be as curious and ask as many questions as possible about the lives of group A members, particularly related to TB. They should also share as much as they can about their own lives, as a person who has high levels of self-worth. They cannot explain to members of group A what they are doing. They can interact with anyone in group A, as identified by the colored stickers.
8. After 5-10 minutes tell participants to wrap-up their interactions.
9. While still standing, invite participants to share their attribute and their experience of the interaction:
   - How did your attribute affect your participation?
   - How did it affect your listening?
   - How did it make you feel about yourself?
   - What else did you notice?
10. Collect all the cards.
11. Invite group A to go outside the room.

12. Distribute the self-stigma cards to group B and give them the same instructions for interacting with group A, with the additional information that during the next phase they will be stigmatized by group A. If they feel uncomfortable with this, they should be given the option to withdraw from participation and act as observers.
13. Group A will now be given the self-worth cards, and they now will be asked to overtly stigmatize any members of group B that they interact with.
14. After 5-10 minutes (depending on the group), tell participants to end their interactions.
15. While still standing, invite participants to share their attribute and their experience of the interaction:
    - How did your attribute affect your participation?
    - How did it affect your listening?
    - How did it make you feel about yourself?
    - What else did you notice?
    - How did it feel to be stigmatized?
    - How did it feel to stigmatize?

Exercise 2.9 My journey - visualizing TB and stigma <TB/DR-TB>

INTRODUCTION

It can be difficult to keep track of all the thoughts one has about stigma, so this exercise helps participants structure their experiences for others. This lets participants understand that their experiences may be like that of others, and to learn the ways in which TB self-stigma has affected other people.

This information will help them to quickly recognize self-stigma as they encounter it in future. The aim is to help participants gain a positive attitude that lets them recognize and understand TB self-stigma, and to help them resist its negative influence. This is related to the inoculation theory, whereby a person can become resistant to 'attacks' if they are exposed to a weakened form of a particular negative attitude.[24]

OBJECTIVE

This exercise aims to help people discuss issues relating to TB, stigma, and self-stigma. It uses visual tools that help them to describe their experiences, feelings, and ideas. By doing this exercise, participants can share their unique story with others. They will gain collective knowledge around self-stigma, allowing them to recognize it more easily and aid the development of coping mechanisms. This will help reduce self-stigma. It will also help protect the participants against manifestations of self-stigma that they might not yet have encountered.

Large flipchart papers, sticky tape or other adhesive to stick photos, marker pens with different colors, printed pictures. Additional pictures will be available here: https://drive.google.com/open?id=1kGKQDm5VSN8PslOL2H1lmSCY5wX_O6b0

60 mins.

Discussion and visual narratives.

FACILITATOR'S STEP-BY-STEP INSTRUCTIONS

Sharing knowledge, experiences, and coping strategies will help reduce self-stigma among participants. It will also help prepare and protect others who are in earlier stages of their treatment journey, so they can recognize and understand self-stigma.

1. Participants should work together in groups of two or three to foster discussion and sharing of ideas. Each participant should create their own individual collection of pictures and images.
2. Facilitators should ensure that enough printed materials are available for everyone.
3. Each participant will build up a pictorial representation of their journey with TB.
    - First, participants will draw an outline representing their body. This can be a simple stick figure, or they can add more detail however they wish.
    - They will then draw or add arrows pointing to parts of their body that they feel have been changed due to TB and stigma.
    - Next, they will place photos representing the concepts of what TB and stigma mean to them, near the parts of their body where they feel there is an attachment.
    - Participants can place the photos in areas that represent specific concepts and meanings. For example, photos may be placed near the head to represent ideas or plans, near the heart to represent things that are very important, or near the feet to represent things that are now behind them in their journey.
    - Participants can also write "in my past", "in my present", or "in my future" next to the pictures to guide their thoughts.

4. Some example images and concepts could include:

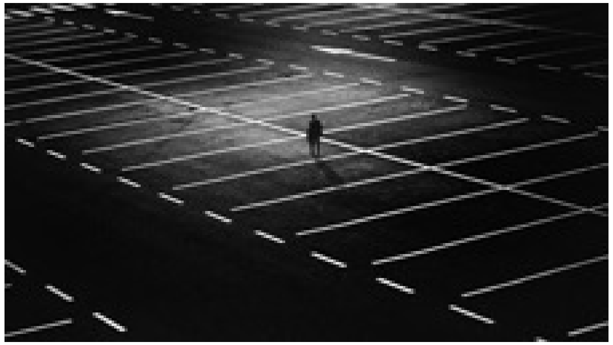

(A picture placed near the feet to represent being behind the participant on their journey.)

IN MY PAST: **"At first I felt isolated and lonely because of my TB."**

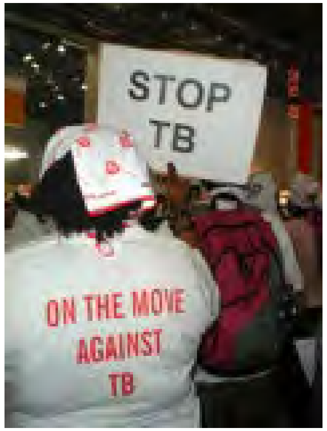

(A picture placed near the heart because of the closeness of the participant's family.)

IN MY PRESENT: **"I get a lot of support from my family who help me cope with TB treatment."**

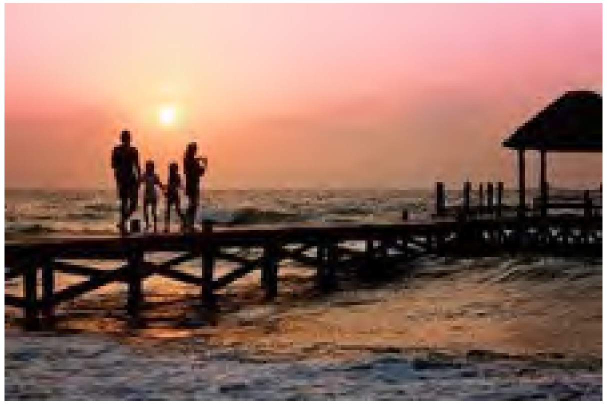

(A picture placed near the head as an idea and hope for the future.)

IN MY FUTURE: **"I am going to become an activist to help others and fight stigma."**

5. After all participants have completed their composition of images, the group should gather for a discussion. Facilitators can ask if anyone would like to volunteer to show their journey. If time allows, and participants are willing, everyone can have a chance to share.
6. The person showing their journey will guide the other members of the group through what drawings and images represent.
7. Key talking points include: What do the pictures symbolize to the individual. Are they unique to that person, or did others live through the same experience? Why did they choose each picture to represent that aspect or time in their lives? How did they cope, or what suggested ways of coping are there?
8. During the discussion, facilitators should discuss ways of challenging negative beliefs and overcoming negative memories. For example:

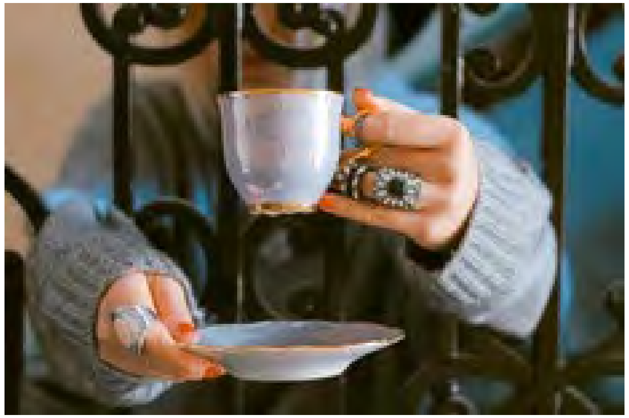

The statement **"I could not share utensils with my family members"** might trigger discussions around how people with TB can play an active role in protecting others from being infected, but how they cannot pass on TB by sharing utensils.

This exercise is based on concepts from the University of Cape Town's Memory Box community outreach initiative using the technique of "body mapping." http://www.davidkrut.com/pastExBodyMaps.html

Exercise 2.10 My right to tell <TB/DR-TB>

INTRODUCTION

The right to privacy is a fundamental human right. People should understand their right to privacy and feel empowered to make decisions carefully about the various levels of disclosure as it relates to them.

OBJECTIVE

To explore the tension between confidentiality, disclosure, and privacy and how they affect our lives. Also, to acknowledge the issues around disclosure and help participants understand the different stages of disclosure so they can make their own decisions about who to tell, how, and when/if.

Handout one copy of 'Adam's story' for each participant [Annex 8]. Handout one copy of the disclosure for each participant [Annex 9], or provide them with access to the participant handout booklet.

60 mins.

Handouts and discussion.

FACILITATOR'S STEP-BY-STEP INSTRUCTIONS

1. Divide the group into smaller groups of approximately eight participants. Hand out copies of the story. Ask one participant in each group to read the story.
2. Discuss the following questions in small groups:
   - Why does Adam feel the need to hide his TB treatment? Does he need to share his diagnosis anyway?
   - How do his worries about secrecy affect him? – look at now and in the future
   - Why is it important to tell someone?
   - Do situations like this happen in our communities? Share examples.
   - What can we do to support people on treatment to break through the secrecy?

   Bringing the group back to the large group, and ask each group to share their answers to the questions. Ask both groups about how living in secrecy may affect us.
3. Disclosure is a personal decision. This is not an exercise to recommend disclosure or to help people decide about disclosure. It is an exercise on awareness around disclosure.
4. Give participants the disclosure handout below and ask each of them to complete it for themselves, including writing down the pros and the cons in each disclosure category.
5. When everyone has finished, invite participants to share anything that was surprising.

Exercise 2.11 Staying in your own business: reducing stress <TB/DR-TB>

OBJECTIVE

To support participants to see how much time they waste worrying about the business of others, which may cause stress, and about things they cannot control. Participants will learn to focus on their own business, where they are able to reduce stress.

‘Three-kinds of Business’ handout [Annex 10].

20 mins.

Group discussion and participation.

FACILITATOR’S STEP-BY-STEP INSTRUCTIONS

1. Read the following text. Participants should have the handout

There are only three kinds of business in the universe: mine, yours, and God’s/ the Universe’s. (God’s being the Universe or any God you may have in your life, or a national state where individual choice is limited).

Whose business is it if I am feeling happy or sad? My business. Whose business is it if you are feeling happy or sad? Your business. Whose business is the weather? God’s/ the Universe’s business. (Anything that’s out of my control, your control, and everyone else’s control is God’s/ the Universe’s business.)

Much of our stress comes from mentally living out of our own business. When I think, “You need to get a job, I want you to be happy, you should be on time, you need to take better care of yourself,” I am in your business. When I’m worried about earthquakes, floods, war, or when I will die, I am in God’s business. If I am mentally in your business or in God’s business, the effect is separation and loneliness in my own life. If you are living your life and I am mentally living your life, who is here living mine? Being mentally in your business keeps me from being present in my own. I am separate from myself, wondering why my life doesn’t work. No one else causes my loneliness, only I can do that.

Notice when you feel loneliness or separation. Are you mentally out of your business? If you are not sure, stop and ask yourself, “Mentally, whose business am I in?” Notice when you give uninvited advice either out loud or silently. Whose business are you in when you are giving unsolicited advice?

EXERCISE

Whose business is it? Invite participants to do this exercise for themselves checking their answer off:

- My height.
- That I have TB.
- Attending a concert.
- Rush-hour traffic.
- My mother’s depression.
- Your judgements of me.
- My judgements of you.
- The weather.
- The effect of TB medicine.
- My friend’s anger.
- My anger.

Take away tip

When next you feel sad, hurt or stressed, ask yourself “whose business am I in?” If you find yourself in someone else’s business, gently bring yourself back to your own business.

Remember, other’s judgements of us can only have power if we believe them. We have no control over judgements made against us, but we can choose whether we believe them or not, or whether we apply meaning to those judgements.

Source: © 2016 Byron Katie International, Inc. All rights reserved. thework.com (slight adaptation made)

Exercise 2.12 My agency, my power <TB/DR-TB>

OBJECTIVE

To identify the self-limiting beliefs related to my TB recovery and to question them to feel more empowered and positive about the future.

4 x One Belief at a Time worksheets: http://thework.com/sites/thework/downloads/worksheets/onebelief_Eng.pdf

60 mins.

Discussion, group sharing, written exercise, and reflection (The Work of Bryon Katie).

FACILITATOR'S STEP-BY-STEP INSTRUCTIONS

1. Facilitators are encouraged to follow the script to get the most of this exercise.
2. Invite participants to reflect on their life with TB, and to close their eyes if they feel comfortable. They should examine who they were before their TB diagnosis, getting in touch with their dreams and the relationships they had (allow for some silence here as people contemplate).
3. Now invite them to look at themselves again, this time following the TB diagnosis.
4. Invite participants to make a list using the prompt: "Because I have (or have had) TB I am not able to…"
    - Examples for number four include "do the things I want to, further my education, play with my children, have a successful relationship, etc."
    - Another way to ask is "what can you not do or be because of your TB?" Facilitators need to be aware that there may also be additional treatment related disabilities that can exacerbate self-stigma or shame, such as infertility, deafness, unwanted (and sometimes uncontrollable) side effects of treatment, such as vomiting and depression, or extended dependency on an oxygen cylinder.
5. Invite participants to share their lists, encouraging them to add to their own lists as they hear things from others.
6. Choose a thought that has come up on a few lists, and ask 'how do you live your life when you believe the thought (for example 'I am not able to have a successful relationship)'. Invite people to share how they feel when they are believing that thought. Now ask them if they couldn't believe that thought for a moment, how would they live their life?
7. Invite participants to notice the effect on their life with the thought and without the thought.
8. Invite participants to circle the thought on their list that they feel is the **most stressful**. For example 'I am not able to have a successful relationship' or 'I am not able to have sex'.
9. Invite each participant to find a space to sit with their One Belief at a Time Worksheet and to take the belief that they have circled to the worksheet and write their answers on their sheet. When they get to the last part (the Turnarounds), remind them to find the opposite turnaround only and not to worry about finding all three for now. For example, 'I am able to have a successful relationship' and then find some examples of that (20 minutes).
10. When all participants have finished, have a round of sharing answers. Ask: "What Did You Learn? What Did You Find? What Did You Discover?"
11. Finish by ensuring –that there is life after TB, and although there could be residual physical changes, it is possible to find new ways to live.

SUMMARY

Unquestioned self-limiting beliefs can often have a huge impact on our lives. Identifying them and questioning empowers us and allows for a strong sense of agency.

Resources

Pennebaker. Writing to heal: A guided journal to recover from trauma and emotional upheaval. New Harbinger Publishers Inc., Oakland, CA. 2004.[20]

The Work of Byron Katie [available in over 50 languages] – www.thework.com

The Work for Change's work on self-stigma and shame in Zimbabwe and self-stigma and shame in Vietnam – www.theworkforchange.org

We are the change: Dealing with HIV-related self-stigma. Facilitators Guide Using The Work of Byron Katie: Inquiry-based stress reduction: www.thework-forchange.org

International HIV/AIDS Alliance Understanding and challenging HIV stigma. Toolkit for action – Module F: Coping with Stigma. Available at: https://www.aidsalliance.org/resources/370-toolkit-understanding-and-challenging-hivs-tigma[21]

de Bruyn, Maria and France, Nadine (2001). Gender or sex: who cares? Skills building resource pack on gender and reproductive health for adolescents and youth workers.[23]

Compton et al. Persuading others to avoid persuasion. Inoculation theory and resistant health attitudes. 2016. Frontiers in Psychology, 7, Article 122.[24]

University of Cape Town's Memory Box community outreach initiative using the technique of "body mapping". http://www.davidkrut.com/pastExBodyMaps.html

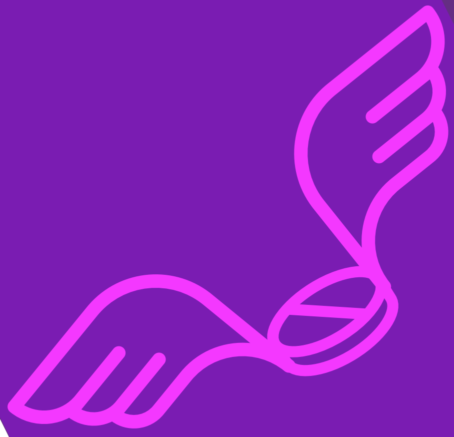

DRUG-RESISTANT TB (DR-TB) AND SELF-STIGMA

MODULE 3

In 2015, there were an estimated 480, 000 new cases of multidrug-resistant TB (MDR-TB) and an additional 100, 000 people with rifampicin-resistant TB.[25] In some areas, 30% of new reported TB cases showed some drug resistance, and it can affect up to 20% of previously treated people. For people with DR-TB, the treatment period is much longer than that for those with DS-TB, and is likely to last at least 18 months. This module will consider the impact of DR-TB on people receiving treatment, particularly the potential to increase self-stigma.

Drug-resistant TB (DR-TB) and self-stigma

Timeline:

Quarter of a day (2 hrs)

Required materials for this module:

Flip charts, marker pens, scenario handouts.

Introduction

Objectives of the module

This module is designed for people with DR-TB, and by the end of the module, participants will:

1. Begin to understand self-stigma associated with DR-TB.

2. Explore strategies that can help reduce the negative impact of self-stigma in the context of DR-TB.

Who will benefit?

This module is aimed at people with DR-TB who are in any stage in the treatment period. Ideally a person should complete the module early on, once they are culture-negative, which can be at six months or more.

"The unexamined life is not worth living"

– Socrates

Initial preparations

Welcome everyone to the session and outline the objectives of this module:

- Begin to understand self-stigma associated with drug-resistant TB.
- Explore strategies that can help reduce the negative impact of self-stigma in the context of drug-resistant TB.

Familiarize yourself with the participants, and find out what their TB experiences are. It may be useful to ask them what their hopes are for the session. This can be done through question and answer sessions, with responses written on a flipchart. Or, each person individually completes a post-it note and attaches it to the wall. These hopes can be revisited at the end of the session.

Remember that even though some workshop participants themselves may have been affected by drug-resistant TB, they might not necessarily have a lot of information about the disease. This module is information-heavy, and adequate time should be given to ensuring that participants understand the concepts and material, and that they do not have any misconceptions or wrong information.

Part I - Background presentation [see PowerPoint slides]

What is DR-TB? [Slides 2-5]

Drug-resistant TB (DR-TB) is caused by an organism that is not cured by one or more of the drugs used to treat TB. There are many forms of DR-TB, dependent on which class of drugs the organism is resistant to. There are mainly two categories of DR-TB important for our context:[26]

- Multi-drug resistant TB (MDR-TB) is when the bacteria causing the TB are resistant to at least isoniazid and rifampicin, the most important first line drugs against TB. Uncomplicated MDR-TB is where a patient is not resistant to second-line TB drugs.
- Extensively drug resistant TB (XDR-TB) is defined as when the bacteria causing TB are resistant to at least rifampicin and isoniazid, as well as second-line TB drugs; namely, one or more of the fluoroquinolones, and one or more of injectable TB drugs, such as Amikacin, Kanamycin, or Capreomycin.

In some contexts, people diagnosed with MDR-TB and XDR-TB benefit from a period of hospitalization for orientation and stabilization of their treatment.

It's important to be clear about what people know about DR-TB. DR-TB is a significant challenge in the context of TB support and care. DR-TB is more complicated to treat than 'drug-susceptible' TB, with an extended treatment period, reduced treatment outcomes, and increased risk of mortality. The cost of treatment is also more expensive.

Sometimes people with DR-TB may feel guilty about having acquired drug resistance (if due to earlier treatment non-adherence) or feel they may contaminate others because of the airborne transmission potential at the start of treatment. This can lead to the person avoiding social interactions even after the infectious period is over (usually six months), reduced well-being, depression, low self-esteem, reduced self-efficacy, poor long-term coping, and lower quality of life. Several papers have been published describing the impact of stigma and discrimination against people with DR-TB,[27, 28] but the impact of self-stigma is not specifically mentioned.

Drug resistance is more common in people who:[29]

- live in areas with a high prevalence of drug resistant TB
- are provided with the wrong treatment combination at initial diagnosis;
- live in an area where directly observed therapy (DOT) is not managed efficiently by medical/nursing staff;
- when there are drug stock outs;
- treatment involves counterfeit medications or drugs that are not quality assured;
- live in places where the first-line diagnostic tests do not measure resistance (e.g., smear microscopy);
- have spent time with someone known to have drug-resistant TB disease; and
- those who may not have taken their TB medicine as advised by their doctor or nurse.

It should be noted that although DR-TB can be due to the incorrect use of TB medicine somewhere in the treatment cascade (acquired resistance), it is possible to become infected with DR-TB from another person (primary resistance). The potential of acquired resistance can also be through sub-standard or incorrect medicines given to the person either by the health system or informal providers such as pharmacies (self-medication).[30] In spite of these varying scenarios, it is usually assumed that the drug resistance is due to poor treatment adherence, attracting stigma and amplifying the risk of self-stigma.

The medication to treat DR-TB can be difficult. It generally takes two years, 14,000 pills, and six months or more of daily injections before a patient can be cured of DR-TB. The drugs are powerful, toxic, and can cause debilitating side effects, including hearing loss, kidney problems, depression and psychosis:[31]

> *These drugs are so horrible to eat every day. After nearly a year and a half, I thought it was just too much; I couldn't keep taking all those pills. I thought it would be OK if I stopped taking them. But they told me if I didn't keep going I might get sick again and then I would have to start again from the beginning with all the injections. So, I kept going with the pills and now I am cured. It was such a long time.[32]*

Family caregivers can be of key benefit in providing emotional and psychological support.[33]

There are several side effects from the treatment of DR-TB. One study found the following (in order of frequency):[34]

- Gastrointestinal disturbances.
- Psychiatric disorders, such as depression, anxiety, and psychosis.
- Arthritis.
- Hepatitis.
- Peripheral neuropathy.
- Hypothyroidism.
- Epileptic Seizures.
- Dermatological effects.

Core issues in self-stigma and DR-TB [Slides 6-7]

According to the literature, there are issues amplifying the risk of stigma, and self-stigma, for people affected by DR-TB:

- **High risk of stigma** – being a 'special risk', self-exclusion, and a feeling of being a particular danger due to DR-TB [self-worth]. This is exacerbated by the extended period of treatment, and the necessity of wearing a mask whilst culture-positive. This will clearly mark him/her as different and increase the risk of self-stigma.
- **Attribution of blame** – DR-TB is often assumed to be the result of poor treatment adherence. Other external factors (such as substandard medication, or the person having primary resistance through contact with another person with DR-TB) are ignored. If the person blames him/herself, then this could lead to feelings of guilt and self-blame.
- **Misunderstanding the disease** – it is important to be open and talk to people about everything to correct their misunderstanding to help reduce rejection.
- **Self-isolation** - "Two participants mentioned that they deliberately separated themselves from friends and family to protect loved ones from potential infection. On further prompting, a few participants stated that while they understood health care workers had determined them no longer to be infectious, they were not willing to put their family at potential risk and preferred to stay away from others."[28]
- **Purpose to society** – there may be some economic impact due to the extended period required for treatment.

1 Adequate ventilation, cough 'etiquette' and correct disposal of sputum, preferably sleeping alone, avoiding public transport if possible, and spending as little time as possible in large groups of people.

Focus – extended treatment period, and beliefs about DR-TB [Slides 8-9]

The extended treatment period for DR-TB poses increased risks of stigma, with the person at risk of being 'visible' for longer, especially if there are side effects from the treatment. There are two **MYTHS** relating to DR-TB that are especially important for self-stigma:

1. The belief that DR-TB is more contagious than drug-susceptible tuberculosis.

BELIEF: People with DR-TB are more 'infectious' than those with drug responsive TB.

FACT: DR-TB carries a similar risk of infection as drug susceptible TB, and the precautions taken for drug-susceptible TB apply[1], but there are additional measures necessary when the person has culture-positive DR-TB:

- The person will need to wear a respirator while infectious.
- Family members living with HIV should not serve in caregiving roles for the person with DR-TB until all risk of infection is over.
- Children under the age of five should spend as little time indoors as possible with the patient until all risk of infection is over.
- These precautions are in addition to the standard precautions for TB.

An additional factor is that the period a person with DR-TB can be infectious is much longer than drug-susceptible TB. A patient with drug-susceptible TB on effective treatment can become non-infectious in as few as two weeks (though this can vary). However, for DR-TB, the infectious period can last six months,[35] and can pose an additional risk of mental health challenges.

2. That people with DR-TB have the disease because of failing to take their medication correctly.

BELIEF: People with DR-TB have this type of tuberculosis because of poor adherence to treatment and, by extension, a lack of personal discipline.

FACT: Although DR-TB can be the result of incorrect treatment, it is also possible to become infected with DR-TB from another person with this form of tuberculosis. One study from Eastern Europe showed that of 35% all new cases of MDR-TB (not XDR-TB) are in people who have never been treated for TB.[36]

Part II - Exercises

Exercise 3.1 The many faces of DR-TB <TB>

OBJECTIVE

To encourage discussion, reflection, and sharing of experience around self-stigma related to DR-TB, and to ensure personal beliefs do not increase feelings of shame.

Each case study should be available on a laminated card with the accompanying questions [see case studies in participant handouts] [Annex 11].

90 mins.

Group work, including discussion, reflection, and feedback.

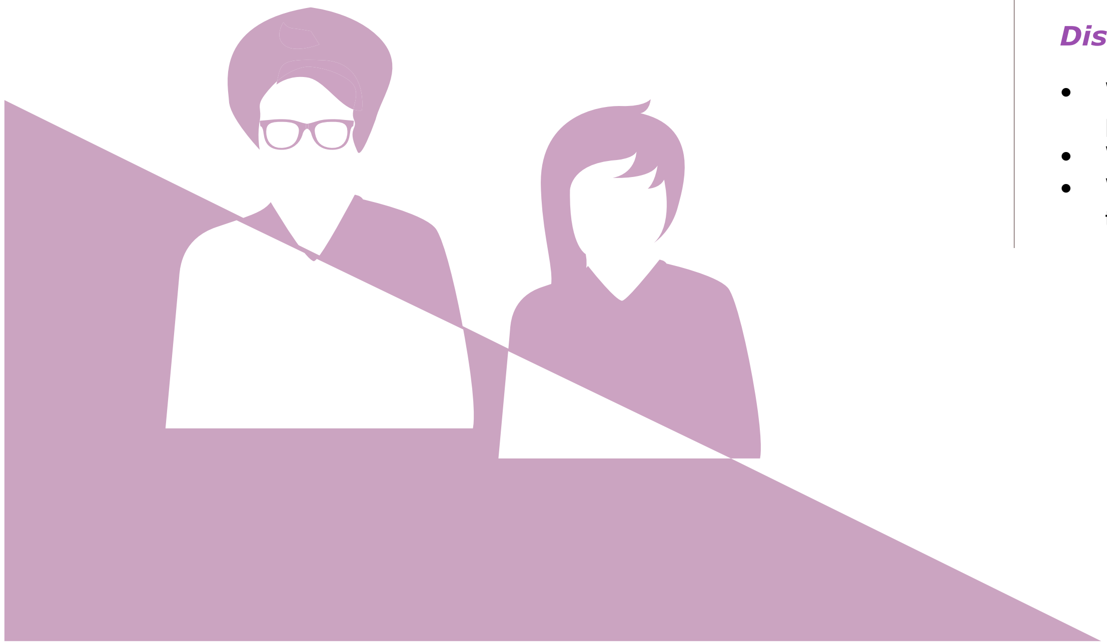

FACILITATOR'S STEP-BY-STEP INSTRUCTIONS

1. Divide into groups of no more than eight people each. Each group takes a case study. Invite each group to ask one person to read the story aloud. When the story has been read, invite each group to discuss the questions at the bottom. One person in each group will be responsible for reporting back to the larger group.

Discussion questions: Case Study 1

- Why does TB/MDR-TB create negativity/negative emotions in the life of patients?
- What helped Xolelwa deal with her MDR-TB diagnosis?
- Why would people feel self-stigma and shame with MDR-TB, and what can they do about it?

Discussion questions: Case Study 2

- Why does TB or DR-TB create negativity/negative emotions in the life of patients?
- What helped Rukmini deal with her DR-TB diagnosis?
- Why would people feel self-stigma and shame with DR-TB, and what can they do about it?

Part III - Session wrap

- Revisit the points of learning from the session. Go over each key point in turn, and make sure that the participants feel that they have achieved:
    - The predisposing factors leading to DR-TB.
    - The increased risk of self-stigma in the context of DR-TB.
    - The impact of self-stigma in DR-TB.
    - The myths relating to DR-TB.
    - The specifics of transmission control in relation to DR-TB.
- Confirm that participants are clear about DR-TB, and the impact on self-stigma.
- Clarify that participants understand the truths and false beliefs of DR-TB that can contribute to self-stigma, and the key points that arose during the discussions.

Resources

France, N. F. et al (2015). "An unspoken world of unspoken things": a study identifying and exploring core beliefs underlying self-stigma among people living with HIV and AIDS in Ireland.[1]

International HIV/AIDS Alliance (2009). Understanding and challenging TB stigma: Toolkit for action.[22]

International HIV/AIDS Alliance (2007). Understanding and challenging HIV stigma: Toolkit for action.[21]

TRANSMISSION CONTROL **AND SELF-STIGMA**

MODULE 4

Transmission control is a priority in the context of TB. This module aims to explore the impact of transmission control on the person, and the potential for increased self-stigma. Strategies for reducing self-stigma (by increased knowledge of TB transmission and the need for empowerment and agency) will be the focus.

Transmission control and self-stigma

Timeline:

Half a day (4 hrs)

Required materials for this module:

Flip charts, handouts of scenarios.

Introduction

Objectives of the module

This module is designed for people living with TB, and by the end of the module, participants will:

1. Begin to understand self-stigma associated with limited knowledge of transmission control.
2. Explore strategies that can help reduce the negative impact of self-stigma deriving from fears of being 'a threat' or risk to others.
3. To empower participants to feel greater agency and control over risk and masking.

Who will benefit?

This module is aimed at people receiving treatment for TB (once they are culture-negative) and can also be beneficial for TB survivors wishing to gain more information about TB risk.

> "If we can share our story with someone who responds with empathy and understanding, shame can't survive."
>
> – Brené Brown

Initial preparations

Welcome everyone to the session. Outline the objectives of this module

- Begin to understand self-stigma rooted in misunderstanding of transmission control.
- Explore strategies that can help reduce the negative impact of self-stigma deriving from fears of being 'a threat' or risk to others.

Familiarize yourself with the participants. Find out the particpant's TB experience and hopes are for the session. This can be done through:

- Question and answer session with responses written on a flipchart.
- Each person individually completing a post-it note and attaching to the wall.

These hopes can be revisited at the end of the session.

Part I - Background presentation [see PowerPoint slides]

What is TB risk? [Slides 2-4]

The germ that causes TB dies quickly with sunlight and ventilation. If a room is dark and unventilated, the TB germs can stay in the room for up to three days, thus increasing the risk of infection.

After two or three weeks of correct TB treatment, most diagnosed TB patients are no longer infectious.[22] DR-TB treatment is less effective and infectiousness can linger for six months.

Special precautions for preventing transmission: TB is a bacterium spread through the air by droplet. Another person who has breathed in a droplet can become infected. TB is not spread by:

- Shaking hands.
- Sharing food or drink.
- Touching toilet seats or bed linen.

Following a confirmed diagnosis of TB, the person is usually cared for at home. There are several precautions to take while the person is infectious:[37]

- Provide adequate ventilation.
- Practice cough etiquette and respiratory hygiene. Cover your mouth and nose when sneezing or coughing with a disposable tissue, if possible, and use proper sputum disposal. If this is not possible, cover your mouth or nose with your elbow or hand, and then wash them immediately.[38]
- The person should also:
    - Spend time outdoors.
    - Sleep alone, if possible, in a well-ventilated room.
    - Limit time on public transport, in hospitals, and in large crowds.

Once the person is non-infectious, they – and their families and friends – are aware that there is no risk of transmission from that point on. For drug-susceptible TB patients this is usually after two weeks, as advised by a health care worker, and determined by factors such as clinical improvement and/or negative sputum test. For drug-resistant TB patients this could be six months or more.

Knowledge about actual risk allows a person to take control of their environment. This will help avoid self-imposed isolation.

Beliefs about contagion and risk [Slides 5 and 6]

Beliefs about contagion and risk are complex. Although germ theory provides a rational interpretation of when someone is infectious or not, culture and lay beliefs[39] can play a significant part in risk perception. A 'disease' is categorized by medical science, but 'illness' is more of a cluster of metaphors, which reflect the personalities and social conditions of those who experience symptoms and treatments, as well as the people around them.

Beliefs about the causes of TB are varied. For example, in Ethiopia one study suggests that people believed TB could be caused by exposure to cold, excessive sun exposure, exposure to mud, khat, and inadequate food. Such beliefs initially led to self-treatment.[40] Studies have shown that some believe TB spreads in the same way as HIV,[41] or as a result of breaking cultural rules that demand abstinence from sex after a family death.[42] In a study from Vanuatu, contaminated food, sharing eating utensils, and kastom (sometimes translated as sorcery) were contributory factors.[43]

To ensure TB control, and reduce the impact of stigma, education about risk is vital. After two weeks of correct treatment the disease is almost gone from the air they exhale, and after two months it should be completely safe to be with other people. A sputum test can confirm.

Core issues around self-stigma and beliefs of infection and control [Slides 7 and 8]

People living with TB can believe they are a threat to others. One study found that some participants mentioned they separated themselves from friends and family to protect loved ones from potential infection.[44] This can happen despite being told by health care workers they were no longer infectious.[28, 45] For one person, "I was afraid of TB because I could transmit that to my son, daughter, and children."[45] It's important that support of people being treated for TB, especially around transmission control and risk of transmission, focus on empowerment and agency.

Part II - Exercises

Exercise 4.1 TB lifeline <TB>

OBJECTIVE

To ensure understanding around TB infectiousness at various stages of the disease and to identify any negative self-stigmatizing beliefs that may arise at particular points. Participants should appreciate that certain problems and feelings are time-sensitive. Their experience will change depending on disease stage.

TB Lifeline (either projected or draw onto a flipchart), My TB Lifeline copy for each participant.

90 mins.

Lifeline.

FACILITATOR'S STEP-BY-STEP INSTRUCTIONS

1. Show people the TB lifeline (Figure 11). Highlight the different phases, the duration of the different phases, and how people know where they are in the lifeline (by the various tests etc.) Note the differences between DR-TB and MDR-TB.
2. Invite each participate to draw their own TB lifeline (Figure 12). They should indicate the dates and length of the various stages in their own TB journey.
3. Then note the positive things that have happened during their journey on the top of the line (met new people, started treatment, went back to work, was able to attend a child's graduation, etc.) and also positive thoughts they had (I am starting to feel better, I am glad I know what it is, my family is supportive, I will get through this).
4. Then note the negative things that have happened during their journey on the bottom of the line (e.g., feeling very weak and sick, feeling guilty for using up family finances, isolation, depression, treatment is difficult to tolerate, reduced energy levels, etc.) and the negative thoughts they have had (I can't cope with this, it is my fault, I have let my family down, I can't provide for my kids, I am too weak to eat, people are afraid of me.)
5. Then answer the following questions:
   - What changed for them when they moved from the intensive phase to the continuation phase?
   - What was different during the two phases?
   - What specifically helped you to cope with the negative things that have happened?

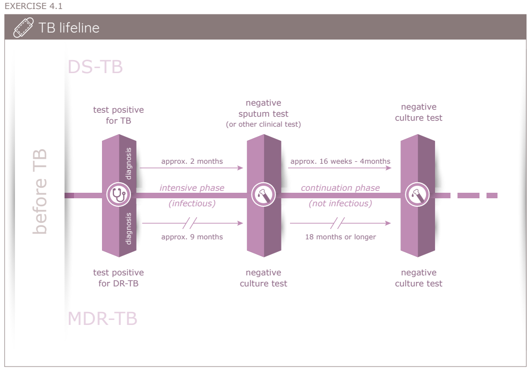

Figure 11. TB Lifeline example

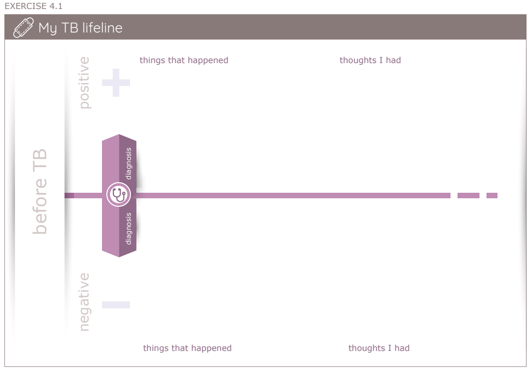

Figure 12. My TB Lifeline

6. Then invite some participants to share their TB lifelines.
7. Wrap up by sharing that many people find it difficult to not internalize negative thoughts, particularly when infectious and afraid of transmission to loved ones. We can carry some of these negative thoughts with us about contagion into the continuation phase. It is important to realize that that TB is a disease and not an identity.
8. TB is not me, and I am not TB.

Adapted from: Gender or sex: who cares? Skills-building resource pack on gender and reproductive health for adolescents and youth workers. de Bruyn, Maria and France, Nadine, 2001. Chapel Hill: Ipas & Health & Development Networks (HDN) Chiang Mai Thailand.[23]

Exercise 4.2 What do we know about TB and risk?

INTRODUCTION

This quiz [see Annex 12] can clarify TB facts. It can be used at any stage of this module, or to support other modules as required. Question groups in the quiz can be selected for particular modules (not all will be relevant for all modules).

OBJECTIVES

By the end of this session participants will be able to:

- ascertain the level of knowledge of TB; and
- provide 'correct' information about various aspects of TB.

Quiz handouts [see Annex 12].

45-60 mins.

Quiz and discussion.

FACILITATOR'S STEP-BY-STEP INSTRUCTIONS

It's important that people are not 'penalized' for getting answers wrong. The recommended approach to the exercise is:

1. Ask participants to complete the questionnaire individually.
2. Go through the answers as a group discussion. People can amend/correct their answers individually.

Part III - Session wrap

- Revisit the points of learning from the session. Go over each key point in turn, and make sure that the participants feel that they understand:
    - The risks of TB transmission.
    - Risk and contagion.
    - The impact on self-stigma.
- Confirm that participants are aware of infection risk and TB.
- Clarify that participants understand the truth about TB risk, as false beliefs can contribute to self-stigma.

Resources

CDC (2016). TB Elimination: General Considerations for Treatment of TB Disease. Online at: https://www.cdc.gov/tb/publications/factsheets/treatment/treatmenthivnegative.pdf

de Bruyn, Maria and France, Nadine (2001). Gender or sex: who cares? Skills-building resource pack on gender and reproductive health for adolescents and youth workers.[23]

France, N. F. et al (2015). “An unspoken world of unspoken things”: a study identifying and exploring core beliefs underlying self-stigma among people living with HIV and AIDS in Ireland.[1]

International HIV/AIDS Alliance (2007). Understanding and challenging HIV stigma: Toolkit for action.[21]

Simbayi, L. C. et al (2007). Internalized stigma, discrimination, and depression among men and women living with HIV/AIDS in Cape Town, South Africa.[46]

Health rights, TB, **AND SELF-STIGMA**

MODULE 5

This module explores the connection between self-stigma and the need for an awareness of basic rights. It focuses on the right to health, and is designed to increase understanding into ways to maximize health rights for people affected by TB. The module is based on the fact that the right to health is a fundamental human right. Protecting and ensuring full realization of the right to health should be the primary responsibility of the state.

Health rights, TB, and self-stigma

Timeline:

Half a day (4 hrs)

Required materials for this session:

Flip charts, handouts of scenarios, cards (for Exercise 5.4)

Introduction

Objective of the module

Deeply exploring self-stigma can leave a person clear minded, strong, and able to fight for their rights. This module on human rights is aimed at ensuring people understand their rights regarding TB and that they can see redress if their rights are being violated.

1. Understand the right to health in the context of self-stigma associated with MDR-TB and other forms of drug-resistant and drug-sensitive TB.
2. Explore strategies that can help ensure better realization of the right to health and mitigate violations of these rights, thereby reducing the negative impact of self-stigma.

Who will benefit?

This module is for people currently being treated for TB, TB survivors, carers, policy makers, and advocates.

"Shame corrodes the very part of us that believes we are capable of change."

– Brené Brown

Initial preparations

Welcome everyone to the session. Outline the objectives of this module

- Begin to understand health rights in the context of self-stigma associated with MDR-TB and other forms of drug-resistant and drug-sensitive TB.
- Explore strategies that can help ensure better realization of health rights and to mitigate violations of these rights, thereby reducing the negative impact of self-stigma.

Familiarize yourself with the participants. Ask them about their TB experiences and their hopes for the session. This can be done through:

- Question and answer sessions, with responses written on a flipchart.
- Each person writes a hope on a post-it note that is attached to the wall.

These hopes can be revisited at the end of the session.

Part I - Background presentation [see PowerPoint slides]

What are rights about TB? [Slides 2-3]

The right to health is a fundamental human right. The right to health includes access to timely, acceptable affordable health care. The human rights-based approach should be at the core of TB care, which can help address inequalities, discriminatory practices, and unjust power relations, which are often at the heart of inequitable health outcomes.

Even the constitution of the WHO has the right to health principle enshrined: "…the highest attainable standard of health as a fundamental right of every human being." Health is a state of complete physical, mental, and social well-being, and not merely the absence of disease or infirmity.

Exercising the right to TB care without discrimination [Slides 4-5]

The Committee on Economic, Social, and Cultural Rights underlined non-discrimination as one of the key interventions to protecting the right to health.[47] The principle of non-discrimination seeks "to guarantee that human rights are exercised without discrimination of any kind based on race, color, sex, language, religion, political or other opinion, national or social origin, property, birth or other status such as disability, age, marital and family status, sexual orientation and gender identity, health status, place of residence, economic and social situation."

TB-related stigma, especially self-stigma, is a major but neglected barrier to accessing existing care services, and violates human rights to health.

The Charter on Rights and Responsibilities of TB patients [Slides 6-7]

In 2006, TB patients developed the Patients Charter for TB Care (The Charter) via an inclusive and consultative process which complemented the International Standards for TB Care (ISTC) and fed into the national TB programs in high burden nations as well as the WHO Stop TB Strategy. The Charter outlines the rights and responsibilities of people with TB. Some of these rights enshrined in The Charter include:

- The right to free and equitable access to TB care, from diagnosis through treatment completion, regardless of race, gender, age, language, legal status, religious beliefs, sexual orientation, culture, or having another illness.
- The right to be treated with respect and dignity, including the delivery of services without stigma, prejudice, or discrimination by health providers and authorities.
- The right to quality healthcare in a dignified environment, with moral support from family, friends, and the community.
- The right to job security after diagnosis or appropriate rehabilitation upon completion of treatment.

NB: It's also important to ensure there is right to confidentiality and informed consent for all people affected by TB [This is covered in more depth in Module 2 exercise 2.10.]

The World Health Organization[48] has four key principles underpinning the End TB Strategy:

1. Government stewardship and accountability, with monitoring and evaluation.
2. Strong coalition with civil society organizations and communities.
3. Protection and promotion of human rights, ethics, and equity.
4. Adaptation of the strategy and targets at the country level, with global collaboration.

Core issues on right to health in relation to self-stigma and TB [Slides 8-9]

There are laws, policies, and practices in TB management that can amplify the risk of human rights violations and self-stigma.

The Right to health framework differs in different countries based upon their legal and constitutional framework, but government obligations to the right to health as enshrined in different international policies, agreements, treaties, declarations, the WHO constitution, and the UN Charter should take primacy over other considerations, such as trade.

Since 2006, the Patients' Charter for TB Care has been part of the WHO Stop TB Strategy and was adapted by different national TB programs. The WHO End TB Strategy, which was unanimously adopted at the 2014 World Health Assembly, has a strong commitment to end catastrophic costs along with care and support for people with all forms of TB.

There are several other obligations governments have committed to enshrined in various international policies, declarations, agreements, and policy statements to protect the rights of women, transgender people, or people living with specific conditions, such as disabilities, or specific populations, such as aborigines. Governments are commitments to achieve the UN Sustainable Development Goals (SDGs) to achieve Universal Health Coverage (UHC) as well as end TB by 2030.

The compelling need for integrated development has never been so acute. No one goal can be achieved unless we make progress on all 17 SDGs. Right to TB care needs to include components such as mental health, disability rights, welfare rights, gender justice, social security, urban development, right to food, and right to housing, among others.

But country-level approaches are still fragmented, and as a result a person with TB can "suffer both from the disease itself and its impact on their enjoyment of other human rights. They may also be subjected to involuntary hospitalization, isolation, and incarceration. TB- associated stigma and discrimination and their overlap with discrimination based on poverty, HIV status, gender, or belonging to other marginalized groups, erect barriers to accessing treatment and care. For the global TB response to succeed, these issues have to be immediately addressed with human rights-based interventions."[49]

Addressing self-stigma can enable a person to fight for their rights. This module on human rights aims to help people understand their rights with TB and to know where they can go for redress if their rights are being violated.

Part II - Exercises

Exercise 5.1 Rights and the patient with TB <TB>

OBJECTIVE

To explore rights and TB in the context of potential self-stigma.

Handout of case studies, flip chart.

60 mins.

Case studies

FACILITATOR'S STEP-BY-STEP INSTRUCTIONS

The right to health includes access to timely, acceptable, and affordable health care of appropriate quality. TB care should have a human rights-based approach in its core to address inequalities, discriminatory practices, and unjust power relations, which are often at the heart of inequitable health outcomes.

Given that all rights are inter-related and interdependent, it's important for facilitators to explore those that may impact on the right to health, such as education, safe employment, or equality under the law.

1. Which rights are violated? [Buzz groups]

Divide the group into pairs, and ask them to discuss:

- What rights could be violated if we have TB?
- How are these rights violated?
- How do I feel then they are violated?

EXAMPLES: the rights to information, health care, work, confidentiality, informed consent, and privacy.

'How it makes me feel?' Examples could be: lacking influence, feeling separate, feeling judged, self-blame.

2. Finding solutions

Divide into small groups and provide each group with some case studies. Ask them to read the case studies and discuss:

- Which rights have been violated?
- Have you experienced a similar situations?
- What could you do if you were the person in the case study?
- Do you have other examples that can be shared from personal experience?

3. Report back and discussion

Ask groups to present their key points for discussion, providing key approaches they have to challenge the violation. Focus on factors such as:

- How did each situation make the affected people feel?
- What impact would it have on their feelings of self-worth or shame?
- How might the solutions help people become less self-stigmatizing?
- What actions can be taken, and what are the avenues to explore when rights are violated?

Facilitators should explore the responses from individuals and groups. Discuss the key issues around rights to health and other rights that impinge in people affected by TB, such as broader issues like privacy, control of personal information, or informed consent.

SUMMARY

- People with TB have rights, and patients have the right to participate in all decisions related to their health
- Violation of rights can harm health and finances, and amplify the risk of self-stigma where a person could isolate themselves through self-blame (and be unable to protect their rights).
- Patients can be involved in decision making about their lives and care decisions
- The denial of rights can lead to reduced empowerment, the freedom to make decisions, and the potential for significant self-stigma.

Exercise 5.2 Perfectly Imperfect

OBJECTIVE

The object of the game is to learn to deflect attempts to stigmatize people with TB (represented by stigma cards: blame, stereotypes, guilt, and lies) and to move your character toward peace (by gathering as many peace points from help cards: truth, love and justice). It should be used to consider rights in the context of TB and the impact of self-compassion. Many of the 'help' contents relate to information about TB. This is related to ensuring a patient's rights are protected.

NB: This card game also works in other Modules where the clarity about the impact of TB and self-compassion is addressed, for example Modules 3, 4, and 6.

How to achieve peace of mind and body

Players need to counter stigma cards dealt by the designated 'stigmatized' player by responding with a corresponding help card, representing heart (love), head (truth), or hands (justice). Players who play the most applicable card get to keep their winning card and add its peace value towards winning the game. The highest score wins after all stigma cards have been played.

Cards [Annex 14]

Variable

Card game

NB: Cards – if possible, these should be printed in color, on high-quality card so that they can be easily re-used.

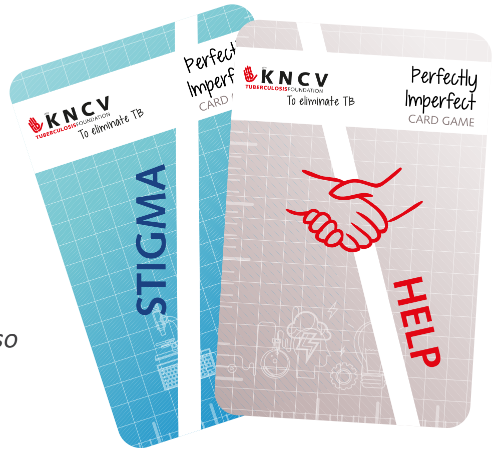

Playing the game

Play mode

There are three play modes to choose from:

A. Discussion Style. The 'Stigmatized player' (the one playing the stigma card) judges whose help card is the most effective. That player wins that round.

B. Points Style. Players add peace values (in the upper left corner of help cards) to match or exceed the stigma value (in the upper right corner of stigma card). Players play one at a time, starting to the right of the Stigmatized player. The player with the highest value wins that round.

C. Match Style. Players must match their help card to the sigma type/or specific card by matching the icon at the bottom of their help card to the icon in the top left of the stigma card. The first player to match wins that round.

Setup

At the start of the game, make a deck of 'stigma' cards and 'help' cards. Deal each player one 'stigma' card and three 'help' cards. Then shuffle both decks together and place the deck in the center of the table. The dealt cards are held in hand, only for the player to see. The grumpiest player goes first.

Rounds

Each round is started by the player holding the 'stigmatized' card. He/she plays a 'stigma' card (blame, stereotype, guilt, or lie) face up from their hand.

MODE A (Discussion Style)

All players choose the one help card from their hand that is the most logical/meaningful response to the stigmatizing message. The stigmatized player discusses which help card she/he finds most appropriate and chooses the winning card. The player who had this help card gets the 'stigma' card and places it in front of him/her with his/her own 'help' card on top of it.

At the end of the game, the player who took the most 'stigma' cards is the overall winner. If there are two players at the end of the game who have taken equal numbers of 'stigma' cards, they should add up the point values on those stigma cards. The player who has the highest total points from the stigma cards is the winner.

MODE B (Points Style)

The player to the stigmatized player's right must put down one 'help' card (heart, head or hands).

- If the peace value on the help card is equal to or higher than the stigma value, the player who played the 'help' card gets the 'stigma' card and places it in front of her with her own 'help' card on top of it.
- If the peace value on the help card is less than the stigma value, the player to the right of him/her must now play a 'help' card. This 'help' card's peace value is added to the first 'help' card's peace value. Continue this way until:
    - The peace value is equal to or higher than the stigma value, at which point the player who added the 'help' card with the highest peace value gets the 'stigma' card and only places his/her winning 'help' card on top of it. The other 'help' cards are discarded. In case of a tie in peace values, the first player is the winner.
    - All players have put down a card ('stigma' or 'help'). All cards played this round are discarded and no one wins. For two or three players, you may want to go a second round to try and 'beat' the Stigma value.

Note: Immediately after playing a help or stigma card, the player must draw a new card from the deck.

The next round is started by the player who won this round. She/he gets the stigmatized card to indicate they are playing the stigma card. If no one won this round, the stigmatized card goes to the player to the left of the current stigmatized player.

MODE C (Match Style)

All players quickly choose one help card from their hand which contains a stigma icon (at the bottom) that matches the icon on the stigma card (left upper corner). The player whose help card matches the 'stigma' card wins the round and places it in front of him/her with his/her own 'help' card on top of it.

Winning

When the last Stigma card has been played, the game ends and the players tally how many peace points they've accumulated. The player with the most heart points is the most empathetic player, the one with most head points is the most knowledgeable player, and the one with the most justice points is the most righteous player.

For example (MODE B)

- Dennis plays a 'stigma' card that says "BLAME: X got TB from hanging out in the wrong places."
- Anna (to his right) plays a 'help' card that says "TRUTH: TB is caused by breathing, it is nobody's fault," It has a peace value of 5.
- Because Anna won the last round, she now plays a 'stigma' card that says "LIE: It is natural to shun people with TB because it is airborne." It has a stigma value of 8.
- Mark (to her right) tries to counter it with a 'Help' card that says "TRUTH: After two weeks of treatment, people are usually not infectious." It has a peace value of 6. Mark knows it's not yet enough to win this round but hopes no one will play a higher peace value 'help' card.
- Jenna (to Mark's right) must play a 'help' card and plays "ACTION: Hand out a brochure about the facts of TB." It has a peace value of 2 (her lowest card value).
- Anna determines which counter is the most effective response to the stigmatization, and that player gets the points on the card.

Part III - Session wrap

- Revisit the points of learning from the session. Go over each key point in turn, and make sure that the participants feel that they have achieved their goals.
- Confirm that participants are clear about the rights of TB patients, and the impact of these rights on self-compassion.

Resources

UN Committee on Economic, Social and Cultural Rights (CESCR), General comment No. 20: Non-discrimination in economic, social and cultural rights (art. 2, para. 2, of the International Covenant on Economic, Social and Cultural Rights), 2 July 2009, E/C.12/GC/20, available at: http://www.refworld.org/docid/4a60961f2.html [accessed 4 October 2018]

World Health Organization (2006) The Patients' Charter for Tuberculosis Care. World Health Organization, Geneva. 2006. Available at: http://www.who.int/tb/publications/2006/istc_charter.pdf

World Health Organization (2017). Ethics Guidance For The Implementation Of The End TB Strategy. Online at: http://www.who.int/tb/publications/2017/ethics-guidance/en/

UNDP (2017). Legal Environment Assessments For Tuberculosis: An Operational Guide. Online at: http://www.stoptb.org/assets/documents/communities/StopTB_TB%20LEA%20DRAFT_FINAL_Sept%2027.pdf

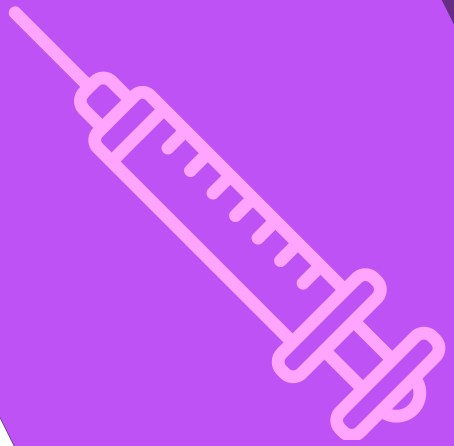

TB TREATMENT AND **SELF-STIGMA**

MODULE 6

TB treatment offers infected people the chance of a full recovery, if medication is taken for the full course. Taking treatment does pose challenges, including remembering when to take tablets, side-effects, and information control (determining when, and how, details of an infection are shared beyond close family and friends). People finding it difficult to adhere to a treatment program are at risk of developing a resistant form of TB. Throughout treatment self-stigma can be significant.

TB treatment and self-stigma

Timeline:

Quarter of a day (2-3 hrs)

Required materials for this module:

Flip charts, marker pens, handouts of scenarios.

Introduction

Objectives of the module

This module is designed for people being treated for TB. By the end of the module, participants will:

1. Begin to understand and manage the stigmatizing side effects of TB.
2. Explore strategies that can help develop self-compassion in the context of TB treatment.

A separate module is available in this package that focuses specifically on issues around stigma and drug resistant TB (DR-TB).

Who will benefit?

This module is aimed at all people receiving treatment for TB, and those who are culture negative. It aims to increase the understanding of TB treatment and the impact of treatment on a person, as well as the risk of increasing self-stigma. The side effects of TB treatment will be explored, and attendees will develop strategies to practice self-compassion.

"Don't believe Everything You Think."

– Byron Katie

Initial preparations

Welcome everyone to the session. Outline the objectives of this module

- Begin to understand self-stigma associated with taking treatment for TB.
- Explore strategies that can help reduce the negative impact of self-stigma in the context of TB treatment.

Familiarize yourself with the participants by finding out more about their TB experiences and hopes for the session. This can be done by:

- Question and answer sessions, with responses written on a flipchart
- Individuals completes a post-it note and attach it to the wall.

These hopes can be revisited at the end of the session.

Part I - Background presentation [see PowerPoint slides]

Treating TB [Slides 2-3]

What drugs are used?

Drug-susceptible TB disease can be treated by taking several drugs for six to nine months. The first-line anti-TB agents that form the core of treatment regimens are:

- Isoniazid (INH).
- Rifampin (RIF).
- Ethambutol (EMB).
- Pyrazinamide (PZA).

Treatment of drug-resistant TB (DR-TB) will require a longer treatment period, often two years or more, due to delays in being mistreated as drug-susceptible and waiting for confirmation of drug resistance.

What are the side effects of treatment for drug-susceptible TB?

Common side effects of TB treatments include:

- Nausea or dizziness.
- Skin rashes.
- Pins and needles.
- Influenza-like symptoms.
- Rarely jaundice.

Other side effects can include:

- Orange body fluids, such as urine, saliva, and tears (where soft contact lenses can be stained) is associated with **Rifampicin**. This may stain clothing.
- Sun sensitivity may require strong sunscreen to avoid burning.
- Reduced effectiveness of contraceptive pills and implants for Rifampicin. Women may be asked to use another form of birth control.

2 Cavitary pulmonary TB: spaces (open areas) in the lungs caused by the TB bacterium.

Other factors during the treatment period [Slide 4]

Phases: There are two phases of treatment of drug-susceptible TB:[50]

1. An **intensive phase** of two months when all four medications are given. It is during this phase that the diagnosis, and living with TB, can be most disruptive. The person may not feel 'well' for some time, and the impact on financial and social well-being can be difficult.
2. A **continuation phase** of four months when two medications are given, usually Rifampicin and Isoniazid (sometimes extended to seven months if there are complicating factor such as cavitary pulmonary TB[2] and when the sputum culture at two months remains positive). During this phase, the person should feel stronger and more able to live a normal daily lives. Self-stigma could lead to a tendency to continue social isolation, even though the person at this stage will not be infectious.[45] There may also be pressing financial problems caused by inability to work during the early stages.

Special precautions for preventing transmission [Slides 5 and 6]

TB is a bacterium spread through the air by droplet. Another person who has breathed in a droplet can become infected. TB is not spread by:

- Shaking hands.
- Sharing food or drink.
- Touching toilet seats or bed linens.

Following a confirmed diagnosis of TB, the person is usually cared for at home. There are several precautions to take while the person is infectious:[37]

- Provide adequate ventilation.
- Practice cough etiquette and respiratory hygiene. Cover your mouth and nose when sneezing or coughing with a disposable tissue, if possible, and use proper sputum disposal. If this is not possible, cover your mouth or nose with your elbow or hand, and then wash them immediately.[38]

- The person should also:
    - Spend time outdoors.
    - Sleep alone, if possible, in a well-ventilated room.
    - Limit time on public transport, in hospitals, and in large crowds.

Once the person is non-infectious, they – and their families and friends – are aware that there is no risk of transmission from that point on. For drug-susceptible TB patients this is usually after two weeks, as advised by a health care worker, and determined by factors such as clinical improvement and/or negative sputum test. For drug-resistant TB cases this could be six months or more.

Core issues around self-stigma and TB treatment [Slides 7-8]

There are several core issues that link treatment with self-stigma. The potential causes of self-stigma in relation to treatment can include:

- Changes in body image (such as skin rash, weight changes due to nausea, or, in extreme cases, jaundice, hearing loss, or infertility).
- Increased vulnerability to deductive disclosure during the treatment period because symptoms are harder to conceal.

The consequences of self-stigma amplified by TB treatment can include:

- A tendency to self-isolate and assume judgement from others, leading to reduced social capital and emotional support.
- Difficulties generating an income due to self-isolation.
- The possibility of a negative body image, especially when side effects of treatments are evident.
- A lack of energy for self-care, which could reduce willingness to attend health centers.
- If they are the only person with TB in their immediate community, being on treatment is a reminder they are 'different' from other people.[3] [51, 52]
- Less control over disclosure and agency (being 'seen' while taking treatment) and therefore amplifying the risk of social separation and self-induced isolation.

---

3 Building social networks for people on treatment for TB can help ameliorate this, and certainly has a positive impact on treatment adherence (Munro et al, 2007) and does in the HIV context (Hodgson et al, 2012)

Part II - Exercises
Exercise 6.1 TB self-stigma and treatment <TB>

INTRODUCTION

This is a good starter exercise for focusing on treatment and stigma. It identifies self-blame, anticipated stigma, stigmatization, and low self-esteem associated with mistreatment.

OBJECTIVES

By the end of this session, participants will be able to:

- Understand how stigmatization and self-stigma can affect access to treatment.
- Share ideas and experiences of self-stigma related to TB treatment.

Flipchart, card and markers.

45-60 mins.

Discussion and a question and answer session.

FACILITATOR'S STEP-BY-STEP INSTRUCTIONS

1. Buzz and card storm

- Distribute cards and markers. Ask participants to buzz (i.e. brainstorm by passing ideas back and forth) with the person next to them some of the ways in which stigmatization, and self-stigma, affects effective TB treatment. Write one point per card.
- Stick cards up and ask participants to help cluster cards with similar points together.
- Divide into small groups and give each group one of the clusters to analyze. Have the groups share stories and experiences to try and understand the problems further.

2. Report back

- Groups can present summaries of their discussions in any way they choose (flipchart, story, role-play, etc.)

3. Processing

- Ask participants:
    - What do we learn from this?
    - What are the initial ideas about how we could change things?

Examples of stigmatization by others

- When attending for directly observed therapy (DOT), the health care worker makes you feel as if you are not trustworthy enough to care for yourself, and you begin to feel disempowered.
- The church teaches you that you need prayer or 'cleansing' instead of treatment, and you begin to doubt your judgment.
- The health care workers think you caused the DR-TB by not taking your medicine consistently in the first round, which triggers feelings of guilt and shame.
- There are myths and rumors about treatment side effects, as well as difficulties surrounding some of the potential side effects.
- The family doesn't want to spend money to support treatment.[22]

The Allies Approach is a curriculum to reduce stigmatizing behavior and policies in health care facilities. It is available here: www.kncvtbc.org/stigma

Exercise 6.2 Treating my body, treating my mind <TB>

INTRODUCTION

Timely and quality TB treatment is vital to recovering from TB. In many cases, TB treatment can last between 6 to 24 months. People often have strong views or self-limiting beliefs about taking medication related to dependency on medication, side effects, and secrecy. It is important to support participants to identify their own self-limiting beliefs and to understand the resulting effects on their lives. People working through this toolkit should expect to experience feelings of discomfort and resistance from within.

OBJECTIVES

By the end of this session, participants will have:

- Identified self-limiting beliefs about TB treatment and understand the effect of those beliefs on their lives.
- Identified empowering beliefs for a support during treatment.

Flip chart - Treatment self-limiting beliefs.

60-90 mins.

Group coaching

FACILITATOR'S STEP-BY-STEP INSTRUCTIONS

1. Invite participants to think about their TB treatment, including the number of pills they must take and the time of day they must take their medication. Now invite them to write a list of all the negative thoughts.

   I am taking TB treatment and that means that (see examples below):

   - My body will experience side effects.
   - I will never be the same again.
   - I can't tolerate the side effects.
   - I am weak.
   - I might not be able work if I cannot hear.
   - I will forget to take my medications, and they won't work.
   - I am putting 'poison' into my body.

2. Now invite volunteers to share their answers. Facilitators should capture common beliefs on a post-it on a flip chart.
3. When the group has shared their answers, facilitators should choose one of the beliefs that came up more than once (for example: 'I will never get my appetite back'.)
4. Ask the group:

- What is the effect of believing this thought?
- How do you feel when you believe this thought?
- How do you treat yourself when you believe the thought?
- How do you treat your TB medication when you believe the thought?

5. Now imagine who you would be without this thought. How does it influence your attitude, behavior, and life?
6. Invite the participants now to look at their lists and draw a line down the center of their page. Then to turn each belief written on the left-hand side to the opposite and put it on the right-hand side. For example:

Self-limiting beliefs	Empowering beliefs
I am putting 'poison' into my body	I am putting tonic into my body
People will know my TB status	People will not know my TB status
My body will experience side effects	My body will not experience side effects
I will never be the same again	I will be same again
I can't tolerate the side effects	I can tolerate the side effects
I am weak	I am strong
They might not work	They might work
I will forget to take my medication and they won't work	I will not forget to take my medication and they will work

7. Now invite participants to put a line through the self-limiting belief and to look at the empowering belief they have replaced it with. They should turn to a partner and read each empowering belief, finding three examples where the empowering belief is true for them.

   For example: I am weak is turned to I am strong.

8. This exercise is useful in noticing how self-limiting beliefs about TB treatment have a profound effect on our lives. We do have a choice in what we believe once we become more aware of our thinking.

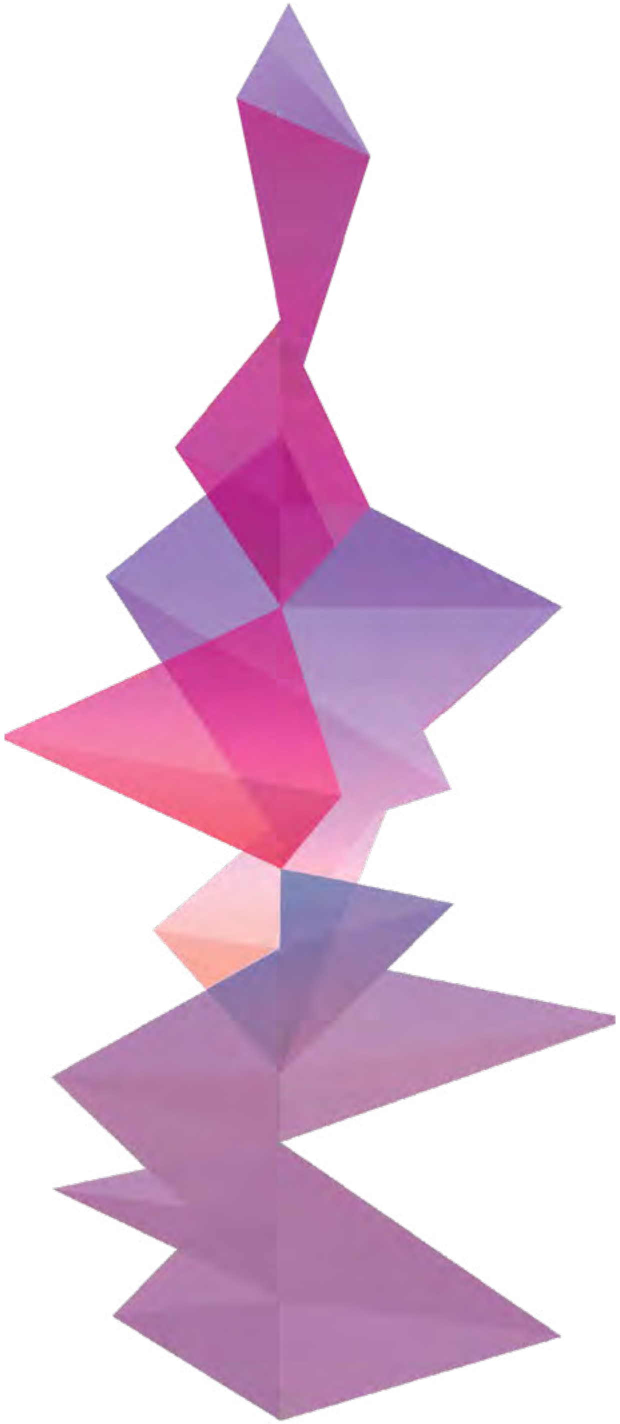

Inspired by:
The Work of Byron Katie - www.thework.com
Positive Success Group Coaching - https://www.positivesuccessgroup.com

Part III - Session wrap

- Revisit the points of learning from the session. Go over each key point in turn, and make sure that the participants themselves feel that they understand:
    - The types of TB treatment.
    - The impact of TB treatment on self-stigma, especially on how self-beliefs can sometimes predispose one to self-stigma.
    - The impact of self-stigma on the effectiveness of treatment.
- Confirm that participants are clear about TB treatment and the impact on self-stigma
- Clarify that participants understand how false beliefs about TB treatment can contribute to self-stigma.

Resources

CDC (2016). TB Elimination: General Considerations for Treatment of TB Disease. Online at: https://www.cdc.gov/tb/publications/factsheets/treatment/treatmenthivnegative.pdf

France, N. F. et al (2015). "An unspoken world of unspoken things": a study identifying and exploring core beliefs underlying self-stigma among people living with HIV and AIDS in Ireland.[1]

International HIV/AIDS Alliance (2007). Understanding and challenging HIV stigma: Toolkit for action.[21]

Simbayi, L. C. et al (2007). Internalized stigma, discrimination, and depression among men and women living with HIV/AIDS in Cape Town, South Africa.[46]

International HIV/AIDS Alliance (2009). Understanding and challenging TB stigma: Toolkit for action. [22]

The Work of Byron Katie - www.thework.com

Positive Success Group Coaching - https://www.positivesuccessgroup.com

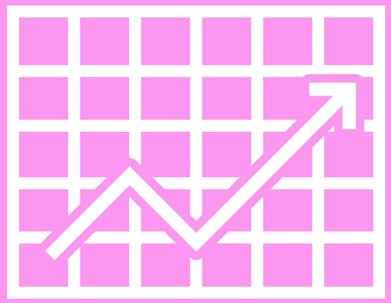

PLANNING FOR THE FUTURE - **TB FREE! WHAT NOW?**

MODULE 7

Reaching the end of TB treatment is an important milestone for people with TB. It is an opportunity for reflection, self-appreciation, and for moving on as a stronger, more self-aware person. Marking the end of treatment is also an opportunity to make sure that any shame and feelings of self-stigma that may have surfaced during the treatment phase have been dealt with. This module will enable transition to a ‘post-TB’ phase.

Planning for the future - TB Free! What now?

Timeline:

Quarter of a day (2-3 hrs)

Required materials for this module:

Flip charts, marker pens.

Introduction

Objectives of the module

By the end of the module, participants will:

1. Explore ways to plan for a TB-free life, particularly around feelings and thoughts leading to self-stigma.

2. Identify factors in their own lives that could impact their social and mental health following TB treatment.

Who will benefit?

This module is designed for people who are near the end, or have recently completed, their TB treatment phase. Transitioning from a 'TB' to 'TB-free' identity requires adjustment and adaptation for people who may have experienced self-stigma during their treatment phases.

"It's not all bad. Heightened self-consciousness, apartness, an inability to join in, physical shame and self-loathing–they are not all bad. Those devils have been my angels. Without them I would never have disappeared into language, literature, the mind, laughter and all the mad intensities that made and unmade me."

– Stephen Fry

Initial preparations

Welcome everyone to the session. Outline the objectives of this module

- Explore ways to plan for a life post-TB, particularly around leaving behind feelings and thoughts that may lead to self-stigma.
- Identify factors in their own lives that could impact their social and mental health following TB treatment.

Familiarize yourself with the participants: find out about their TB experiences and hopes for the session. This can be done through:

- Q&A, with responses written on a flipchart.
- Each person individually completing a post-it note and attaching it to the wall.

These hopes can be revisited at the end of the session.

Part I - Background presentation [see PowerPoint slides]

The issues - transitioning from patient back to person [Slides 2-3]

TB, even when successfully treated, may change a person's life. Physically there may be residual symptoms, such as tiredness, and the person's social life may not immediately return to as it was prior to a TB diagnosis. For a period after treatment, a person may find it difficult to talk about their experience.[53] Increasing empowerment and engaging with supportive social and family networks can be beneficial.

In the context of self-stigma, it's vital to ensure that people can process and confront predisposing factors, especially around self-awareness and a refusal to be bound by the narratives of other people.

Part II - Exercises

Exercise 7.1 The end of my TB journey - moving on <TB>

OBJECTIVE

To mark the end of and reflect upon the TB journey, enabling participants to move on emotionally and physically from their TB experience.

Several objects, preferably from the local environment, such as rocks, stones, flowers, grass, and sand. Also pens, rulers, and pencils. Should have colored markers and paper available too.

Flip chart with points for writing a letter

60-90 mins.

FACILITATORS STEP-BY-STEP INSTRUCTIONS

1. The end of TB treatment is an important milestone. It is an opportunity for reflection and self-appreciation and for moving on as a stronger, more self-aware person. Marking the end of treatment is also an opportunity to make sure that any shame and feelings of self-stigma that may have surfaced during the treatment phase are dealt with.
2. Invite participants to close their eyes and take some time to consider their journey with TB and DR-TB. Speak slowly, with spaces between sentences for people to really reflect on their own experience.
3. Say something like: "Starting with before you were diagnosed. Allow yourselves to re-experience what it was like to have symptoms, coughing, general feelings of being unwell, the fear and frustration with that. Back to when you were trying to get a diagnosis in a health center or hospital. The feelings that arose at that time. Your experiences in telling (or not telling) family and friends. The early days of starting TB treatment. Then the long phase of taking treatment and how that affected you physically or emotionally. Your visits to the health center during that time to today, the day where you have come out the other side and TB is gone from your body. The day when you no longer must take any medication for TB (this could be especially important for people following the extended treatment period required for drug-resistant TB). Take a little time to reflect on all the people you have met during your TB journey. The other people with TB, the nurses, doctors, any NGO workers, TB activists and advocates."
4. [If you are in an environment where people can go outside to find natural objects, allow them to do so. If not, you will have to use the materials you have collected]. Invite participants to go either outside or to visit the table of objects and to select one object that represents TB.
5. Form groups, and invite each participant to share what object they chose and why it represents TB to them.
6. Placing the object in front of them, every participant should have a pen, paper and a selection of colored markers available.
7. Invite all participants to write a letter to TB using the following format:

   Dear TB,
   The three most difficult things about having TB for you in your life.
   The three most important lessons you have learned from having TB.
   How TB has changed/influenced you positively as a person.
   What commitment can you make to yourself as a TB survivor?
   What commitment can you make to the world as a TB survivor?
   Thank yourself and thank TB.
   Love, XXXX

   The letter can be decorate with colors, symbols, or cartoons. Since this is a reflective exercise, it is important to ensure that adequate time is given for participants to think deeply and write as fully as they would like.

8. Invite volunteers to share their letters one by one. Facilitator should not remark on the letters. No one should be forced to read their letter. If time is short, invite a few volunteers to read to the group and then ask participants to turn to a partner and take turns reading their letters. Those listening should merely thank the reader. When both partners have read their letter, fall into silence.

Exercise 7.2 Accompaniment: being a TB Champion

OBJECTIVE

This exercise supports participants in exploring possibilities to accompany others as a pathfinder or wise companion to help them navigate the pitfalls of TB treatment. Standing up to give testimony can inspire others as well as help you to reframe your experience in a way that fosters resilience and strengthens advocacy.

Flip charts

60 mins.

Individual/group work, personal planning and general feedback.

FACILITATORS STEP-BY-STEP INSTRUCTIONS

Empowerment of people with TB and TB survivors is key for personal development. Advocacy in the context of TB aims for change of attitudes, actions, or even policies and laws. There are many ways a TB survivor can become involved in advocacy and become a TB Champion. They may work with friends, in the community, in networks, or even at the national level helping to shape policy and reduce stigma.

It is important that participants are not pressured during this exercise. Facilitators should emphasize that advocacy can occur at many levels. It can be in a conversation with a friend, or simply being careful in the language used to describe people affected by TB. It's about considering being a role model and reflecting on personal experience to increase empathy with other affected people.

Step 1: Reflection on the letter in Exercise 7.1 [individual]

- Ask people to revisit the letter they wrote for Exercise 7.1 and ask themselves:
    - What are the ways I could help prevent the stigmatization of people being treated for TB?
    - What are the ways I could support people being treated for TB to help reduce their experience of self-stigma, shame, or guilt?
    - How would I achieve my aims and goals?

Step 2: Small group discussion

- Break the participants into groups of two or three.
- Ask them to discuss their thoughts about levels of involvement and pool the suggestions into a list of activities and ways that TB survivors could have a positive influence on the lives of TB patients and on what types of support minimize self-stigma.

Step 3: Feedback and conclusions

- Ask each group to give feedback to the larger group
- Finally, ask individuals before they leave the session to set three personal goals for their own contribution to being a TB Champion (at whatever level feels comfortable for the participant).

Possible examples of areas of involvement or contribution

- Thinking about the words used when talking about people with TB.
- Talking openly about TB (not being afraid of the topic).
- Challenging stigma (speaking out and naming the problem).
- Encouraging others to talk about TB and their experiences of self-stigma and sharing how we confront our feelings of shame and guilt.
- Joining a TB Club and being willing to share experiences to encourage others with TB.
- Encouraging people to use available services.
- Visit and support TB patients and families in the neighborhood and watch for signs of self-stigma. Share experiences.
- Keep revisiting the letter from Exercise 7.1.

Part III - Session wrap

- Revisit the points of learning from the session. Go over each key point in turn, and make sure that the participants have achieved their goals.
- Confirm that participants are clear about issues surrounding life after TB.

MONITORING & EVALUATION **OF THIS INTERVENTION**

MODULE 8

This module will guide facilitators and implementers through the important aspects of monitoring and evaluation of this self-stigma intervention. The module describes the importance and purpose of monitoring and evaluation, provides tools to assist in that task, and provides insight into the interpretation and utilization of these findings.

Monitoring & evaluation of this intervention

Timeline:

- Participant baseline – 30 mins.
- Participant end-line – 50 mins.
- Facilitator self-reflection – 20 mins.

Required materials for this module:

Paper or electronic survey and data entry screens.

Self-administered survey or/ low-literacy alternatives.

Introduction

Objectives of the module

1. To gain skills in monitoring participants' feedback on the organization, the environment (venue), the facilitators, and workshop content.
2. To gain skills in measuring changes in participants' self-stigma levels, perceived self-efficacy, resilience, and psychological well-being.
3. To explain how to adjust tools for low-literacy audiences.

Who will benefit?

This module is aimed at implementers and facilitators to assist them in monitoring and evaluating this self-stigma intervention, and adapting the intervention as necessary.

Summary of this module

This module addresses baseline measurements to map the "stigma situation" prior to the intervention and facilitate the measurement of change (end-line), as well as fine tuning of the intervention design (i.e., selection of appropriate exercises addressing issues flagged, and allocation of additional time to critical issues). The module addresses the monitoring of the intervention to facilitate self-evaluation and continuous improvement of content and delivery.

Advance preparation

Before the workshop, adapt, translate, and pilot all the tools, and revise accordingly. Particular attention should be paid to keeping the core meaning of each question during the translation and adaptation process. In addition, the target audience should be considered – difficulties with literacy or understanding the questions should be anticipated and addressed by facilitators. Participant fatigue may also be a risk, and some participants may have impaired concentration levels due to medication or other factors. For this reason, time of question administration needs to be carefully chosen and implementers and facilitators should aim to use the minimal set of questions for their specific audience.

8.1 Evaluating the effectiveness of this intervention

To evaluate the effectiveness of the intervention, you must assess changes in self-stigma among participants. Since we all start with varying levels of self-stigma and self-compassion, we are largely interested in the difference between before the intervention and after.

Conducting a baseline and end-line with participants:

We recommend you measure the following both before the intervention starts and *again within two months after the intervention is complete*:

1. Participants' self-stigma levels.
2. Participants self-compassion levels.[54]
3. Participant's knowledge of self-stigma and perceived self-efficacy.
4. Participant's psychological well-being.
5. Participant's sociodemographic and clinical information.

8.1.1 Self-stigma levels

TB self-stigma is dynamic during the course of treatment, so the self-stigma metric should be appropriate to the person's stage

of treatment. People who were successfully treated with TB in the past should be asked items that refer to TB disease as in the past.

The Van Rie scale is a validated scale for measuring self-stigma among people with TB.[6] It is one of a small number of scales that has been evaluated for content validity, construct validity, and reliability in multiple settings using rigorous methods.[55-58] It has two items on guilt and three items on anticipated stigma, which could be expected to improve as a consequence of this intervention.

Alternatively, you may use the items from exercise 1.2, which were derived from Stevelink et al. self-stigma domains[5] and adapted from the following validated scales:

1. The six-item self-stigma sub-scale of the Diabetes Stigma Assessment Scale (DSAS-2) adapted for TB.[59]
2. Negative Self-Image subscale of the HIV Stigma Scale.[60]
3. Shame sub-scale for lung cancer stigma.[61]
4. Van Rie[6]

These items are narrowly mapped to this intervention. The combined use of these items has yet to be validated, so a more conservative approach would be to use Van Rie, which is well validated.

The Van Rie Patient Perspectives Towards Tuberculosis scale[6]

The Van Rie scales were developed to measure stigma and self-stigma in HIV and TB. Four parts were created: Community Perspectives Towards HIV/AIDS; Patient Perspectives Towards HIV/AIDS; Community Perspectives Towards Tuberculosis; and Patient Perspectives Towards Tuberculosis.

The community perspectives questions can be used to measure stigma held by general communities, or specific groups such as healthcare providers. The patient perspectives questions are useful in measuring self-stigma held by individuals. Van Rie's scales were first developed in 2007-8, using input from tuberculosis patients in Thailand, combined with input from Thai and American research teams. Since their development, they have been used in many other countries and contexts. The Patient Perspectives Towards Tuberculosis scale is reproduced below (Table 4). Note that the wording of the original questions is in the third-person – i.e. "some people", rather than "I". Higher scores represent higher level of self-stigma.

Question	Strongly disagree	Disagree	Slightly disagree	Not sure	Slightly agree	Agree	Strongly agree	Summary Score
Some people who have TB feel hurt by how others react to knowing they have TB	1	2	3	4	5	6	7	
Some people who have TB lose friends when they share with them they have TB	1	2	3	4	5	6	7	
Some people who have TB feel alone	1	2	3	4	5	6	7	
Some people who have TB keep their distance from others to avoid spreading TB germs	1	2	3	4	5	6	7	
Some people who have TB are afraid to tell those outside their family that they have TB	1	2	3	4	5	6	7	
Some people who have TB are afraid of going to TB clinics because other people may see them there	1	2	3	4	5	6	7	
Some people who have TB are afraid to tell others that they have TB because others may think that they also have AIDS	1	2	3	4	5	6	7	
Some people who have TB feel guilty because their family has the burden of caring for them	1	2	3	4	5	6	7	
Some people who have TB will choose carefully who they tell about having TB	1	2	3	4	5	6	7	

Table 4. Van Rie patient perspective towards tuberculosis scale - Adapted from van Rie, et al.[6]

Some people who have TB feel guilty for getting TB because of their smoking, drinking, or other careless behaviours	1	2	3	4	5	6	7	
Some people who have TB are worried about having AIDS	1	2	3	4	5	6	7	
Some people who have TB are afraid to tell their family that they have TB	1	2	3	4	5	6	7	

Self-stigma is influenced by whether TB is infectious, drug-susceptible or drug resistant, and common. Self-stigma is also influenced by beliefs about how TB was acquired (i.e., through non-adherence). Therefore, the evaluation metric should be linked to the type of TB (DS or DR). As of 2017, there were no validated DR-TB self-stigma measures.

8.1.2 Self-compassion levels

Self-compassion is measured with the Neff scale.[54] The shorter version is 12 questions (see below) and the longer version is 24 items. It is available in 20 languages, and is located here: http://self-compassion.org/self-compassion-scales-for-researchers/

**How I typically act towards myself in difficult times**<br>Instructions: Please read each statement carefully before answering. Indicate how often you behave in the stated manner.	**Strongly disagree**	**Disagree**	**Slightly disagree**	**Not sure**	**Slightly agree**	**Agree**	**Strongly agree**	**Summary Score**
1. When I fail at something important to me, I become consumed by feelings of inadequacy.	1	2	3	4	5	6	7	
2. I try to be understanding and patient towards those aspects of my personality I don't like.	1	2	3	4	5	6	7	
3. When something painful happens I try to take a balanced view of the situation.	1	2	3	4	5	6	7	
4. When I'm feeling down, I tend to feel like most other people are probably happier than I am.	1	2	3	4	5	6	7	
5. I try to see my failings as part of the human condition.	1	2	3	4	5	6	7	
6. When I'm going through a very hard time, I give myself the caring and tenderness I need.	1	2	3	4	5	6	7	
7. When something upsets me, I try to keep my emotions in balance.	1	2	3	4	5	6	7	
8. When I fail at something that's important to me, I tend to feel alone in my failure.	1	2	3	4	5	6	7	
9. When I'm feeling down I tend to obsess and fixate on everything that's wrong.	1	2	3	4	5	6	7	
10. When I feel inadequate in some way, I try to remind myself that feelings of inadequacy are shared by most people.	1	2	3	4	5	6	7	
11. I'm disapproving and judgmental about my own flaws and inadequacies.	1	2	3	4	5	6	7	
12. I'm intolerant and impatient towards those aspects of my personality I don't like.	1	2	3	4	5	6	7	

Table 5. Neff's self-compassion scale - short form (SCS-SF)

8.1.3 Self-stigma knowledge and perceived self-efficacy of the participants

This toolkit is designed to help individuals identify, understand, and address self-stigma. In evaluating knowledge gained, it is also important to add items that can assess the intention to use that knowledge.

Illustrative items for perceived self-efficacy:

- I know what self-stigma is
- I can give examples of the manifestations of self-stigma
- I know the negative effects of self-stigma
- I can give examples of the effects of self-stigma
- I am equipped with techniques to overcome self-stigma
- I can give examples of techniques to overcome self-stigma
- I have tools to overcome self-stigma in myself
- I can assist other people who are experiencing self-stigma

8.1.4 Psychological well-being: The Ryff Dimensions of psychological well-being

The Ryff Dimensions should be used to gain insight into life impacts of the program. This is useful in determining the effectiveness of holistic programs that are aimed at empowering people living with self- develop their capacity to live well. The Ryff Dimensions were chosen because manifestations of self-stigma such as guilt, shame, blame, and stereotype endorsement all affect psychological well-being. Effects of self-stigma, such as social withdrawal, avoidance of seeking care, and fears around disclosure, are also linked to psychological well-being.

The Ryff Dimensions, developed in 1995, provide a measure of how well an individual is functioning across six areas of psychological well-being: purpose in life; environmental mastery; positive relationships with others; personal growth; autonomy; and self-acceptance. The Ryff Dimensions are reproduced below, adapted into plain language. This scale contains reverse-coded items shaded and marked with an asterisk*. Reverse these after questionnaire completion. Average scores for each of the six dimensions should then be calculated. A higher score represents better function. The plain language version has not yet been validated.

ADAPTED RYFF STATEMENT	Strongly disagree	Disagree	Slightly disagree	Not sure	Slightly agree	Agree	Strongly agree	Summary Score
DIMENSION 1: AUTONOMY								
I am not afraid to say what I think, even if it is different from what others think.	1	2	3	4	5	6	7	
My decisions are not usually influenced by other people.	1	2	3	4	5	6	7	
*I worry about what other people think of me.	1	2	3	4	5	6	7	
*I am often influenced by people who have strong opinions.	1	2	3	4	5	6	7	
I have confidence in my opinions, even if they are different from everybody else's.	1	2	3	4	5	6	7	
*It is difficult for me to give my opinion on controversial subjects.	1	2	3	4	5	6	7	
I judge myself by what I think is important, not what other people think is important.	1	2	3	4	5	6	7	
DIMENSION 2: ENVIRONMENTAL MASTERY								
I feel like I am in control of my life.	1	2	3	4	5	6	7	

Table 6. Ryff Dimensions - plain language adapted

ADAPTED RYFF STATEMENT	Strongly disagree	Disagree	Slightly disagree	Not sure	Slightly agree	Agree	Strongly agree	Summary Score
*Everyday life often makes me feel sad.	1	2	3	4	5	6	7	
*I do not fit in very well with the people and the community around me.	1	2	3	4	5	6	7	
I am good at managing the responsibilities in my daily life.	1	2	3	4	5	6	7	
*I often feel like I cannot cope with my responsibilities.	1	2	3	4	5	6	7	
*It is hard for me to live in a way that I find satisfying.	1	2	3	4	5	6	7	
I like my home and my life.	1	2	3	4	5	6	7	
DIMENSION 3: PERSONAL GROWTH								
*I am not interested in doing new activities.	1	2	3	4	5	6	7	
It is important to have new experiences that change the way I think about myself and the world around me.	1	2	3	4	5	6	7	
*I do not think I have improved as a person over time.	1	2	3	4	5	6	7	
I think that I have developed a lot as a person over time.	1	2	3	4	5	6	7	
*I do not like being in new situations where I have to change my way of doing things.	1	2	3	4	5	6	7	
My life has been a continuous process of learning, changing, and growth.	1	2	3	4	5	6	7	
*I gave up trying to make big improvements or changes to my life a long time ago.	1	2	3	4	5	6	7	
DIMENSION 4: POSITIVE RELATIONSHIPS WITH OTHERS								
Most people think I am a loving and affectionate person.	1	2	3	4	5	6	7	
*It has been difficult and frustrating for me to maintain close relationships with other people.	1	2	3	4	5	6	7	
*I often feel lonely because I do not have many close friends to share my worries with.	1	2	3	4	5	6	7	
I enjoy talking closely with family and friends.	1	2	3	4	5	6	7	
Other people think I am generous, and that I am willing to share my time with them.	1	2	3	4	5	6	7	
*I have not experienced many good relationships with other people.	1	2	3	4	5	6	7	
I know that I can trust my friends, and they know they can trust me.	1	2	3	4	5	6	7	
DIMENSION 5: PURPOSE IN LIFE								
*I live life one day at a time, and don’t really think about the future.	1	2	3	4	5	6	7	
I have a purpose in life.	1	2	3	4	5	6	7	
*I often feel like my daily activities are meaningless.	1	2	3	4	5	6	7	
*I do not know what I want to accomplish in my life.	1	2	3	4	5	6	7	

ADAPTED RYFF STATEMENT	Strongly disagree	Disagree	Slightly disagree	Not sure	Slightly agree	Agree	Strongly agree	Summary Score
I enjoy making plans for the future and working to make them happen.	1	2	3	4	5	6	7	
Some people do not have goals in their lives, but I am not like that.	1	2	3	4	5	6	7	
*I sometimes feel as if I have done everything that can be done in life.	1	2	3	4	5	6	7	
DIMENSION 6: SELF-ACCEPTANCE								
I am pleased with how my life is.	1	2	3	4	5	6	7	
In general, I feel confident and positive about myself.	1	2	3	4	5	6	7	
*I feel like many of the people I know have better lives than me.	1	2	3	4	5	6	7	
I like my personality.	1	2	3	4	5	6	7	
*I feel disappointed about my achievements in life.	1	2	3	4	5	6	7	
*I feel less positive about myself than other people feel about themselves.	1	2	3	4	5	6	7	
When I compare myself to the people I know, it makes me feel good about who I am.	1	2	3	4	5	6	7	

8.1.5 Sociodemographic and clinical information

Sociodemographic and clinical information provides necessary context and background information for proper evaluation. Information under the sociodemographic heading includes: age, gender, education, migration background, ethnicity, religious affiliation, marital status, household information, employment, and income. Different index variables are based on socio-demographic variables, such as socio-economic status, which combines information on education and income. Clinical information includes TB type, location of infection (pulmonary, extra-pulmonary, etc.), drug-resistance status, and treatment history.

This information is used to describe the social context of the participants, and to determine if there might be confounders or a correlation between the described self-stigma levels and other characteristics shared by the participants, beyond their TB diagnosis (e.g., gender, marital status, employment status, etc.)

How to design a baseline survey

Guidance on how to design a stigma measurement survey instrument is found here: www.challengetb.org/publications/tools/ua/TB_Stigma_Measurement_Guidance.pdf

Most scales use the Likert system, where 1 is assigned to the negative response and 7 is assigned to the affirmative response.

- Strongly disagree = 1
- Disagree =2
- Somewhat disagree = 3
- Not sure = 4
- Somewhat agree = 5
- Agree = 6
- Strongly agree = 7

The scores are then added and compared across multiple variables, such as gender, location, age, treatment duration, health care provider, etc. Seven -point Likert scales are also preferred by respondents. Moreover they are

sufficiently responsive to detect subtle improvements. An example baseline end-line survey is attached in Annex 15.

Translation, cultural adaptation, and piloting of validated scales

Before administering any tools, a local team should first pilot the questions to make sure that the intended audience will understand them. If the wording needs to be changed, make sure that the meaning of each question is still the same. Questions should be culturally appropriate as well (for example, is it more culturally acceptable to ask the question in third-person, or first-person?)

How to administer a baseline survey

1. Someone who is familiar with all the questions and the scoring system should explain how to self-administer the survey. Ensure privacy. Use a unique ID instead of names whenever possible.
2. In the case of low-literacy participants, a person can administer the questionnaire by reading each question aloud. Make sure the participant understands the question.
3. Note that a proper baseline including confounders and appropriate scales will be long, and it can be broken up into two or more sessions. This helps avoid "question fatigue" – i.e., the recipient gets bored and simply answers "agree" for every question.

How to analyze the data and interpret the results

Pay attention to any positively phrased questions to ensure you recode them correctly (e.g., if someone responds "strongly agree" to the statement "TB patients are just like everyone else," that would need to be reverse coded).

Formal Evaluation

If a rigorous evaluation of effectiveness is required, then we recommend that you capture the following participant characteristics on (or before) the first day of the intervention for all participants. These metrics are used to gather information on the individuals' context, especially those factors that can impact the effectiveness of the intervention.

1. TB treatment history and comorbidities.
2. Experiences of everyday discrimination index (EODI).
3. Perceptions of respectful TB care or discrimination.[62]
4. Prior exposure(s) to related material (e.g., self-esteem courses, empowerment interventions, resilience).
5. Concurrent interventions (e.g., social and psychological support).

Measuring related stigmas

Finally, depending on the other stigmatized identities participants are managing, you may also consider including the following in the baseline:

1. Substance Abuse Self-Stigma Scale.[63]
2. Self-Stigma of Individuals with Criminal Records Scale (SSICR).[64]
3. Internalized Stigma of Smoking Inventory (ISSI).[65]
4. Diabetes Self-Stigma Scale (SSS).[66, 67]
5. Internalized HIV self-stigma scale.[60]

For more information on evaluating self-stigma and its wider impacts, please see Chapter 10 in "Measuring self-stigma, resilience, resistance, and deflection of TB stigma" in the Measuring TB Stigma Guidance and the Chapter 10 Companion Curriculum "Assessing, understanding and measuring TB self-stigma in context: an agenda for change". www.challengetb.org/publications/tools/ua/TB_Stigma_Measurement_Guidance.pdf

Chapter 10 gives readers insight into the key features of self-stigma and its manifestations. It also discusses the challenges in defining self-stigma, and highlights some key methods to investigate its effects. The chapter draws on international evidence from TB, HIV, and mental illness. The companion curriculum then provides learners with examples, tools, and exercises to help them explore the multi-faceted nature of self-stigma.

8.2 Monitoring implementation of the intervention

It is important to monitor workshop implementation. This should be done in two ways:

1. Monitoring the number, type, and way exercises were included via the facilitator pilot feedback form (below).
2. Monitoring participants' feedback on the facilitator, content, organization, and venue via the participant feedback form.

Facilitator pilot feedback form

This form should be completed by facilitators as a self-reflection to provide useful information for future adjustments to the package.

1. Which exercises did you use?
2. What kind of training did you use the exercises for, and who was the target audience?
3. Which exercises worked well? Why?
4. Which exercises did not work well? Why?
5. Please describe any changes or improvements you made to any exercises.
6. What additional topics need to be added to the toolkit?
7. Have you developed any case studies or other materials on stigma? If yes, please describe. Send them to KNCV, if possible.
8. Please provide your name and address so we can follow up with you if necessary.

Feedback forms should be reviewed after each workshop to ensure that program improvements can be made in future. Illustrative questions are given below. Responses can be recorded using a four-point Likert-type scale of: 1= strongly disagree; 2= slightly disagree; 3= slightly agree; 4= strongly agree; or as free text response where appropriate. Facilitators can also provide information on how to improve the workshops and the intervention toolkit overall.

Illustrative items for self-evaluation by facilitators include:

- I had sufficient knowledge to answer any questions from the participants (Likert scale AND free text response).
- I was able to run the workshop with good timing (Likert scale AND free text response).
- I made sure that the participants felt welcome and comfortable while participating in the workshop (Likert scale AND free text response).
- I made sure that all participants were included during discussions and exercises (Likert scale AND free text response).

An example facilitator self-evaluation form can be found in Annex 16.

Monitoring participant feedback

Gathering participant feedback on the workshops will reveal any technical, personal, organizational, or environmental challenges that can be addressed. Participants should be invited to share their ideas about the organization, venue, facilitators, and content.

Sample statements for gathering feedback on satisfaction with the organization:

- We had enough time to complete the exercises during the workshop (Likert scale).
- Our needs were met during the workshop (Likert scale).

Sample items for satisfaction with the (venue:

- The workshop venue was comfortable.
- The air was safe (Likert scale AND free text response).
- The workshop venue had enough sound and visual privacy (Likert scale AND free text response).
- The workshop venue was easily accessible (e.g., for participants with disabilities) (Likert scale AND free text response).

Facilitator feedback can be provided by participants, peers, supervisors, or facilitators. The aim of this feedback should be to help the facilitator understand their strengths and weaknesses and find areas where they can improve. Ultimately, monitoring tools should focus on gathering information, complements, and tips to help the facilitators become as capable and as knowledgeable as possible.

Sample statements for evaluating satisfaction with facilitation:

- Facilitator A was able to deal well with emotions.
- Facilitator A is a good listener and refrained from giving advice.
- Facilitator A can connect well with people.
- Facilitator A can work well cross-culturally.
- Facilitator A has good organizational skills.
- Facilitator A shows self-compassion.
- Facilitator A did not try to fix me.
- Facilitator A was knowledgeable and answered questions satisfactorily (Likert scale).
- Facilitator A was approachable and friendly (Likert scale).
- Facilitator A explained the exercises and information satisfactorily (Likert scale).
- Facilitator A kept good time and ensured that the workshop ran smoothly (Likert scale).

Illustrative items for satisfaction with content

- Which exercises worked well? Why? [Free text response]
- Which exercises did not work well? Why? [Free text response]
- The workshop delivered useful information and skills to help address self-stigma (Likert scale).

If you need more detailed insight, ask participants to rate each exercise.

An example participant evaluation form can be found in Annex 17.

References

1. France, N.F., et al., “An unspoken world of unspoken things”: a study identifying and exploring core beliefs underlying self-stigma among people living with HIV and AIDS in Ireland. Swiss Med Wkly, 2015. 145: p. w14113.
2. Morrison, K., Breaking the cycle : stigma, discrimination, internal stigma, and HIV. 2006, USAID, Futures Group, POLICY Project: Washington, DC. p. 1-15.
3. Kalichman, S.C., et al., Measuring AIDS stigmas in people living with HIV/AIDS: the Internalized AIDS-Related Stigma Scale. AIDS Care, 2009. 21(1): p. 87-93.
4. Corrigan, P.W., J.E. Larson, and N. Rusch, Self-stigma and the “why try” effect: impact on life goals and evidence-based practices. World Psychiatry, 2009. 8(2): p. 75-81.
5. Stevelink, S.A., W.H. van Brakel, and V. Augustine, Stigma and social participation in Southern India: differences and commonalities among persons affected by leprosy and persons living with HIV/AIDS. Psychol Health Med, 2011. 16(6): p. 695-707.
6. Van Rie, A., et al., Measuring stigma associated with tuberculosis and HIV/AIDS in southern Thailand: exploratory and confirmatory factor analyses of two new scales. Trop Med Int Health, 2008. 13(1): p. 21-30.
7. Ryff, C.D. and C.L. Keyes, The structure of psychological well-being revisited. J Pers Soc Psychol, 1995. 69(4): p. 719-27.
8. Cameron, J.E., Internal Stigma and AIDS, in 17th National Congress of South African Society of Psychiatrists (SASOP). 2012: Johannesburg, South Africa.
9. Parker, R. and P. Aggeleton, HIV / AIDS-related Stigma and Discrimination : A Conceptual Framework and an Agenda for Action. Social Science & Medicine, 2003. 57: p. 1-28.
10. Castro, A. and P. Farmer, Understanding and addressing AIDS-related stigma: from anthropological theory to clinical practice in Haiti. Am J Public Health, 2005. 95(1): p. 53-9.
11. Deacon, H., Towards a sustainable theory of health-related stigma: lessons from the HIV/AIDS literature. Journal of Community and Applied Social Psychology, 2006. 16(6): p. 418-425.
12. Ryff, C.D., Psychological well-being revisited: advances in the science and practice of eudaimonia. Psychother Psychosom, 2014. 83(1): p. 10-28.
13. Brohan, E., et al., Self-stigma, empowerment and perceived discrimination among people with schizophrenia in 14 European countries: the GAMIAN-Europe study. Schizophr Res, 2010. 122(1-3): p. 232-8.
14. Puhl, R.M., M.S. Himmelstein, and D.M. Quinn, Internalizing Weight Stigma: Prevalence and Sociodemographic Considerations in US Adults. Obesity (Silver Spring), 2018. 26(1): p. 167-175.
15. Macq, J., et al., Tackling tuberculosis patients’ internalized social stigma through patient centred care: an intervention study in rural Nicaragua. BMC Public Health, 2008. 8: p. 154.
16. South African National AIDS Council, The People Living With HIV Stigma Index: South Africa 2014 – Summary Report. 2015, South African National AIDS Council.
17. Young, D.K. and P.Y. Ng, The prevalence and predictors of self-stigma of individuals with mental health illness in two Chinese cities. Int J Soc Psychiatry, 2016. 62(2): p. 176-85.
18. Oduguwa, T.O., O.O. Akinwotu, and A.A. Adeoye, A comparative study of self stigma between HIV/AIDS and schizophrenia patients. Vol. 17. 2014. 525-531.
19. Global Network of People living with HIV and AIDS (GNP+), People living with HIV Stigma Index: Asia Pacific Regional Analysis. 2011, UNAIDS.
20. Pennebaker, J.W., Writing to heal: a guided journal for recovering from trauma and emotional upheaval. 2004.
21. International HIV/AIDS Alliance, Understanding and challenging HIV stigma. Toolkit for action - Module F: Coping with Stigma. Understanding and challenging HIV stigma. Toolkit for action, ed. R. Kidd, S. Clay, and C. Chiiya. 2007: International HIV/AIDS Alliance.
22. STAMPP, CREATE, and International HIV/AIDS Alliance, Understanding and challenging TB stigma - Toolkit for action. 2009: ZAMBART Project, International HIV/AIDS Alliance.

23. de Bruyn, M. and N.F. France, Gender or sex: who cares? Skills-building resource pack on gender and reproductive health for adolescents and youth workers. 2001, Chapel Hill: Ipas & Health & Development Networks (HDN).

24. Compton, J., B. Jackson, and J.A. Dimmock, Persuading Others to Avoid Persuasion: Inoculation Theory and Resistant Health Attitudes. Front Psychol, 2016. 7: p. 122.

25. WHO, Global Tuberculosis Report 2016. 2016, WHO: Geneva. p. 130.

26. Kanabus, A. Drug resistant TB – Acquired, primary, types, statistics, global. Information about Tuberculosis 2017 [cited 2018; Available from: https://www.tbfacts.org/drug-resistant-tb/.

27. Shin, S., et al., Community-based treatment of multidrug-resistant tuberculosis in Lima, Peru: 7 years of experience. Soc Sci Med, 2004. 59(7): p. 1529-39.

28. Morris, M.D., et al., Social, economic, and psychological impacts of MDR-TB treatment in Tijuana, Mexico: a patient's perspective. Int J Tuberc Lung Dis, 2013. 17(7): p. 954-60.

29. CDC. TB Elimination: Multidrug resistant Tuberculosis (MDR-TB). 2016 [cited 2018; Available from: www.cdc.gov/tb/publications/factsheets/drtb/mdrtb.htm.

30. Stringer, B., 'They prefer hidden treatment': anti-tuberculosis drug-taking practices and drug regulation in Karakalpakstan. Int J Tuberc Lung Dis, 2016. 20: p. 1084-1090.

31. Elsey, H. and I. Walker. The link between multi-drug resistant TB and mental health. 2017; Available from: http://www.stoptb.org/webadmin/cms/docs/Blog%20WITH%20NEW%20PIC.pdf.

32. Keshavjee, S. and P.E. Farmer, Time to put boots on the ground: making universal access to MDR-TB treatment a reality. Int J Tuberc Lung Dis, 2010. 14(10): p. 1222-5.

33. Isaakidis, P., et al., 'I cry every day': experiences of patients co-infected with HIV and multidrug-resistant tuberculosis. Trop Med Int Health, 2013. 18(9): p. 1128-1133.

34. Yang, T.W., et al., Side effects associated with the treatment of multidrug-resistant tuberculosis at a tuberculosis referral hospital in South Korea: A retrospective study. Medicine (Baltimore), 2017. 96(28): p. e7482.

35. Lange, C., et al., Management of patients with multidrug-resistant/extensively drug-resistant tuberculosis in Europe: a TBNET consensus statement. Eur Respir J, 2014. 44(1): p. 23-63.

36. Skrahina, A., et al., Alarming levels of drug-resistant tuberculosis in Belarus: results of a survey in Minsk. Eur Respir J, 2012. 39(6): p. 1425-31.

37. Kanabus, A. TB Prevention – Precautions, vaccine, masks. Information about Tuberculosis 2017 [cited 2018; Available from: https://www.tbfacts.org/tb-prevention/.

38. WHO, WHO Policy on TB Infection Control in Health-Care Facilities, Congregate Settings and Households. 2009: Geneva.

39. Kleinman, A., Patients and healers in the context of culture: an exploration of the borderland between anthropology, medicine, and psychiatry. 1980, Berkley: University of California Press.

40. Gebremariam, A. and A. Addissie, Knowledge and perception on long acting and permanent contraceptive methods in adigrat town, tigray, northern ethiopia: a qualitative study. Int J Family Med, 2014. 2014: p. 878639.

41. West, E.L., et al., Tuberculosis knowledge, attitudes, and beliefs among North Carolinians at increased risk of infection. N C Med J, 2008. 69(1): p. 14-20.

42. Edginton, M.E., C.S. Sekatane, and S. Goldstein, Patients' beliefs: Do they affect tuberculosis control? A study in a rural district of South Africa. Int J Tuberc Lung Dis, 2003. 6(12): p. 1075-82.

43. Viney, K.A., et al., Tuberculosis patients' knowledge and beliefs about tuberculosis: a mixed methods study from the Pacific Island nation of Vanuatu. BMC Public Health, 2014. 14: p. 467.

44. Paz-Soldan, V.A., et al., The provision of and need for social support among adult and pediatric patients with tuberculosis in Lima, Peru: a qualitative study. BMC Health Serv Res, 2013. 13: p. 290.

45. Baral, S.C., D.K. Karki, and J.N. Newell, Causes of stigma and discrimination associated with tuberculosis in Nepal: A qualitative study. BMC Public Health, 2007. 7.

46. Simbayi, L.C., et al., Internalized stigma, discrimination, and depression among men and women living with HIV/AIDS in Cape Town, South Africa. Soc Sci Med, 2007. 64(9): p. 1823-31.

47. UN Committee on Economic, Social and Cultural Rights (CESCR) (2009), General comment No. 20: Non-discrimination in economic, social and cultural rights (art. 2, para. 2, of the International Covenant on Economic, Social and Cultural Rights), 2 July 2009, E/C.12/GC/20, available at: http://www.refworld.org/docid/4a60961f2.html [accessed 4 October 2018]

48. World Health Organization, Ethics guidance for the implementation of the End TB Strategy. 2017, Geneva: WHO.

49. UNDP and Stop TB Partnership, Legal Environment Assessments For Tuberculosis: An Operational Guide. 2017.

50. CDC. Treatment for TB disease. 2018 [cited 2018; Available from: https://www.cdc.gov/tb/topic/treatment/tbdisease.htm.

51. Munro, S.A., et al., Patient Adherence to Tuberculosis Treatment: A Systematic Review of Qualitative Research. PLoS Medicine, 2007. 4: p. e238.

52. Hodgson, I., et al., Only connect--the role of PLHIV group networks in increasing the effectiveness of Ugandan HIV services. AIDS Care, 2012. 24(11): p. 1368-74.

53. Dias, A.A., et al., Life experiences of patients who have completed tuberculosis treatment: a qualitative investigation in southeast Brazil. BMC Public Health, 2013. 13: p. 595.

54. Neff, K., The development and validation of a scale to measure self-compassion. Self and Identity, 2003. 2: p. 223-250.

55. Kipp, A.M., et al., Re-validation of the Van Rie HIV/AIDS-related stigma scale for use with people living with HIV in the United States. PLoS One, 2015. 10(3): p. e0118836.

56. Moya, E.M., et al., Assessment of stigma associated with tuberculosis in Mexico. Public Health Action, 2014. 4(4): p. 226-232.

57. Almeida, G.P.L., et al., Reliability and Validity of the Hip Stability Isometric Test (HipSIT): A New Method to Assess Hip Posterolateral Muscle Strength. J Orthop Sports Phys Ther, 2017. 47(12): p. 906-913.

58. Hayes-Larson, E., et al., High baseline prevalence of stigma, depressive symptoms and hazardous alcohol use among TB/HIV patients in Lesotho. International Journal of Tuberculosis and Lung Disease, 2017. supplement.

59. Browne, J.L., et al., Measuring the Stigma Surrounding Type 2 Diabetes: Development and Validation of the Type 2 Diabetes Stigma Assessment Scale (DSAS-2). Diabetes Care, 2016. 39(12): p. 2141-2148.

60. Berger, B.E., C.E. Ferrans, and F.R. Lashley, Measuring stigma in people with HIV: psychometric assessment of the HIV stigma scale. Res Nurs Health, 2001. 24(6): p. 518-29.

61. Cataldo, J.K., et al., Measuring stigma in people with lung cancer: psychometric testing of the cataldo lung cancer stigma scale. Oncol Nurs Forum, 2011. 38(1): p. E46-54.

62. Sheferaw, E.D., T.Z. Mengesha, and S.B. Wase, Development of a tool to measure women's perception of respectful maternity care in public health facilities. BMC Pregnancy and Childbirth, 2016. 16: p. 67.

63. Luoma, J.B., et al., Self-Stigma in Substance Abuse: Development of a New Measure. J Psychopathol Behav Assess, 2013. 35(2): p. 223-234.

64. Moore, K.E., J.P. Tangney, and J.B. Stuewig, The Self-Stigma Process in Criminal Offenders. Stigma Health, 2016. 1(3): p. 206-224.

65. Brown-Johnson, C.G., et al., Validity and reliability of the Internalized Stigma of Smoking Inventory: An exploration of shame, isolation, and discrimination in smokers with mental health diagnoses. Am J Addict, 2015. 24(5): p. 410-8.

66. Kato, A., et al., Association between self-stigma and self-care behaviors in patients with type 2 diabetes: a cross-sectional study. BMJ Open Diabetes Res Care, 2016. 4(1): p. e000156.

67. Kato, A., M. Takada, and H. Hashimoto, Reliability and validity of the Japanese version of the self-stigma scale in patients with type 2 diabetes. Health Qual Life Outcomes, 2014. 12: p. 179.

ANNEXES

Annex 1 – Additional resources, interventions and ideas

This Annex includes examples of initiatives to address stigma and self-stigma. They include networking and narrative sharing.

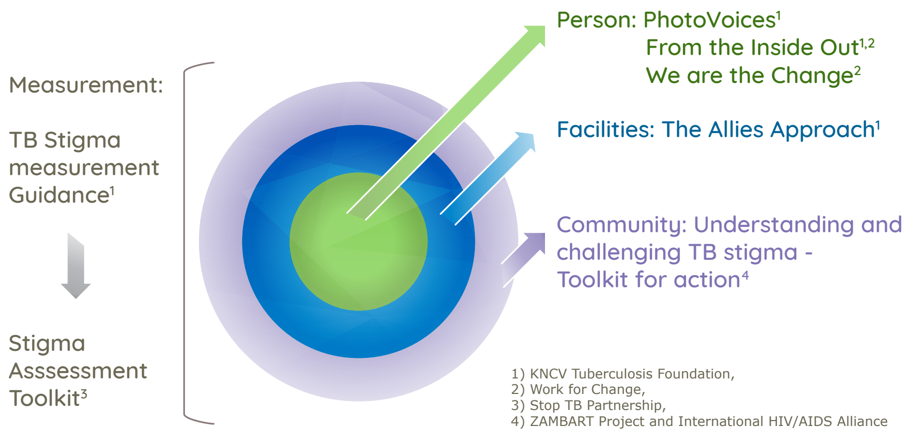

Figure 13. Stigma - spaces and scope of intervention and corresponding tools

> The thing I'm most ashamed of is...
> I infected my daughter with HIV
> I lied to my partner about my status
> I have an ugly body
> And that means that...

From the Inside Out can also be combined with community and facility level interventions to reduce stigma. The Allies Approach, an intervention to reduce TB stigma in health care facilities (end-stigma-in-health-care.com). The intervention has an e-learning course for health care workers, a policy reform component, and group exercises to build capacity in respectful, dignified, and empowering care of people with TB.

Indepth intervention on self-stigma

We are the change: Reducing self-stigma and shame among people living with HIV in Zimbabwe, The Work for Change.

This is a 12-course program on self-stigma, shame, and HIV (currently being adapted to gender-based violence, available to be adapted to support people with TB and TB survivors).*

**Some of the exercises in the current toolkit have been adapted from the Zimbabwe program for use by general facilitators to support people with TB.*

This course is designed to support participants to identify and question all the negative, self-stigmatizing thoughts and beliefs they have living with HIV using a method of self-inquiry called 'The Work': Inquiry-based stress reduction (IBSR). Based on formative research, each session is carefully crafted so that it tackles all aspects of self-stigma and shame to provide a 360 degree, comprehensive, and deeply powerful experience. It goes way beyond HIV and well into self-stigma as it shows up in many parts of a person's life. This is a course that works from the inside out.

'The Work: IBSR' is an evidence-based approach to tackling HIV self-stigma in high and low-income settings. It gives individuals the power to address self-stigma and stigma-related conditions themselves, through a community-based,

peer-to-peer approach. It improves well-being and positively affects self-agency and self-esteem, and was recently included in a UNAIDS publication for innovative community-based responses to HIV.

Participants who took the course consistently report profound shifts in their lives, including around living positively with HIV, increased confidence and self-agency in areas of education and professional development, improved communication and relationships with their families, lessened fears about disclosure, not feeling limited by HIV, and increased peacefulness. Quantitatively, results show statistically significant improvements in several areas (percent improved): self-stigma (61 percent), depression (78 percent), life satisfaction (52 percent), fears around disclosure (52 percent) and daily activity (70 percent).

Delivered by trained Certified Facilitators, the 12-session group course is cumulative and is 60 hours in total. This includes a group session of four hours that is run preferably once a week, and an individual one-hour session with a Certified Facilitator/Coach in the Work of Byron Katie (this can be adapted depending on time and financial resources available).

First piloted in Zimbabwe in 2013 with local people living with HIV trained in the method, it is currently expanding to focus on self-stigma and shame among gender-based violence survivors in Zimbabwe. We are also currently working to develop a course on self-stigma and shame among sex workers in Vietnam.

This whole program was developed in partnerships with a group of non-profit organizations and research institutions, including the Zimbabwe National Network of People Living with HIV (ZNNP+), Trócaire, Ireland, Connect Zimbabwe, Royal College of Surgeons in Ireland, Impact Research International, and Byron Katie International (BKI). Through the process, the Community of the Work in Zimbabwe was formed.

This program is suitable as a follow-up to the toolkit training for those who would like to provide a comprehensive and in-depth course on self-stigma. For more information or to have this program delivered in your country, please contact: nadine@theworkforchange.org.

TB CLUBS

TB Clubs for people being treated for TB empower and enable TB patients to take more responsibility for their health, especially around treatment adherence. They are patient centred, a safe space where patients can share their problems and promote self-help. They also present opportunities for TB survivors (those the cured) to meet with new patients to help motivate the latter to adhere to treatment.

There are many international examples of TB Clubs. In Bangladesh, the clubs form part of a government program run by an NGO. In Mongolia, TB Clubs are organized with cured patients, patients under treatment, and community leaders. In Colombia, they are a component of a community care programs, and in Zambia, former TB patients have created health education committees, which give talks about TB, thereby reducing the stigma linked to the disease. Finally, in Peru, patients in two hospitals and 24 health centers constitute the basis of an NGO.

Evaluations of TB Clubs report significant benefits, such as changes in patients' understanding of TB, patients' initial reaction to a TB diagnosis, misconceptions about the cause and treatment of TB, social isolation, and compliance and belief in modern health care in the TB club area. Community elders, community health agents, and local health workers helped TB clubs in referring suspected cases, promoting treatment adherence, and tracing those lost to follow-up (which is an integral part of a district TB programme).

[Source: WHO (2007). Empowerment and involvement of tuberculosis patients in tuberculosis control: Documented experiences and interventions. Online at: http://www.who.int/tb/publications/who_htm_stb_2007_39/en/]

The Positive Vibes Approach (South Africa)

Positive Vibes (PV) is a capacity-development organization that supports LGBTI individuals and people living with HIV in South Africa.

PV supports people to overcome challenges such as economic marginalisation, a lack of information and resources to fulfil one's basic rights, and stigma and discrimination. One key empowerment tool used by PV is the "Looking In - Looking Out (LILO)" approach. LILO uses a personalized approach to help participants explore gender identity and sexual orientation.

First, they are encouraged to look back on their lives. Then they are encouraged to look at what is happening internally, and to look out at how the world is around them. Lastly, they look forward, which helps them gain confidence and positivity for their future.

LILO workshops encourage individuals to share their own unique experiences and views to help others. By doing so, they unpack feelings, needs, and desires, helping them to make the best choices and plans for themselves.

Another useful tool that PV uses is "Body Mapping". During Body Mapping, participants work with an artist and a counsellor to create a painting of themselves that visually records the impacts of HIV on their body and life. They then share the paintings as a group, each giving a narrative of their personal story and describing what the images in the painting represent. This helps the group explore and understand their unique and shared challenges and develop strategies to address them. PV also gives training workshops that help participants understand and address HIV stigma and discrimination.

For more information, see: http://www.positivevibes.org/what_we_do.html

TB PhotoVoice

TB PhotoVoice is a project designed to give a voice to TB survivors so they can become part of the TB elimination solution. The stories they share, according to the project website, can "expose the harsh realities and teach us how to better prevent, diagnose, and cure TB. TB PhotoVoice programs help bring their voices out into the community to bring about positive change and education".

TB PhotoVoice uses a range of media for TB survivors to tell their stories, such as photographs, videos, and narratives. This details the lived experience of having TB, the treatment experience, and the community response. Through such an approach, we can learn much more about the challenges and personal solutions for how deal with the difficulties of a TB diagnosis and treatment progress.

TB PhotoVoice also provides a rich and highly personal perspective on how best to guide communities in increasing TB awareness and reduce the stigma of people at risk, those being treated for TB, and TB survivors. For one TB survivor from India, sharing her story through TB PhotoVoice:

Every day was a fight, every day I won! Of all my achievements and learning in life, winning over tuberculosis was my best win and the best lesson of life.

Studies suggest that increased empowerment and self-awareness does help reduce self-stigma. This project offers a way for TB survivors to share their experiences and strengthen networks.

For more information, see: http://tbphotovoice.org

Studies suggest that increased empowerment and self-awareness does help reduce self-stigma. This project offers a way for TB survivors to share their experiences and strengthen networks.

For more information: http://tbphotovoice.org and www.kncvtbc.org/stigma

Annex 2 – Sample agendas

Sample agenda 1: Two-day workshop

Day one	Activity	Topic
8:15-8:30 am	Registration	
8:30-9:15 am	Introductions and Expectations	
	Baseline	
9:15-10:45 am	Module 1 [exercises 1.1, 1.2 or 1.3]	What is self-stigma?
10:45-11:00 am	BREAK	
11:00-12:00 pm	Module 2 [exercises 2.1 and 2.2]	Dealing with self-stigma and shame.
12:00-1:15 pm	LUNCH	
1:15-1:45 pm	Module 2 [exercise 2.3]	
1:45-3:15 pm	Module 2 [exercise 2.6]	
3:15-3:30 pm	BREAK	
3:30-4:30 pm	Module 2 [exercise 2.12]	
4:30 pm	Finish	

Day two	Activity	Topic
8:15-8:30 am	Icebreaker/progress review	
8:30-9:15 am	Module 3 [exercise 3.1]	DR-TB
9:15-10:45 am	Module 4 [exercise 4.1]	Transmission control and self-stigma.
10:45-11:00 am	BREAK	
11:00-12:00 pm	Module 4 [exercise 4.2]	
12:00-1:15 pm	LUNCH	
1:15-2:15 pm	Module 6 [exercise 6.1]	Treatment and self-stigma.
2:15-3:15 pm	Module 6 [exercise 6.2]	
3:15-3:30 pm	BREAK	
3:30-4:30 pm	Module 7 [exercise 7.1]	Planning for the future – TB free! What now?
4:30-4:45 pm	Review and concluding comment / Evaluation	
4:45 pm	Finish	

Sample Agenda 2 - Self-Stigma interventions integrated into a regular psychosocial support group

Increasingly, DR-TB programs offer group psychotherapy to DR-TB patients. Such programs have proven effective for increasing well-being, decreasing isolation, and fostering treatment adherence.

Below we offer a sample agenda for how DR-TB self-stigma work can be integrated as part of an existing psychosocial support programs. Exercises in this toolkit can be combined with a peer and clinical support group models to compliment clinical, educational, artistic, and advocacy work.

SESSION	CLINICAL SESSION	SELF-STIGMA
**1.**	Introduction, Welcome participants to the session	Testimony of DR-TB Survivor
**2.**	Group psycho therapy	Baseline, DR-TB Quiz
**3.**	Group psycho therapy	Module 1: What is self-stigma?
**4.**	Coping with DR-TB drug side-effects	Exercise 5.1 Rights and the patient with TB
**5.**		Exercise 1.1 Understanding self-stigma and its effects [30 min]
**6.**	Group psycho therapy	Exercise 1.1b Living with self-stigma [20-30 min]
**7.**	Group psycho therapy	Exercise 1.2 Recognizing TB self-stigma and its challenges [60 min]
**8.**	Group psycho therapy	Exercise 1.3 Recognizing self-stigma in the broader context of well-being [60 min]
**9.**	Group psycho therapy	Module 4: Transmission control and self-stigma
**10.**	Inspirational Video: DR-TB survivor	Module 3: Drug-resistant TB (DR-TB) and self-stigma
**11.**	Group psycho therapy	Module 2: Dealing with self-stigma and shame
**12.**	Group psycho therapy	Testimony of DR-TB Survivor
**13.**	Group psycho therapy	Exercise 2.1 Keeping a TB Journal
**14.**	Group psycho therapy	Exercise 2.2 Being breathed
**15.**	Group psycho therapy	Exercise 2.3 Universality, self-stigma, and shame
**16.**	Group psycho therapy	Exercise 3.1 The many faces of DR-TB
**17.**	Recognizing stigma and discrimination in health care	Exercise 4.2 What do we know about TB and risk?
**18.**	Group psycho therapy	Exercise 2.4 What I think you think about me
**19.**	Photovoices	Exercise 2.5 Who is judging whom?
**20.**	Group psycho therapy	Exercise 2.6 How TB affects me?
**21.**	Group psycho therapy	Exercise 2.7 Do you believe everything you think? I have TB and that means that....
**22.**	Group psycho therapy	Exercise 2.8 Living self-stigma
**23.**	Group psycho therapy	Exercise 2.9 My journey - visualizing TB and stigma
**24.**	Photovoices	Exercise 2.10 My right to tell
**25.**	Group psycho therapy	Exercise 2.11 Staying in your own business: reducing stress
**26.**	Group psycho therapy	Exercise 2.12 My agency, my power
**27.**	Mid-Term Evaluation	

SESSION	CLINICAL SESSION	SELF-STIGMA
**28.**	Group psycho therapy	Exercise 4.1 TB lifeline
**29.**	Photovoices	
**30.**	Group psycho therapy	Exercise 5.2 Perfectly Imperfect
**31.**	Photovoices	Exercise 6.1 TB self-stigma and treatment
**32.**	Group psycho therapy	Exercise 6.2 Treating my body, treating my mind
**33.**	Advocacy testimony	Advocacy skills - for new policies
**34.**	Photovoices	Exercise 7.1 The end of my TB journey - moving on
**35.**	Group psycho therapy	Exercise 7.2 Accompaniment: being a TB Champion
**36.**	Family Day	Graduation Ceremony/Photovoices exhibition
**37.**	End-line evaluation	

Annex 3 – Self-stigma learning tool 1: Defining self-stigma

1) Self-Stigma: what is it?

Self-disabling inner feelings of contamination, self-rejection, and self-loathing...even when there is no objective reason to fear rejection or discrimination, and even when there is good objective reason to believe that they will receive external support, protection, treatment, and acceptance.

Justice Edwin Cameron (South Africa), 2012.

2) What does self-stigma cause?

Not seeking care

Higher levels of depression

Fears around disclosure

Lower quality of life

Low self-esteem

Reduced self-efficacy

Lower treatment adherence

3) Self-stigma is the result of complex interactions between social, contextual and self factors

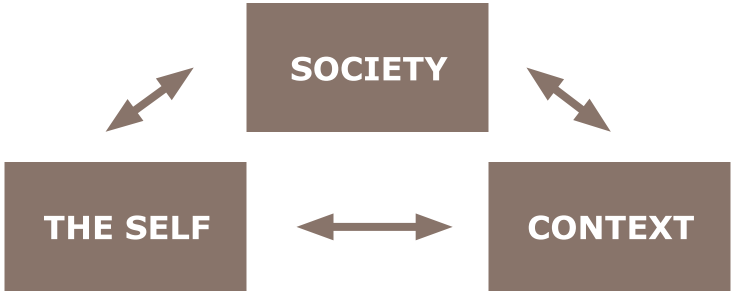

5) You don't have to be *stigmatised* to have self-stigma

Negative beliefs in the community about a condition like TB, HIV, or mental illness can contribute to a person's self-stigma.

Self-stigma includes internalized stigma. But an individual can already have self-stigma even if they've never actually been stigmatised.

What I think you think about me is not always true. A person can self-stigmatise up to three times as much as others actually stigmatise them.

4) What does self-stigma look like?

BELIEFS

Stereotype endoresement
"I believe TB is a sign of weakness"
Self-blame
"If I had taken better care of myself, I wouldn't have got TB"
Self-agency
"Having TB will affect my chances of keeping my job"

FEELINGS

Shame
"I get embarrassed because of having TB"
Guilt
"I can't forgive myself for exposing my family to TB"
Perception of what others think
"People will mistreat me because I have TB"

ACTIONS

Social withdrawal
"I keep my distance from others because I have TB disease"
Wny try?
"I won't go to school/work because I'm going to die anyway"
Negative coping
"I don't to to the clinic because people will know I have TB"

Annex 4 – Self-stigma learning tool 2: We are not alone

Self-stigma examples from all around the world.

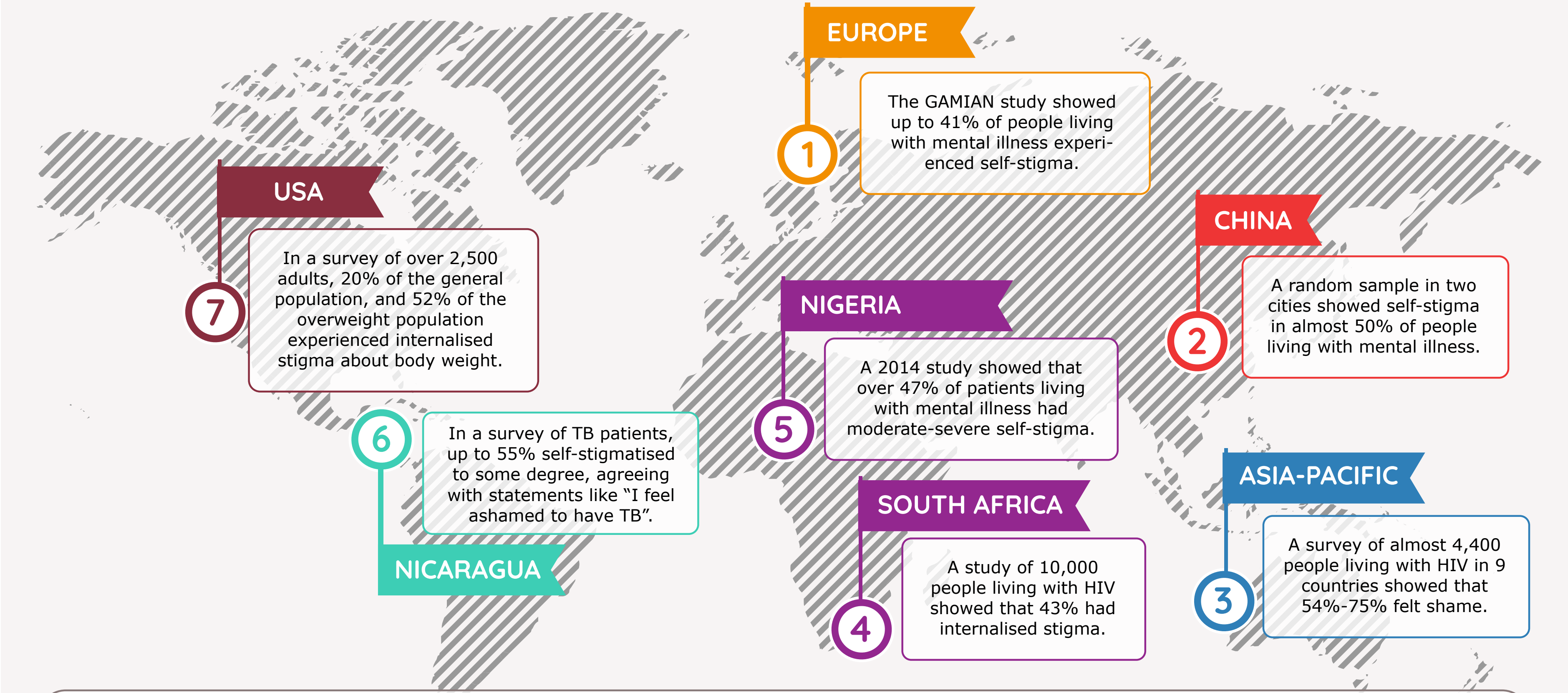

1) Self-stigma, empowerment and perceived discrimination among people with schizophrenia in 14 European countries: The GAMIAN-Europe study. Brohan et al. 2010
2) The prevalence and predictors of self-stigma of individuals with mental health illness in two Chinese cities. Young and Ng. 2016
3) People living with HIV Stigma Index: Asia Pacific Regional Analysis.Global Network of People Living with HIV and AIDS (GNP+) et al. 2011
4) The People Living with HIV Stigma Index: South Africa. South African National AIDS Council. 2014
5) A comparative study of self stigma between HIV/AIDS and schizophrenia patients. Oduguwa et al. 2014
6) Tackling tuberculosis patients' internalized social stigma through patient centred care: An intervention study in rural Nicaragua. Macq et al. 2008
7) Internalizing Weight Stigma: Prevalence and Sociodemographic Considerations in US Adults. Puhl et al. 2017

Annex 5 – Example participant profiles for Exercise 1.3

(These profiles are for illustrative purposes only. Audience- and country- specific profiles can be created by the facilitator team.)

1. The nervous participant

This individual is newly-diagnosed with TB and has never been involved in this type of activity before. They are extremely shy and may find it difficult to speak openly about their life experiences. They lack the confidence to express their opinion to others, and they feel like they have no control over their life.

2. The activist

This individual is in their late thirties and has been the founding member of several patient advocacy groups, as well as having spoken to policymakers to help create better conditions for themselves and others. They are not afraid to speak about their past experiences, and they feel like they are in full control of their life.

3. The self-stigmatizer

This individual is experiencing a high degree of self-stigma. They feel that because of their condition, their life is effectively over. They are not planning for their future, and because of the way that they have been treated in the past, they feel that someone with their condition is not allowed to join in discussions or to express their opinions to others.

Annex 6 - Journaling template for Exercise 2.1

My TB journal template

This is my journal on: Write today's date here

This is me, as I am today

Today I am feeling...	Describe how you feel, and what that means for you
What is on my mind today?	Write everything that is going on in your head

These are my negative thoughts

What negative thoughts have I had about TB today?	Write whatever negative thoughts arise
How does that make me feel?	Describe any emotions and feelings you have
Have I felt shame/self-stigma about TB today? If so, in what ways and how did I feel?	Write any experiences of self-stigma and shame you can identify
What would my closest family member or friend say to me today?	

These things were good today

Three things I am grateful for today:	Write ANYTHING you are grateful for, big or small
What am I proud of doing today?	Wite ANYTHING that you are proud of doing today

Annex 7 - Labels for Exercise 2.8 – Living Self-Stigma

Self-stigma

- shame
- guilt
- hopelessness
- worthlessness
- secretive
- hiding
- fearful to share information
- distrustful
- self-doubt
- low self-confidence

- high self-worth
- empowered
- confident
- strong self-agency
- high self-esteem
- open about TB diagnosis
- comfortable in your skin

Self-worth

Annex 8 - Handout for Exercise 2.10 – My right to tell

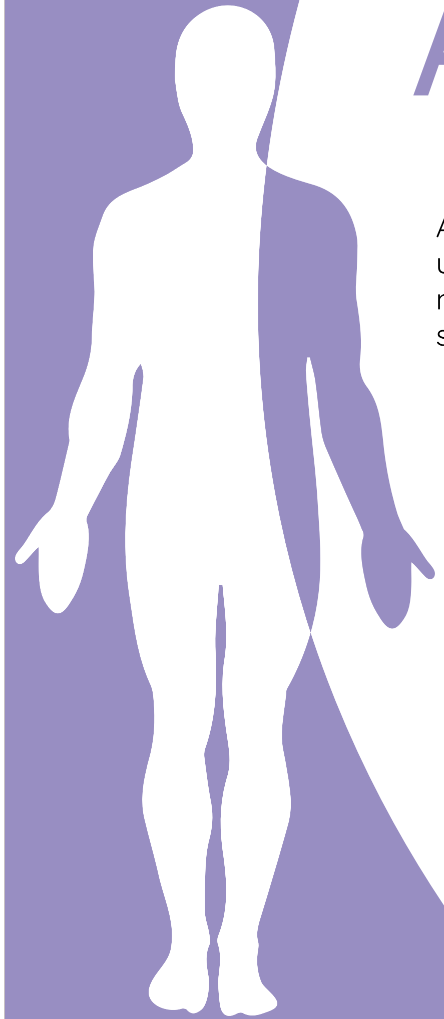

Adam is 37 years old with three children. His wife passed away six months ago, and he has started to have a prolonged cough and lose weight. One of his workmates has noticed he is not well and advises him to go to the clinic. Adam decides to go but he is worried he may have TB and maybe even be HIV positive. What will his family and friends say?

Adam goes to the clinic where a nurse does a TB test. The nurse tells him he has TB. This news upsets him – all he can think about is what people will say about him. The nurse tells him he must start treatment and gives him a number of instructions, but he hardly hears what she is saying. He takes the medicine she gives him and leaves the clinic in a confused state

After a few days, Adam returns to work. His colleague asks him, “What happened?” He says, “Nothing, I’m okay” and changes the topic of conversation. He feels like everyone is watching him and tries not to cough. When he is alone, he asks himself, “Who knows? How can I take treatment without people finding out? Will I lose my job? Are my children safe? Will they still be allowed go to School?”

Adam has an appointment to go back to the clinic. He is afraid to askpermission from his boss for more time off. He doesn’t know what to do.

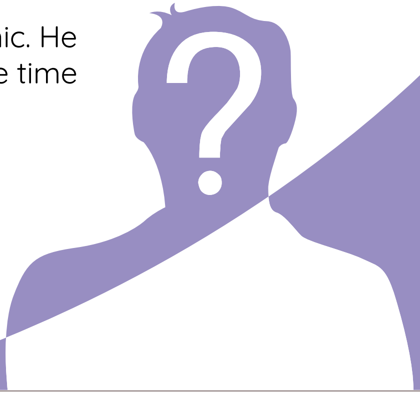

Adapted from: *STAMPP, CREATE, and International HIV/AIDS Alliance, (2009) Understanding and challenging TB stigma - Toolkit for action. 2009: ZAMBART Project, International HIV/AIDS Alliance.*

Annex 9 - Handout for Exercise 2.10 - Where am I on the disclosure continuum?

Social Avoidance: Stay away from others so they do not have a chance to stigmatise me

Pros	Cons

Secrecy: Go out into the world doing your usual daily activities – but tell no one about my illness

Pros	Cons

Selective disclosure: Tell people about my illness who seem like they will understand

Pros	Cons

Indiscriminate disclosure: Hide it from no-one

Pros	Cons

Broadcast: Be proud and let everyone know

Pros	Cons

Classifications taken from: On the Self-Stigma of Mental Illness: Stages, Disclosure, and Strategies for Change, Can J Psychiatry. 2012 Aug; 57(8): 464–469 doi: 10.1177/070674371205700804.

Annex 10 - Handout for Exercise 2.11 - Staying in your own business

There are only three kinds of business in the universe: mine, yours, and God's / The Universe's. [God's being the Universe or any God you may have in your life].

Whose business is it if I am feeling happy or sad? **My business.**

Whose business is it if you are feeling happy or sad? **Your business.**

Whose business is the weather? **God's / The Universe's business.** (Anything that's out of my control, your control, and everyone else's control.)

Much of our stress comes from mentally living out of our own business. When I think, "You need to get a job, I want you to be happy, you should be kinder, you should be on time, you need to take better care of yourself," I am in your business. When I'm worried about earthquakes, floods, war, or when I will die, I am in God's / The Universe's business. If I am mentally in your business or in God's / The Universe's business, the effect is separation and loneliness. If you are living your life and I am mentally living your life, who is here living mine? We're both over there. Being mentally in your business keeps me from being present in my own. I am separate from myself, wondering why my life doesn't work. Of course I feel lonely and separated! No one else causes my loneliness. I do that.

Notice when you feel loneliness or separation. Are you mentally out of your business? If you are not sure, stop and ask yourself, "Mentally, whose business am I in?" Notice when you give uninvited advice either out loud or silently. Whose business are you in when you are giving unsolicited advice?

Adapted from: © 2016 Byron Katie International, Inc. All rights reserved. thework.com (slight adaptation made)

Whose business is it? Check off your responses below:	my business	your business	God's business
My height	☐	☐	☐
That I have TB	☐	☐	☐
Attending a concert	☐	☐	☐
Rush-hour traffic	☐	☐	☐
My mother's depression	☐	☐	☐
Your judgements of me	☐	☐	☐
My judgements of you	☐	☐	☐
The weather	☐	☐	☐
My boss's anger	☐	☐	☐
My body	☐	☐	☐

Other's judgements of us can only have power if we believe them. We have no control over judgements made against us, but we can choose whether we believe them or not or whether we apply meaning to those judgements.

Annex 11 - Case studies for Exercise 3.1 – the many faces of TB

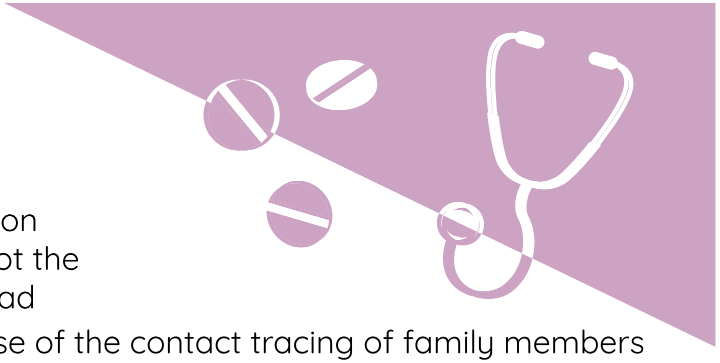

Story of Struggle with MDR-TB: Xolelwa's story

I was diagnosed with MDR-TB in 2012. I got it from my twin sister who has been on MDR-TB treatment for the last 4 years and has now developed XDR-TB. She got the TB germs from her friend who had MDR-TB. The strange thing is that I never had any external symptoms of TB and neither did I ever feel sick. It was just because of the contact tracing of family members of my twin sister that my MDR-TB status could be confirmed."

"MDR-TB treatment is really a difficult one. We have to take so many big pills and injections for such a long time. On top of this, most of us have to suffer from severe side effects. It makes one feel very miserable and angry that why I got TB at all. TB develops negative emotions in us which increase our day to day problems. There is still some stigma around the disease in the community and neighbourhood. If you have TB people stay away from you and tell you on your face not to come near them."

"It is here that counsellors and voluntary health workers can help, as they did in my case. They visited me at home, gave me a lot of moral support, and encouraged me to continue with the treatment. I got a lot of support from my family too, especially my twin sister. My friends were also quite okay with me. My boyfriend, whom I met three years ago, also stood by me. He is a social worker and also a cured TB survivor. Perhaps it was due to this that he did not let my TB affect our relationship."

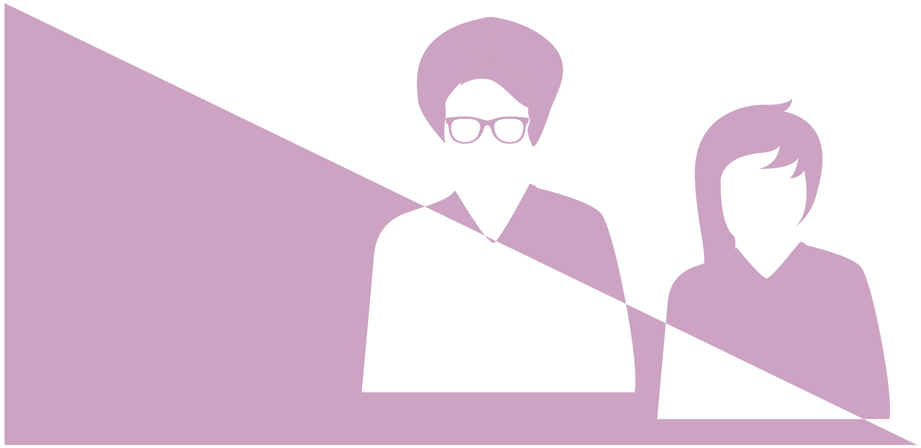

"My message to all those with TB or DR-TB is to be brave and to accept it. Acceptance always makes things easier. It becomes difficult to fight the disease if you are depressed and harbour negative thoughts."

Adapted from: CNS interview with Xolelwa Joni in November 2013: www.citizen-news.org/2013/11/story-of-struggle-of-mdr-tb-survivor.html

Rukmini’s story: persistent low-grade fever and cough…

Rukmini, who was battling MDR-TB in Ahmedabad, Gujarat, India, and spoke with CNS in March 2013: “My family is very supportive of me despite the infectious nature of the disease” said Rukmini. But unfortunately, Rukmini’s daughter contracted MDR-TB through her mother and has been on MDR-TB treatment in the same hospital since the last 6 months. The attending doctor, Dr Purvi told CNS that, “Direct MDR-TB transmission through contact is common. So, if a family member of such a MDR-TB patient has TB we test for MDR-TB in the beginning itself and if diagnosed put him/her directly on Category 4 treatment.”

Although her daughter contracted MDR-TB, better late than never on infection control. Healthcare workers “have really counselled me well and I follow all their instructions regarding infection control methods at home - I spit in a spittoon given by the hospital, I bury my spit in mud, I keep my house very clean. I hope I will soon be able to go back to my work of selling fruits and not remain a financial burden on my loving husband.

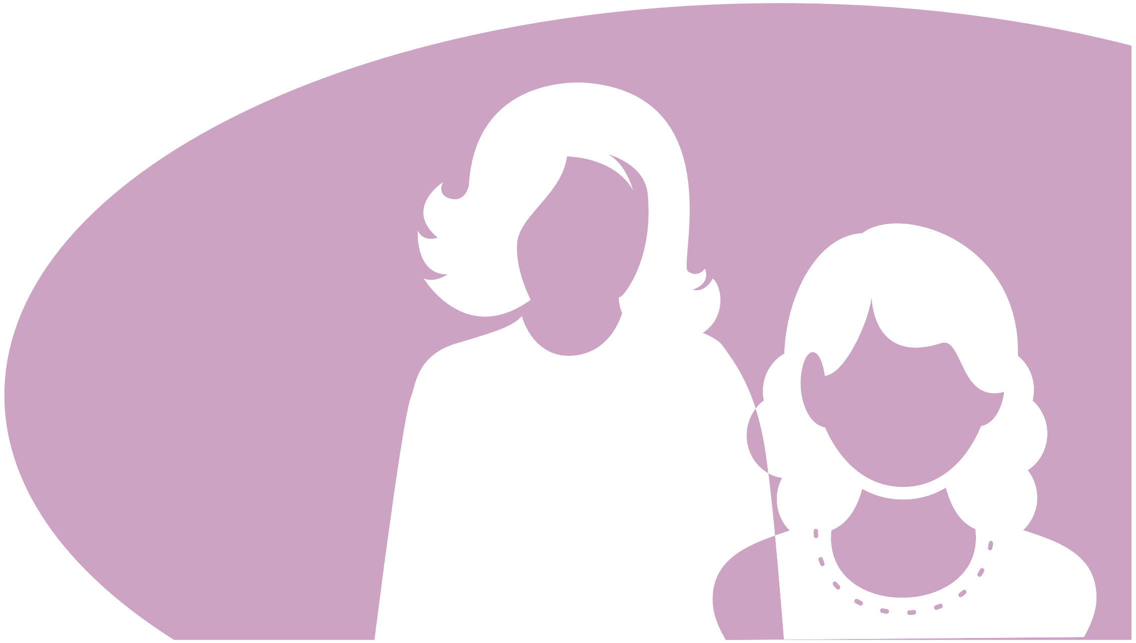

Adapted from: MDR-TB patient Rukmini’s interview with CNS in March 2013: http://www.citizen-news.org/2013/08/persistent-low-grade-fever-and-cough.html

Annex 12 – TB quiz for Exercise 4.2

This questionnaire will assess the knowledge level of participants, and to identify gaps in knowledge. Once you have identified these gaps, provide the necessary information to fill them in.

[Updated in 2017 from original source: International HIV/AIDS Alliance (2009). Understanding and challenging TB stigma: Toolkit for action.]

Answer the following questions by ticking either the true or false box	TRUE	FALSE
**General**		
1. TB is an infectious, airborne disease that only affects the lungs.	○	○
2. TB is becoming harder to treat.	○	○
3. TB can attack any part of the body, e.g. lungs, glands, brain, spine, hip, intestines, genitals, brain, eyes, etc	○	○
4. When the lungs are damaged by TB, a person coughs up sputum from the lungs and this produces TB droplets in the air.	○	○
5. Everyone who gets TB infection will become sick with TB disease.	○	○
6. Signs and symptoms of TB (especially TB of lungs) may include coughing, fever, sweating at night, loss of appetite, weight loss and feeling weak.	○	○
**Transmission**		
7. You get TB by breathing in germs in the air from a person with TB who is coughing.	○	○
8. All people who are coughing are infectious – they can spread TB to others.	○	○
9. You can get TB through shaking hands, touching or kissing someone who has TB.	○	○
10. You will 100% get TB through staying in the same house with someone who has TB.	○	○
11. You can still transmit TB to other people despite being cured	○	○
12. Health workers will 100% get TB because of frequent exposure to patients with infectious TB disease.	○	○

Answer the following questions by ticking either the true or false box

	TRUE	FALSE
**Myths and misconceptions**		
13. Having sex with a virgin cures TB.	○	○
14. Women have more TB germs. They are the ones who give men TB.	○	○
15. There are two types of TB – old TB and new (HIV-linked) TB.	○	○
16. People whose families have had TB (e.g. grandfather) are more at risk of getting TB.	○	○
**Prevention**		
17. Wearing masks is the best form of protection against TB.	○	○
18. Putting TB patients in isolated rooms is a good method for preventing TB.	○	○
19. Stopping the sharing of utensils is a good method for preventing TB.	○	○
20. It is important to get tested if you have a prolonged cough or cough up blood.	○	○
21. If you are coughing or sneezing you should cover your nose and mouth.	○	○
22. People living with HIV who don't have active TB disease but have latent TB infection, should take preventive therapy.	○	○
**TB testing and treatment**		
23. All forms of TB are diagnosed by examining the patient's sputum.	○	○
24. A person cannot take TB treatment at the same time as taking ARVs.	○	○
25. You can be cured of all forms of TB if you take treatment consistently for six or eight months.	○	○
26. The side effects of TB treatment are ...	○	○
27. During TB treatment eat well, avoid alcohol and tobacco, and avoid stress.	○	○

Answers for TB Quiz

General	Answer
1. TB is an infectious, airborne disease that only affects the lungs.	FALSE. TB is a disease caused by a germ called mycobacterium tuberculosis. TB can affect any part of the body. When TB disease affects the lungs then it is called Pulmonary TB. TB of other parts of the body is called Extra-Pulmonary TB. Pulmonary TB disease is infectious, but extra-pulmonary TB is not. People living with HIV often develop Extra-Pulmonary TB.
2. TB is becoming harder to treat.	TRUE. Over time, the TB germ slowly works out how to resist being killed by the major TB drugs, especially when the drugs are not taken properly. Some strains or specific varieties of TB have become resistant to at least one of the major anti-TB drugs, meaning that fewer weapons are available to fight the infection. These strains are called drug-resistant TB.
3. TB can attack any part of the body, e.g. lungs, glands, brain, spine, hip, intestines, genitals, brain, eyes, etc.	TRUE. The most common part of the body to be affected by TB disease is the lungs, but TB also attacks other parts of the body.
4. When the lungs are damaged by TB, a person coughs up sputum from the lungs and this produces TB droplets in the air.	TRUE. The person coughs up sputum or mucus from the lungs and it produces TB droplets in the air.
5. Everyone who gets TB infection will become sick with TB disease.	FALSE. Not everybody who breathes in TB germs will get TB disease. Latent TB infection is when TB bacteria is in the lungs but is not causing any disease. Many people in TB high burden nations may have latent TB. Latent TB is not infectious.<br>But in 1 in 10 people latent TB infection may develop into active TB disease. Those with immune-compromised situations like HIV may be at higher risk of developing active TB disease, which may be infectious (if TB disease is of lungs).
6. Signs and symptoms of TB (especially TB of lungs) may include coughing, fever, sweating at night, loss of appetite, weight loss and feeling weak.	TRUE. A person with TB may experience coughing, fever, sweating at night, loss of appetite, weight loss, and feeling weak. These symptoms usually are of TB of the lungs. TB disease of other parts of the body such as eyes or genitals may have different symptoms and different samples are needed for confirming TB diagnosis (not sputum).

Answers

Transmission	Answer
7. You get TB by breathing in germs in the air from a person with TB who is coughing.	TRUE. People with infectious TB, release TB droplets into the air through coughing. A person who is in the same room as the source who breathes in the droplets can become infected.
8. All people who are coughing are infectious – they can spread TB to others.	FALSE. Only people with TB who are sputum positive or 'infectious' can transmit the germs to other people through a cough. There are other diseases which may cause coughing too. For prolonged cough it is a good practice to seek medical advice – and get treated for the disease which is causing cough.
9. You can get TB through shaking hands, touching or kissing someone who has TB.	FALSE. TB is not transmitted through touch or physical contact. In the case of kissing, TB bacilli are produced deep in the lungs, not in saliva, so there is no risk of getting TB through kissing.
10. You will 100% get TB through staying in the same house with someone who has TB.	FALSE. Best practices in transmission control can prevent transmission of TB infection in homes, communities, healthcare facilities. If transmission control practices are not followed in homes, communities, and/or healthcare facilities, then risk of transmission of TB shoots up.
11. You can still transmit TB to other people despite being cured	FALSE. There is a possibility of transmission of TB when one is on treatment, but the risk is reduced when patients become sputum negative in case of TB of the lungs, or when doctors confirm from laboratories that they have become culture negative in case of TB of other parts of body, as well as drug-resistant TB.
12. Health workers will 100% get TB because of frequent exposure to patients with infectious TB disease.	FALSE. With standard transmission control practices in place in healthcare facilities, communities, and homes, the risk of TB transmission is negligible. But if standard transmission control practices are not followed in healthcare facilities, communities and homes, then risk of TB transmission goes up.

Answers

Myths and misconceptions	Answer
13. Having sex with a virgin cures TB.	FALSE. Virgins do not have any power to heal TB-infected individuals, just as they cannot heal HIV-infected individuals. Having sex with a minor puts them at a lot of health risks.
14. Women have more TB germs. They are the ones who give men TB.	FALSE. Globally TB incidence is higher in males than females, except in a few nations, like Afghanistan. But gender-based inequalities may jeopardize access to TB care for women, and fuel multiple stigma and discrimination.
15. There are two types of TB – old TB and new (HIV-linked) TB.	FALSE. There is no old or new TB. It is true that people living with HIV are at increased risk of TB because of the weakened immune system.
16. People whose families have had TB (e.g. grandfather) are more at risk of getting TB.	FALSE. TB is transmitted through the air, not by our genes.

Prevention	Answer
17. Wearing masks is the best form of protection against TB.	FALSE. Standard masks are of little value. High quality masks such as N-95 masks might reduce risk, but are very expensive. Also, good ventilation, exposure to sunlight, and all other standard transmission control practices in all healthcare facilities, communities, and homes are important to cut the chain of TB transmission.
18. Putting TB patients in isolated rooms is a good method for preventing TB.	FALSE. Isolating TB patients is not a good method for preventing TB; it also promotes stigma. However, all standard transmission control practices must be followed in all healthcare facilities and community settings as well as within homes to cut the chain of TB transmission.
19. Stopping the sharing of utensils is a good method for preventing TB.	FALSE. TB cannot be transmitted through sharing eating utensils. Everyone can use the same utensils. Stopping the sharing of utensils promotes stigma.
20. It is important to get tested if you have a prolonged cough or cough up blood.	TRUE. Taking a test helps you know if you have TB so you can start treatment early. Also, there are other diseases that may be causing the cough, so it is good practice to seek medical advice for prolonged cough.

Answers

21. If you are coughing or sneezing you should cover your nose and mouth.	TRUE. The recommended practice is to lift the arm up and cover the nose and mouth with the inner surface of the arm when coughing or sneezing. This is to keep germs away from the hands. The hands cannot spread TB, but other respiratory germs such as influenza can.
22. People living with HIV who don't have active TB disease but have latent TB infection, should take preventive therapy.	TRUE. People living with HIV who don't have active TB disease but have latent TB infection can take TB preventive therapy.

TB testing and treatment	Answer
23. All forms of TB are diagnosed by examining the patient's sputum.	FALSE. Examining sputum is the test for TB of the lungs (pulmonary TB). TB of other body parts (such as brain, genitals, eyes, etc.) may need different samples from affected sites to be tested for TB.
24. A person cannot take TB treatment at the same time as taking ARVs.	FALSE. One can take TB treatment and ARVs. It is important that she/ he seeks advice from qualified health workers at the health center to ensure there is no drug-drug interaction and patient is stabilized on both anti-TB treatment and ARVs.
25. You can be cured of all forms of TB if you take treatment consistently for six or eight months.	FALSE. Drug-sensitive TB treatment lasts for six months or more, but treatment duration for drug-resistant TB may last up to two years or more. It is important to adhere the TB therapy and be treated with drugs that a person is sensitive to (and not resistant to) to get cured.
26. The side effects of TB treatment are ...	Some of the side effects of TB treatment include nausea, abdominal pains, pain in joints, and burning sensation in the feet. Severe side effects include ringing in the ears, deafness, severe skin rash, dizziness, yellowness of the eyes, poor sight, and loss of color perception. If one presents any of the above, they should seek medical attention from qualified medical personnel at the clinic.
27. During TB treatment eat well, avoid alcohol and tobacco, and avoid stress.	TRUE. The body needs nutritious foods for energy and strength to fight infections and sicknesses. Alcohol and cigarettes can damage the body's ability to fight off diseases, so stopping or reducing their consumption can help to keep the body strong.

Annex 13 – Case studies for Exercise 5.1 – Rights and the patient with TB

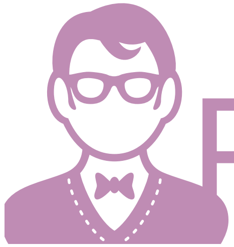

Robert is a married man with three children. He and his family were chased out of his house when his landlord discovered that he had TB. The landlord said he didn't want Robert to infect other people and that it would be bad for his business.

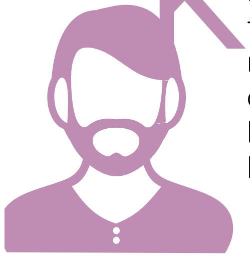

Kenneth has had TB for the last three months and is responding to treatment well. He stays with his family and while he is there, the family starts planning the wedding for his youngest sister. Kenneth asks to help with the wedding arrangements, but his father tells him, "People like you don't need to be involved in these things".

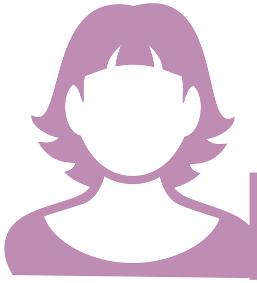

Natalie has been on TB treatment for two weeks and has not been responding well. She is very sick. Her family calls a meeting and decides that she should stop taking the drugs and go to her grandmother's house in the village where she can rest and recover.

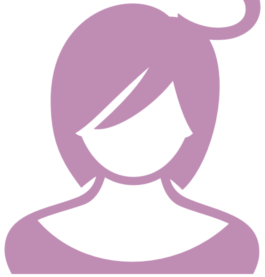

Selina is a young woman who is living with her grandmother. She was diagnosed with TB a few months ago and has been responding well to treatment. Recently she met a young man whom she really likes and hopes to marry one day. However, her grandmother has told her that she cannot be in a relationship – she must wait until her TB treatment is finished and she is sure that she is well.

Adapted from: International HIV/AIDS Alliance (2009). Understanding and challenging TB stigma: Toolkit for action.

Annex 14 - Card game for Exercise 5.2 – Perfectly imperfect

If possible, these should be printed in color, on high-quality card so that they can be easily re-used. Print out each sheet of cards double-sided with the appropriate back matching the type of card on the front (see below).

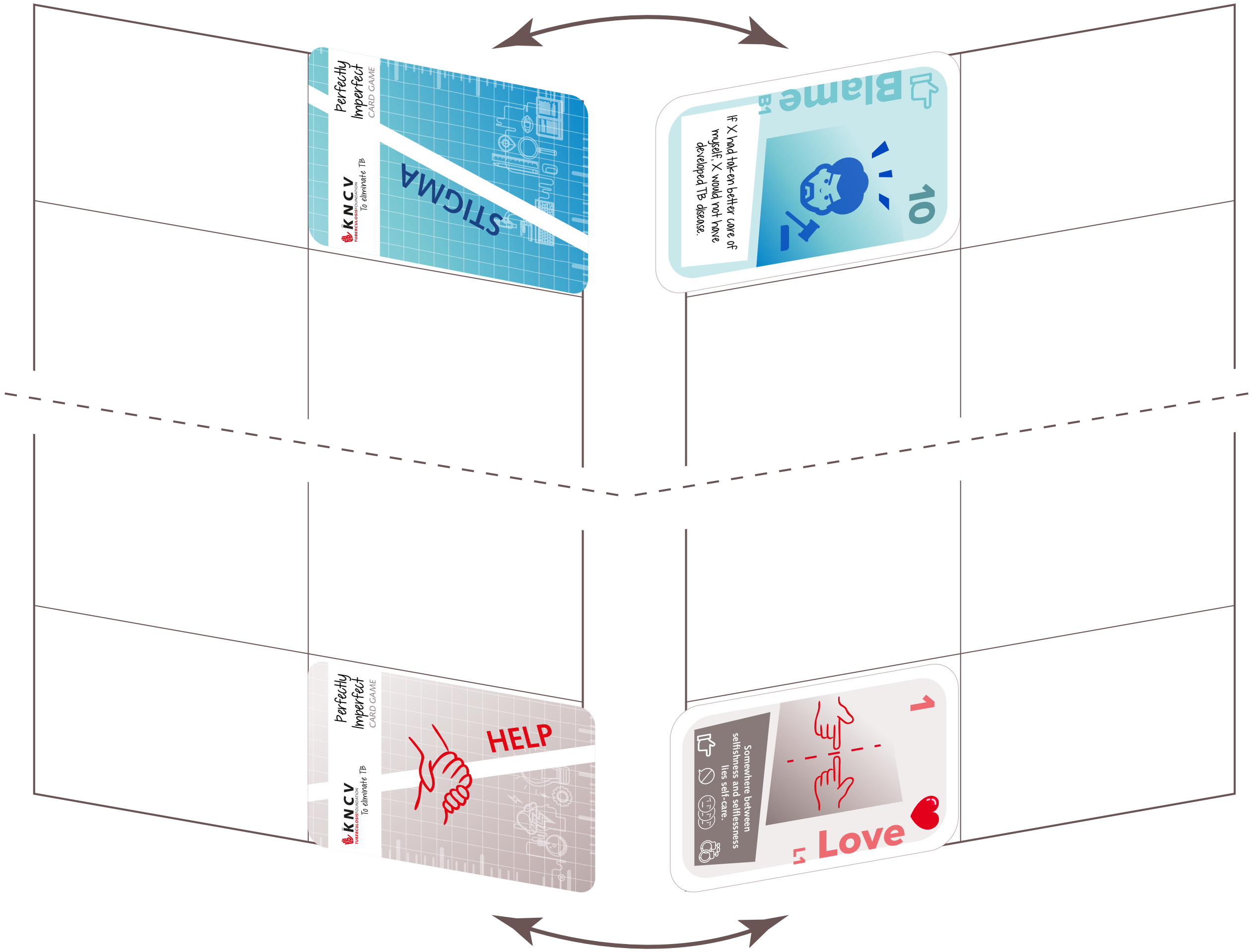

Perfectly Imperfect

SELF-STIGMA CARD GAME

KNCV TUBERCULOSIS FOUNDATION
To eliminate TB

STIGMA CARD (BACK)

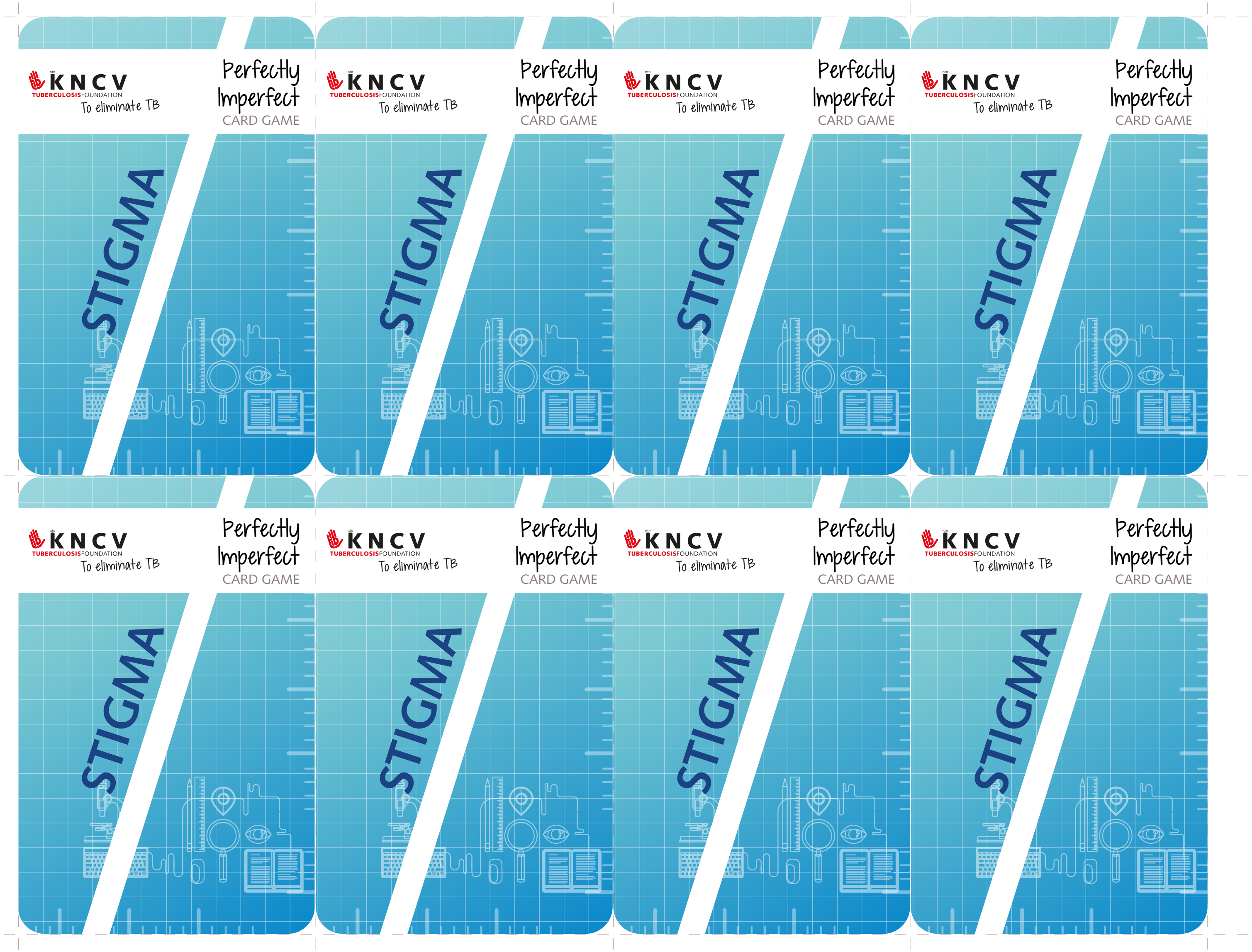

Perfectly Imperfect

SELF-STIGMA CARD GAME

KNCV TUBERCULOSIS FOUNDATION
To eliminate TB

STIGMA CARDS (FRONT)

10 Blame B1

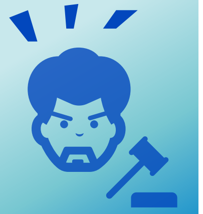

If X had taken better care of myself, X would not have developed TB disease.

9 Blame B2

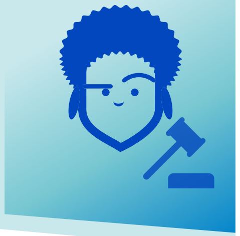

If X had taken my TB treatment as instructed, X wouldn't have developed drug-resistant TB.

8 Blame B3

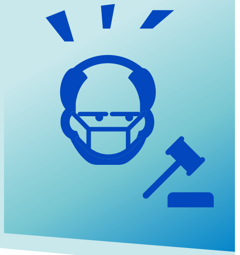

If X had taken my ART and IPT as instructed, X would not have developed TB disease.

7 Blame B4

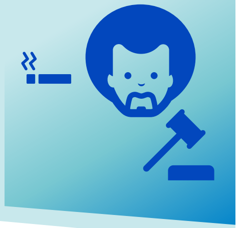

If X had not smoked, X would not have gotten TB.

6 Blame B5

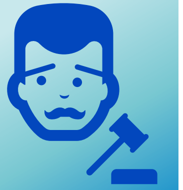

If X had not been drinking or drugging X would not have gotten TB.

5 Blame B6

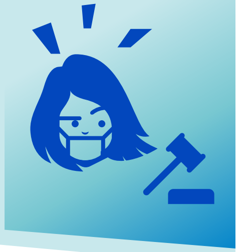

If X had not been detained, X would not have gotten TB.

6 Blame B7

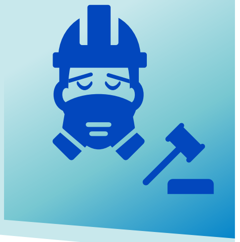

If X had worn my respirator at work, X would not have gotten TB.

7 Blame B8

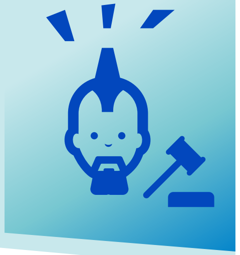

If X had not spent time in certain places, X would not have gotten TB.

Perfectly Imperfect

SELF-STIGMA CARD GAME

KNCV TUBERCULOSIS FOUNDATION
To eliminate TB

STIGMA CARDS (FRONT)

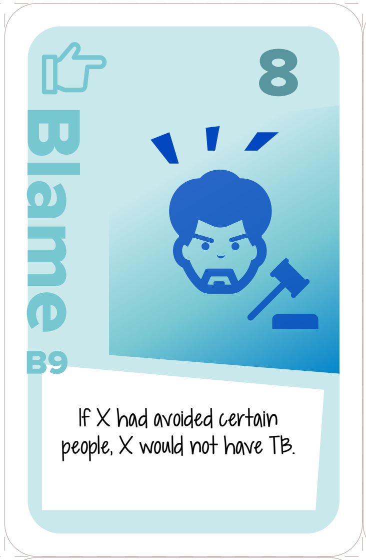

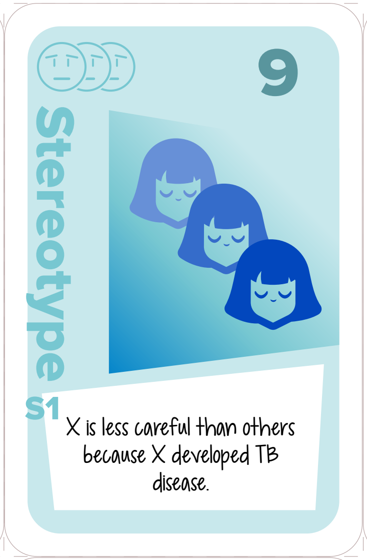

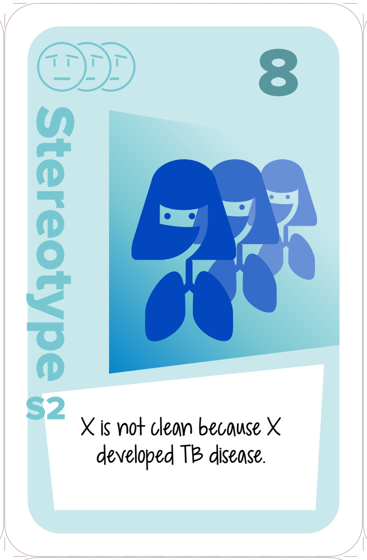

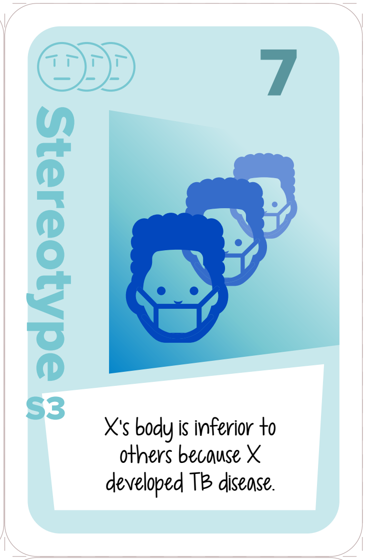

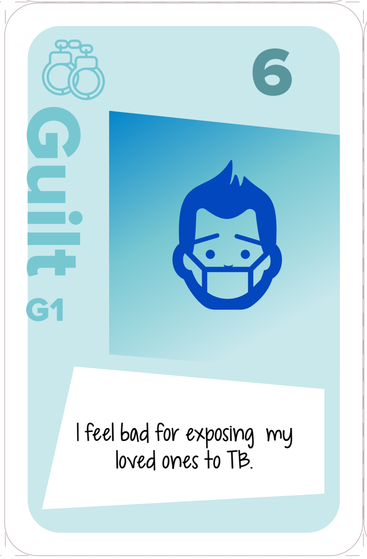

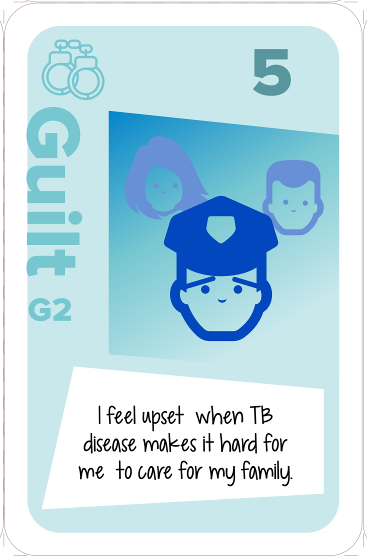

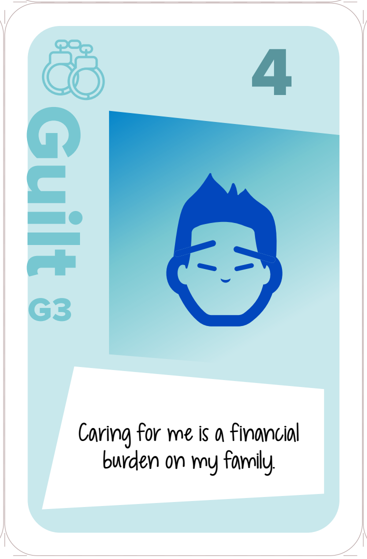

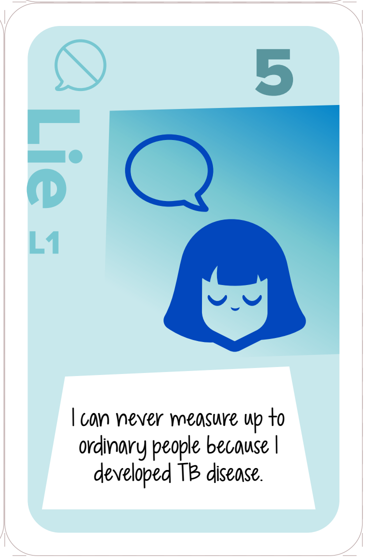

STIGMA CARDS (FRONT)

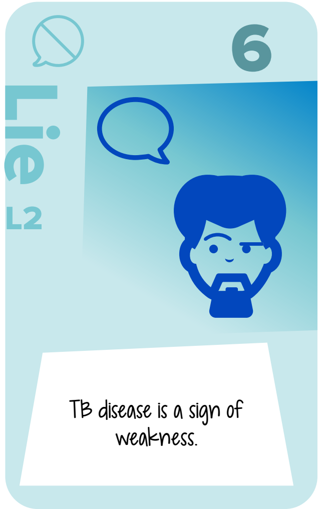

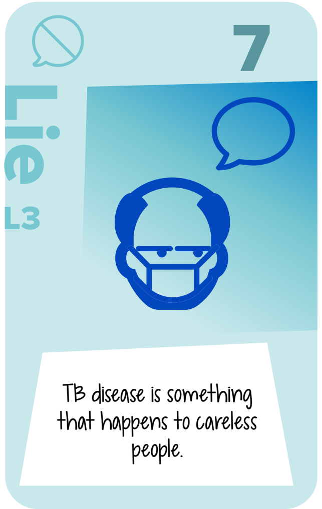

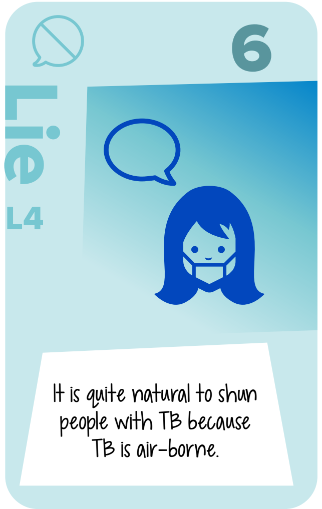

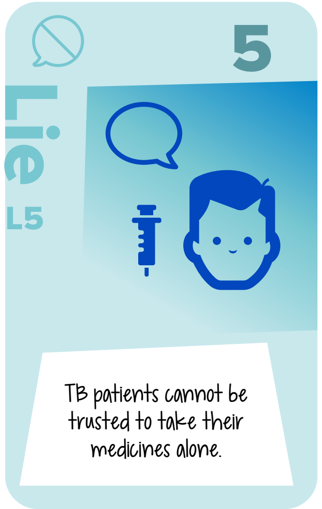

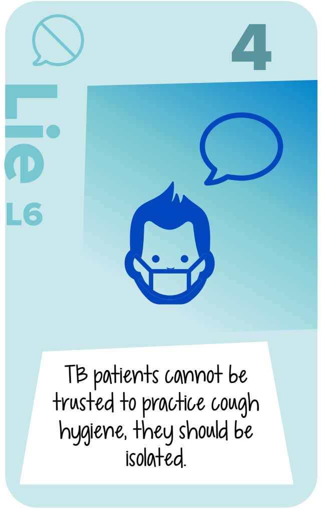

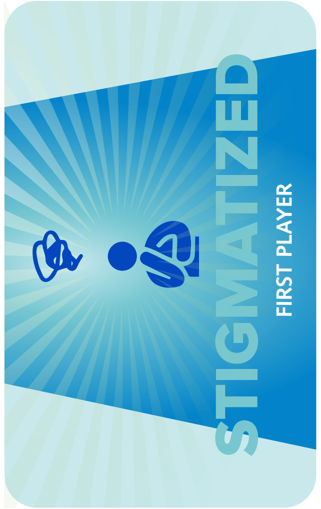

FIRST PLAYER CARD (FRONT)

HELP CARD (BACK)

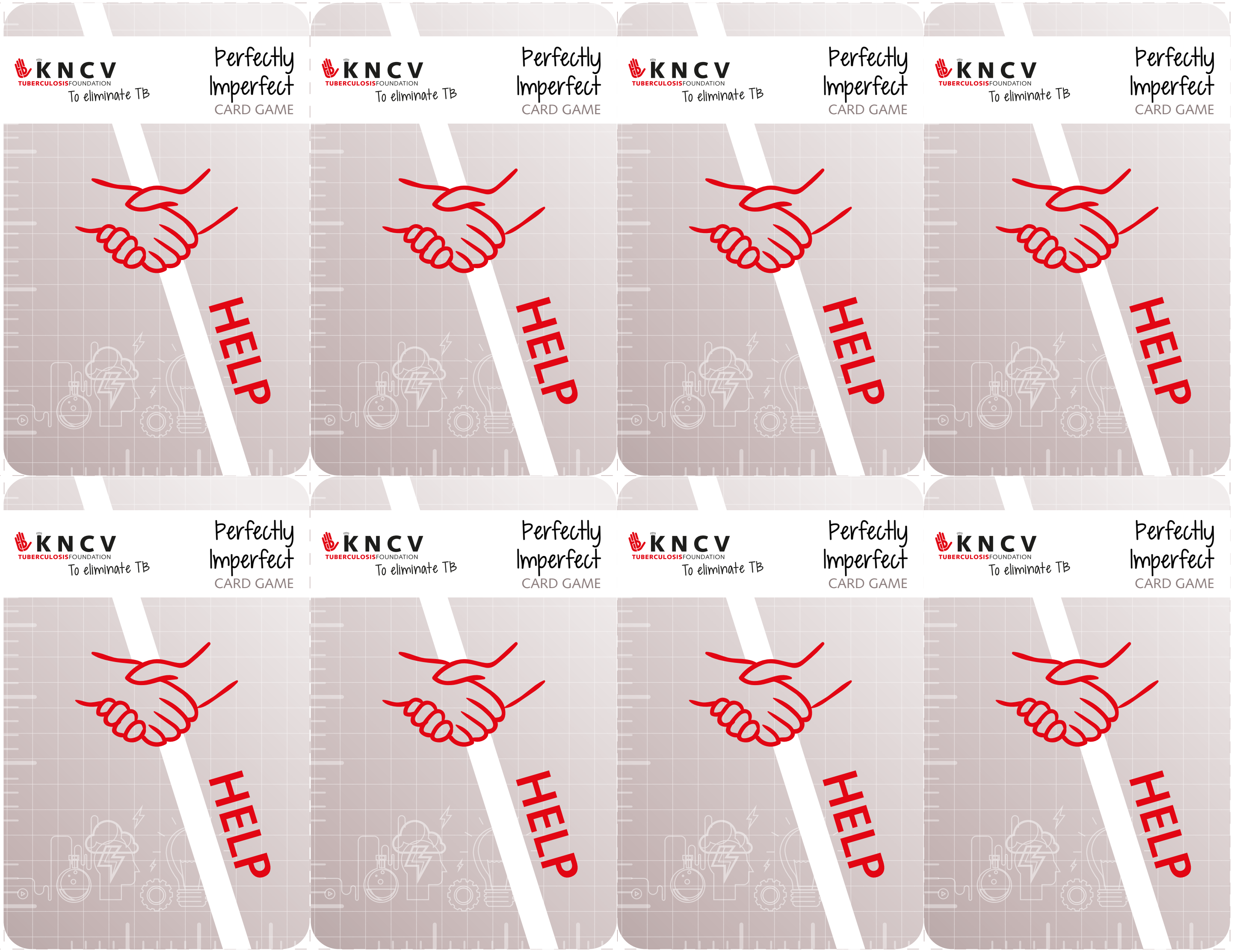

Perfectly Imperfect

SELF-STIGMA CARD GAME

KNCV TUBERCULOSIS FOUNDATION
To eliminate TB

HELP CARD (FRONT)

5 Truth T1
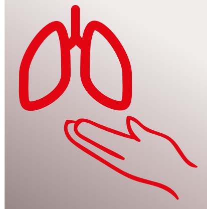
TB is caused by breathing it is nobody's fault.

2 Truth T2
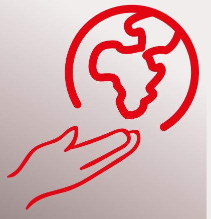
20% of the world's population has TB infection.

3 Truth T3
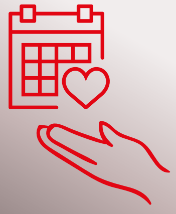
Once on effective TB treatment, a person stops being infectious in about 2 weeks.

5 Truth T4
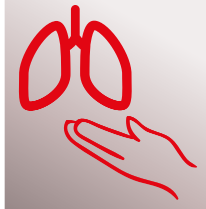
TB is caused by breathing it is nobody's fault.

1 Love L1
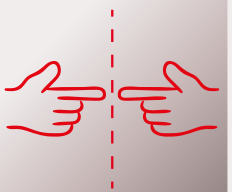
Somewhere between selfishness and selflessness lies self-care.

2 Love L2
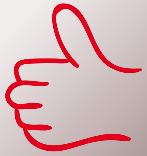
Forgiveness of myself and others releases me from the past.

3 Love L3
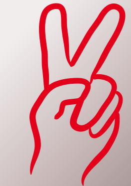
Forgiveness is the answer to many problems.

4 Love L4
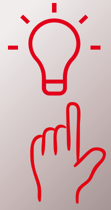
Though these times are difficult, they are teaching me many things.

Perfectly Imperfect

SELF-STIGMA CARD GAME

KNCV TUBERCULOSISFOUNDATION To eliminate TB

HELP CARD (FRONT)

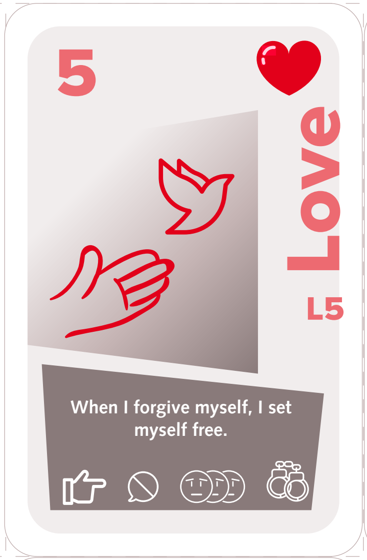

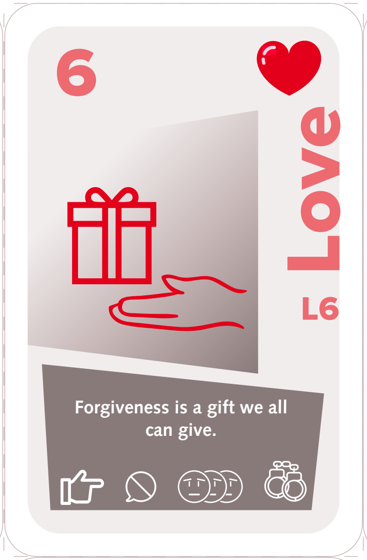

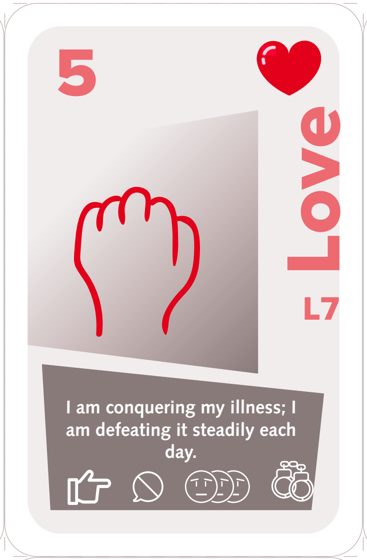

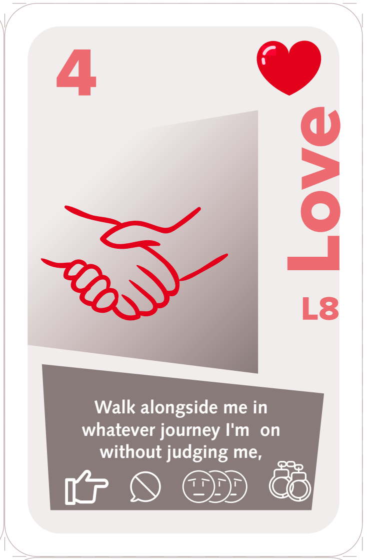

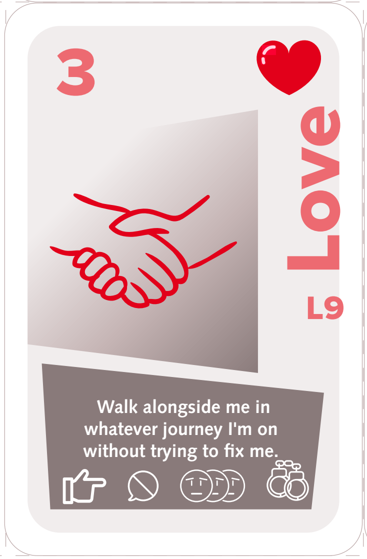

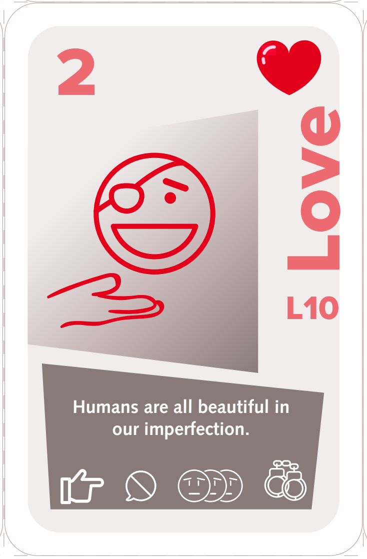

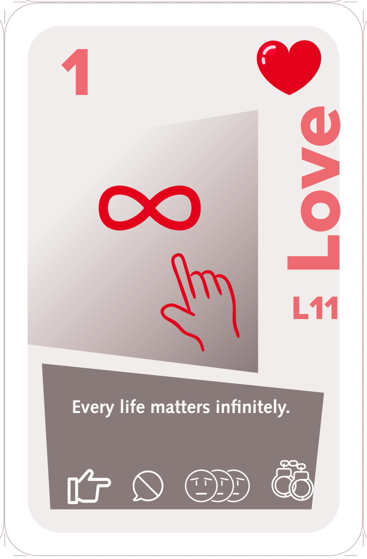

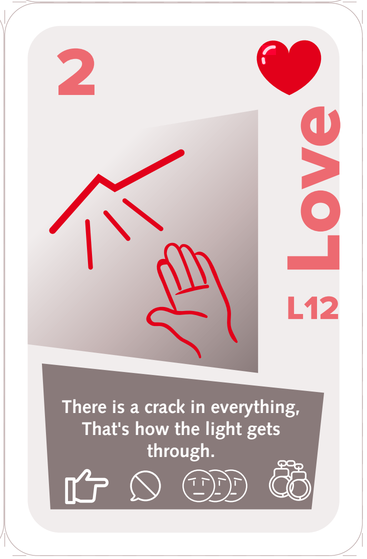

KNCV TUBERCULOSISFOUNDATION To eliminate TB

HELP CARD (FRONT)

3 Love L13
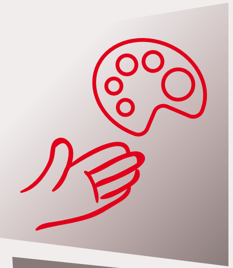
To be creative is to be come home to ourselves.

4 Love L14
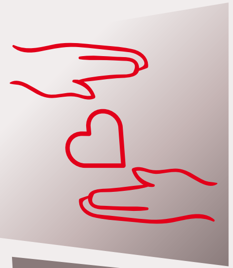
Love weaves beauty out of pain.

5 Love L15
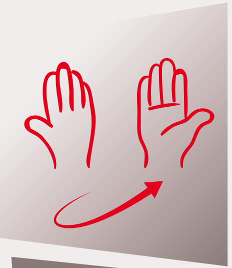
What you see on the outside is not all I am on the inside.

4 Love L16
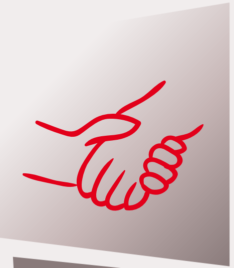
Solidarity is the tenderness of the people.

3 Love L17
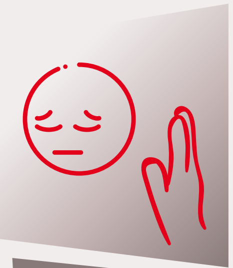
No one can make you feel inadequate without your consent.

2 Love L18
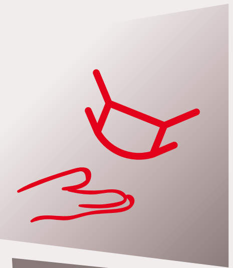
Allow people to make different decisions and to have different experiences than you would.

1 Love L19
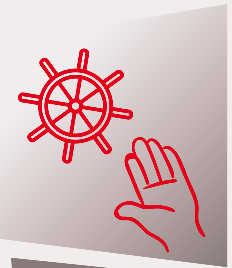
Release control and we honour differences.

2 Love L20
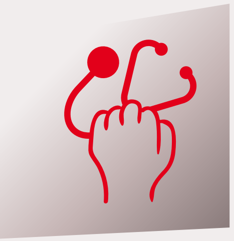
Treating yourself with care, consideration, kindness, compas-sion and love is part of recovering from a health problem.

Perfectly Imperfect

SELF-STIGMA CARD GAME

KNCV TUBERCULOSIS FOUNDATION
To eliminate TB

HELP CARD (FRONT)

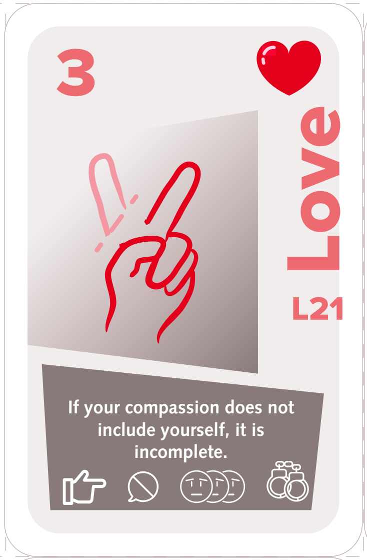

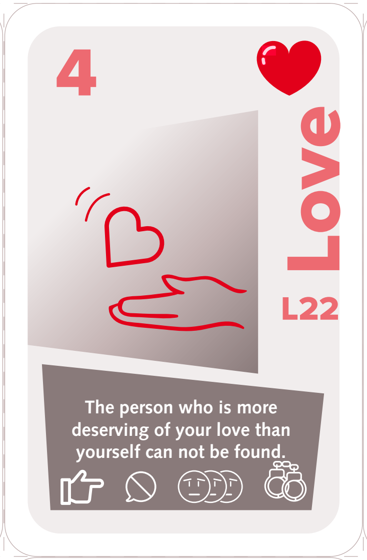

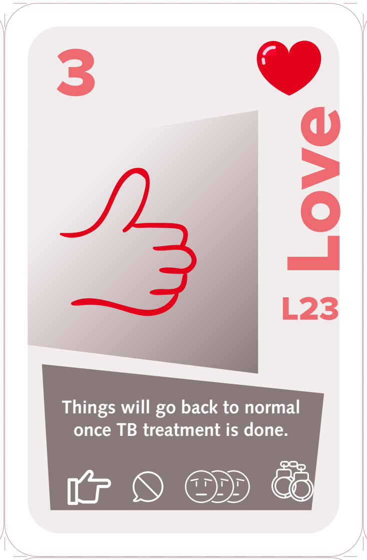

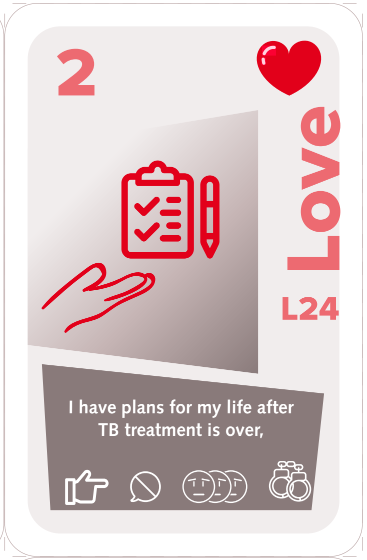

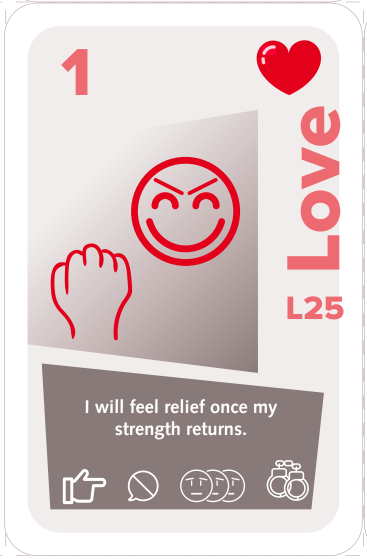

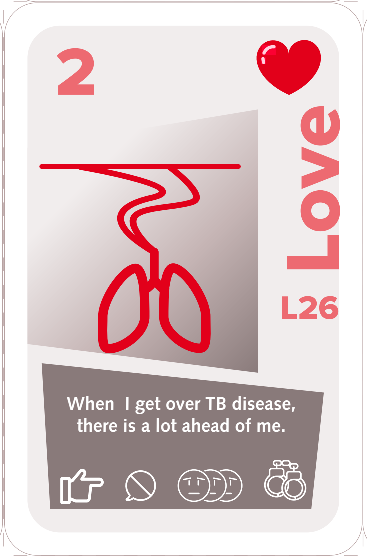

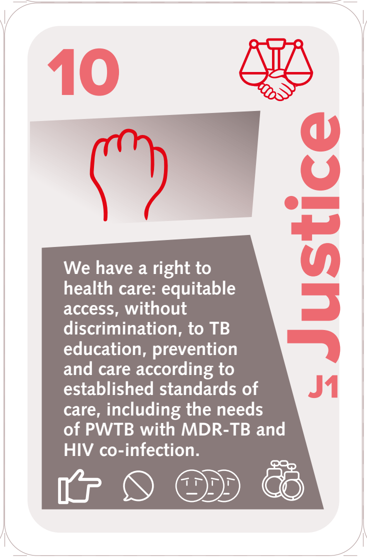

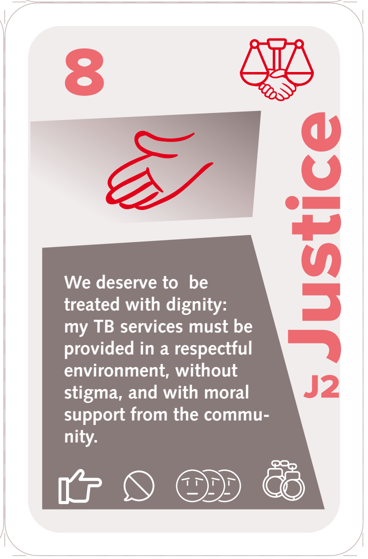

Perfectly Imperfect

SELF-STIGMA CARD GAME

KNCV TUBERCULOSISFOUNDATION To eliminate TB

HELP CARD (FRONT)

2 Justice J3

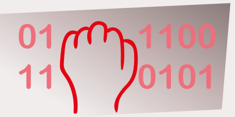

We have a right to Information: on all aspects of TB, including prognosis, costs, side effects, and other consequences, and to share experiences with peers.

4 Justice J4

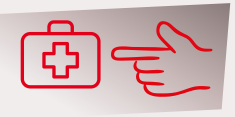

We have choices: to have a second opinion, access to medical records, accept or refuse medical interventions and to take part-or not-in research.

2 Justice J5

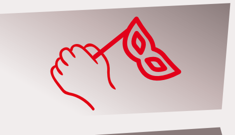

My right to privacy must be respected: uphold patient-provider confidentiality laws, in contact investigation.

4 Justice J6

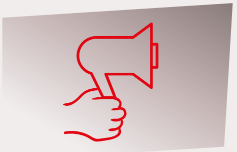

We deserve justice: we have the right to complain, to appeal and to be heard promptly and fairly.

3 Justice J7

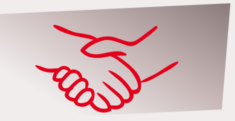

We have the right to organize: to participate as stakeholders in policies and programs and establish TB survivor platforms.

7 Justice J8

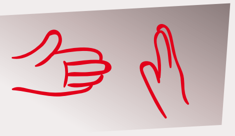

We have the right to protection from discrimination: my job security should not be threatened while I am recovering.

5 Justice J9

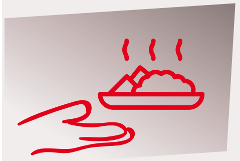

We deserve sustenance: nutrition security or food supplements if needed.

6 Justice J10

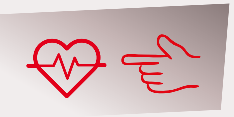

We have a right to life: States must adopt measures in law and policy to protect the lives of people with TB, including ensuring access to testing and life saving treatment.

HELP CARD (FRONT)

6 — Justice J11

We have a right to medicine: States have a core obligation to provide essential medicine on the WHO Model List of Essential Medicines-including 1st/2nd-line anti-TB drugs, bedaqiline and delamanid

7 — Justice J12

We have a right to be free from discrimination: People with TB must be protected by law against discrimination in both the public and private spheres, including employment, education, housing, health care settings, etc.

5 — Justice J13

We have the right to be free from torture and other cruel, inhuman or degrading treatment or punishment: Law and policy must ensure people in prisons and other detention centers are provided clean air, TB testing and treatment services

4 — Justice J14

We have the right to liberty and security of person: Law and policy must establish clear protections against involuntary detention or isolation of people with TB, except under exceptional circumstances, as a last resort, or when a person is known to be or highly likely to be contagious but refuses treatment or testing and all reasonable measures have been unsuccessfu-then the least restrictive possible measure must be used.

Hurray! You are the most knowledgeable player — VICTORY CARD

Yes! You are the most empathetic player — VICTORY CARD

Wow! You are the most righteous player — VICTORY CARD

VICTORY CARD (FRONT)

Annex 15 - Example baseline-endline assessment of participants

This example baseline-endline measurement will help implementers to assess levels of self-stigma among participants, levels of efficacy around self-stigma, and levels of self-compassion, pre- and post-interventions. It uses items from the validated Van Rie Patient Perspectives towards tuberculosis, and the Neff self-compassion scales, as well as original questions.

Part 1: Van Rie Patient Perspectives Towards Tuberculosis							
**Question**	**Strongly disagree**	**Disagree**	**Slightly disagree**	**Not sure**	**Slightly agree**	**Agree**	**Strongly agree**
Some people who have TB feel hurt by how others react to knowing they have TB.	1	2	3	4	5	6	7
Some people who have TB lose friends when they share with them they have TB.	1	2	3	4	5	6	7
Some people who have TB feel alone.	1	2	3	4	5	6	7
Some people who have TB keep their distance from others to avoid spreading TB germs.	1	2	3	4	5	6	7
Some people who have TB are afraid to tell those outside their family that they have TB.	1	2	3	4	5	6	7
Some people who have TB are afraid of going to TB clinics because other people may see them there.	1	2	3	4	5	6	7
Some people who have TB are afraid to tell others that they have TB because others may think that they also have AIDS.	1	2	3	4	5	6	7
Some people who have TB feel guilty because their family has the burden of caring for them.	1	2	3	4	5	6	7
Some people who have TB will choose carefully who they tell about having.	1	2	3	4	5	6	7
Some people who have TB feel guilty for getting TB because of their smoking, drinking, or other careless behaviours.	1	2	3	4	5	6	7
Some people who have TB are worried about having AIDS.	1	2	3	4	5	6	7
Some people who have TB are afraid to tell their family that they have TB.	1	2	3	4	5	6	7

Part 2: Self-stigma knowledge and efficacy							
**Question**	**Strongly disagree**	**Disagree**	**Slightly disagree**	**Not sure**	**Slightly agree**	**Agree**	**Strongly agree**
I know what self-stigma is.	1	2	3	4	5	6	7
I can give examples of the manifestations of self-stigma.<br>Please give examples of the manifestations of self-stigma that you know:	1	2	3	4	5	6	7
I know the negative effects of self-stigma.	1	2	3	4	5	6	7

Part 2: Self-stigma knowledge and efficacy							
**Question**	**Strongly disagree**	**Disagree**	**Slightly disagree**	**Not sure**	**Slightly agree**	**Agree**	**Strongly agree**
I can give examples of the effects of self-stigma.<br>Please give examples of the effects of self-stigma that you know:	1	2	3	4	5	6	7
I am equipped with techniques to overcome self-stigma.	1	2	3	4	5	6	7
I can give examples of techniques to overcome self-stigma.<br>Please give examples of the techniques that you know to overcome self-stigma:	1	2	3	4	5	6	7
I have tools to overcome self-stigma in myself.	1	2	3	4	5	6	7
I can assist other people who are experiencing self-stigma.	1	2	3	4	5	6	7

Part 3: Self-compassion					
**Question**	**Strongly dis-agree**	**Disagree**	**Not sure**	**Agree**	**Strongly agree**
When I fail at something important to me, I become consumed by feelings of inadequacy.	1	2	3	4	5
I try to be understanding and patient towards those aspects of my personality I don’t like.	1	2	3	4	5
When something painful happens I try to take a balanced view of the situation.	1	2	3	4	5
When I’m feeling down, I tend to feel like most other people are probably happier than I am.	1	2	3	4	5
I try to see my failings as part of the human condition.	1	2	3	4	5
When I’m going through a very hard time, I give myself the caring and tenderness I need.	1	2	3	4	5
When something upsets me, I try to keep my emotions in balance.	1	2	3	4	5
When I fail at something that’s important to me, I tend to feel alone in my failure.	1	2	3	4	5
When I’m feeling down I tend to obsess and fixate on everything that’s wrong.	1	2	3	4	5
When I feel inadequate in some way, I try to remind myself that feelings of inadequacy are shared by most people.	1	2	3	4	5
I’m disapproving and judgmental about my own flaws and inadequacies.	1	2	3	4	5
I’m intolerant and impatient towards those aspects of my personality I don’t like.	1	2	3	4	5

Annex 16 - Example facilitator pilot feedback form

This form can be completed by facilitators, and provides useful information for future adjustments to the package.

Self-stigma toolkit facilitator feedback form<br>Please complete all sectionss	
What exercises did you use?	
What kind of training did you use the exercises for and who was the target audience?	
Which exercises worked well? Why?	
Which exercises did not work well? Why?	
Please describe any changes or improvements you made to any exercises.	
What ADDITIONAL TOPICS need to be added to the toolkit?	
Have you developed any case studies or other materials on stigma? If yes, please describe and, if possible, send them to KNCV.	
Please provide your name and email address so we can follow up.	

Annex 17 - Example participant workshop feedback form

This form can be given to participants to complete following the workshop session.

PARTICIPANT WORKSHOP FEEDBACK FORM
Please choose one response to each question
Possible responses:
1 = Strongly disagree; 2 = Disagree; 3 = Slightly disagree; 4 = Not sure; 5 = Agree a little; 6 = Agree; 7 = Strongly Agree
Or write responses where indicated

About the organization of the workshop	Strongly disagree	Disagree	Slightly disagree	Not sure	Slightly agree	Agree	Strongly agree
We had enough time to complete the exercises during the workshop.	1	2	3	4	5	6	7
Our needs were met during the workshop.	1	2	3	4	5	6	7
**About the workshop venue**	**Strongly disagree**	**Disagree**	**Slightly disagree**	**Not sure**	**Slightly agree**	**Agree**	**Strongly agree**
The workshop venue was comfortable.	1	2	3	4	5	6	7
The air was safe.	1	2	3	4	5	6	7
The workshop venue had enough sound and visual privacy.	1	2	3	4	5	6	7
The workshop venue was easily accessible (e.g., for participants with disabilities).	1	2	3	4	5	6	7
Please give any further feedback about the workshop venue:							
**About the facilitators**	**Strongly disagree**	**Disagree**	**Slightly disagree**	**Not sure**	**Slightly agree**	**Agree**	**Strongly agree**
Facilitator A was able to deal well with emotions.	1	2	3	4	5	6	7
Facilitator A is a good listener and refrained from giving advice.	1	2	3	4	5	6	7
Facilitator A can connect well with people.	1	2	3	4	5	6	7
Facilitator A can work well cross-culturally.	1	2	3	4	5	6	7
Facilitator A has good organizational skills.	1	2	3	4	5	6	7
Facilitator A shows self-compassion.	1	2	3	4	5	6	7
Facilitator A did not try to fix me.	1	2	3	4	5	6	7
Facilitator A was knowledgeable and answered questions satisfactorily.	1	2	3	4	5	6	7
Facilitator A was approachable and friendly.	1	2	3	4	5	6	7

PARTICIPANT WORKSHOP FEEDBACK FORM
Please choose one response to each question
Possible responses:
1 = Strongly disagree; 2 = Disagree; 3 = Slightly disagree; 4 = Not sure; 5 = Agree a little; 6 = Agree; 7 = Strongly Agree
Or write responses where indicated

About the organization of the workshop	Strongly disagree	Disagree	Slightly disagree	Not sure	Slightly agree	Agree	Strongly agree
Facilitator A explained the exercises and information satisfactorily.	1	2	3	4	5	6	7
Facilitator A kept good time and ensured that the workshop ran smoothly.	1	2	3	4	5	6	7
Please give any further feedback about the facilitator(s):							

About the workshop information and exercises	Strongly disagree	Disagree	Slightly disagree	Not sure	Slightly agree	Agree	Strongly agree
Which exercises worked well? Why?							
Which exercises did not work well? Why?							
The workshop delivered useful information and skills to help address self-stigma.	1	2	3	4	5	6	7
